# The influence of circulating cholesterol and its components in middle-aged adults on cognitive function at mid- and later-life; a systematic review

**DOI:** 10.3389/fragi.2025.1430382

**Published:** 2025-03-19

**Authors:** O. C. Joyce, C. McHugh, D. Mockler, F. Wilson, Á. M. Kelly

**Affiliations:** ^1^ Discipline of Physiology, School of Medicine, Trinity Biomedical Sciences Institute and Trinity College Institute of Neuroscience, Trinity College Dublin, Dublin, Ireland; ^2^ John Stearne Library, St James’s Hospital, Dublin, Ireland; ^3^ Discipline of Physiotherapy, School of Medicine, Trinity Centre for Health Sciences, St James’s Hospital, Dublin, Ireland

**Keywords:** cholesterol, cognition, midlife, middle-aged, hypercholesterolemia

## Abstract

**Introduction:**

Several measures of cardiovascular health have been investigated as potential risk factors for development of cognitive decline in mid-to later-life, among them, circulating cholesterol. However, the efficacy of midlife interventions aimed at reducing blood cholesterol to mitigate the risk of cognitive decline is uncertain, with conflicting evidence reported from a range of longitudinal and cross-sectional studies. This review systematically investigates the connection between cholesterol measures in midlife and their impact on cognitive function in both mid- and later-life.

**Methods:**

Electronic databases were explored from their inception until December 2023. Studies that evaluated the relationship between cholesterol and its sub-components in midlife (40–65 years) and cognitive function in mid and/or later-life were included. Qualitative analysis was used to assess the associations between cholesterol and cognition according to cognitive domains (positive, negative, or neutral).

**Results:**

106 studies were included. We found inconsistent reporting on the association between midlife cholesterol and its sub-components, and cognitive function in older age. Longitudinal cohort studies (75%) generally showed no significant link between midlife cholesterol metrics and later-life cognitive domains. Conversely, half of individual cohort studies (50%) reported negative associations with memory, executive function, global cognition, and psychomotor speed. Most studies (78.6%) found no clear relationship between midlife cholesterol metrics and cognitive function either at midlife or later life, irrespective of study design or quality.

**Discussion:**

Our review found no conclusive link between midlife cholesterol and cognitive function in mid- and later-life, contrasting with the recent inclusion of high-LDL cholesterol as a modifiable risk factor for dementia by the 2024 Lancet Commission, following its exclusion in 2020 due to lack of evidence. These conflicting reports highlight the need to continue to investigate the importance of cholesterol metrics at midlife on cognitive function throughout the lifespan. Meanwhile, efforts to manage the all of cognitive decline in mid- and later-life across the population should continue to focus on other modifiable variables.

**Systematic Review Registration:**

https://www.crd.york.ac.uk/PROSPERO/view/CRD42021238293.

## Introduction

The increasing aging population is a major global public health challenge, not least due to the link between aging and cognitive decline, Alzheimer’s Disease (AD) (WHO, 2022; WHO, 2014) and other forms of dementia (Lee et al., 2018). The recent Lancet Commission on dementia prevention, intervention, and care suggests that up to 45% of all dementia cases are linked to modifiable risk factors (Livingston et al., 2024). Among these are risk factors of cardiovascular (CV) disease, such as diabetes, obesity, and hypertension, which have been strongly linked to lower cognitive function with advancing age (Iadecola et al., 2016; Arvanitakis et al., 2004; Angoff et al., 2022). Notably, high LDL cholesterol was added as a new modifiable risk factor for all-cause dementia identified by the Lancet Commission, following its omission in the 2020 report due to lack of evidence (Livingston et al., 2020). This result builds on the literature reporting a link between cholesterol and the development of neurodegenerative disorders associated with cognitive decline (Peters et al., 2020; Anstey et al., 2014; Anstey et al., 2017). However, impaired cognitive performance at midlife does not necessarily result in a dementia diagnosis (Pandya et al., 2016), likewise many live with mild cognitive impairment in old age that never progresses to dementia. Therefore understanding the impact of cholesterol metrics at midlife on general cognitive function both at midlife and in older age is of clinical relevance.

Cholesterol is required for healthy neuronal structure, function, and metabolic activity. However, high cholesterol during midlife can induce pathogenic risk factors leading to metabolic dysregulation (Kivipelto et al., 2001; Kivipelto et al., 2006) and a possible link with cognitive decline in later life has been suggested (Bennett et al., 2006). Total cholesterol (TC) encompasses high-density lipoprotein (HDL) “good cholesterol” and low-density lipoprotein (LDL) “bad cholesterol”, among other subcomponents. Elevated LDL can lead to reduced blood flow to the brain and increased oxidative stress (Matsuda and Shimomura, 2013), potentially increasing the risk of cognitive impairment, AD and vascular dementia with age. Further, dyslipidaemia has been linked to the development of neuropathological states from midlife onwards with higher levels of TC predictive of reduced cognitive function and capacity (Solomon et al., 2009), correlating to a 27% increased risk of dementia in later-life (Whitmer et al., 2005). Conversely, a moderate decrease in TC from midlife onwards is correlated with a 3.5-fold increase in the risk of cognitive impairment after more than two-decades, suggestive of a protective effect in older age (Solomon et al., 2007). Therefore, the impact of TC on the trajectory of age-related cognitive decline is potentially more pronounced in midlife (van Vliet, 2012; van Vliet et al., 2009). However, the evidence is conflicting; while many studies report elevated TC at midlife as a significant vascular risk factor and predictor for early onset dementia and cognitive decline (Whitmer et al., 2005; Solomon et al., 2007; Kalmijn, 2000; Debette et al., 2011; Mielke et al., 2010; Solomon et al., 2013), others have reported no effect (Mielke et al., 2010) or even a positive effect on cognitive function (van den Kommer et al., 2012; Lv et al., 2016).

While the evidence suggests a potential relationship between cholesterol, its subcomponents (LDL, HDL, and triglycerides) and cognitive function with age, there is no clear consensus as to whether intervention and management of cholesterol at midlife reduces risk of cognitive decline, in contrast to a dementia diagnosis either at midlife or later in life (Beker et al., 2021; Beker et al., 2020; Anatürk et al., 2020). Therefore, the primary aim of this review was to systematically determine the relationship between measures of cholesterol, triglycerides, and metabolic disorders of cholesterol measured at midlife and cognitive function in later-life, with a secondary aim to investigate the relationship between measures of cholesterol at midlife and cognitive function at midlife.

## Methods

This review followed the Preferred Reporting Items for Systematic Reviews (PRISMA; www.prisma-statement.org) and Meta-Analyses standards and was submitted to PROSPERO, a systematic review registry. This review’s registration may be found at https://www.crd.york.ac.uk/prospero/ (registration number: CRD42021238293). This review is a sub-analysis of the overall registered review on midlife cardiovascular risk factors and cognitive health at midlife and later-life (Joyce et al., 2023).

### Search strategy and screening

**FIGURE 1:**
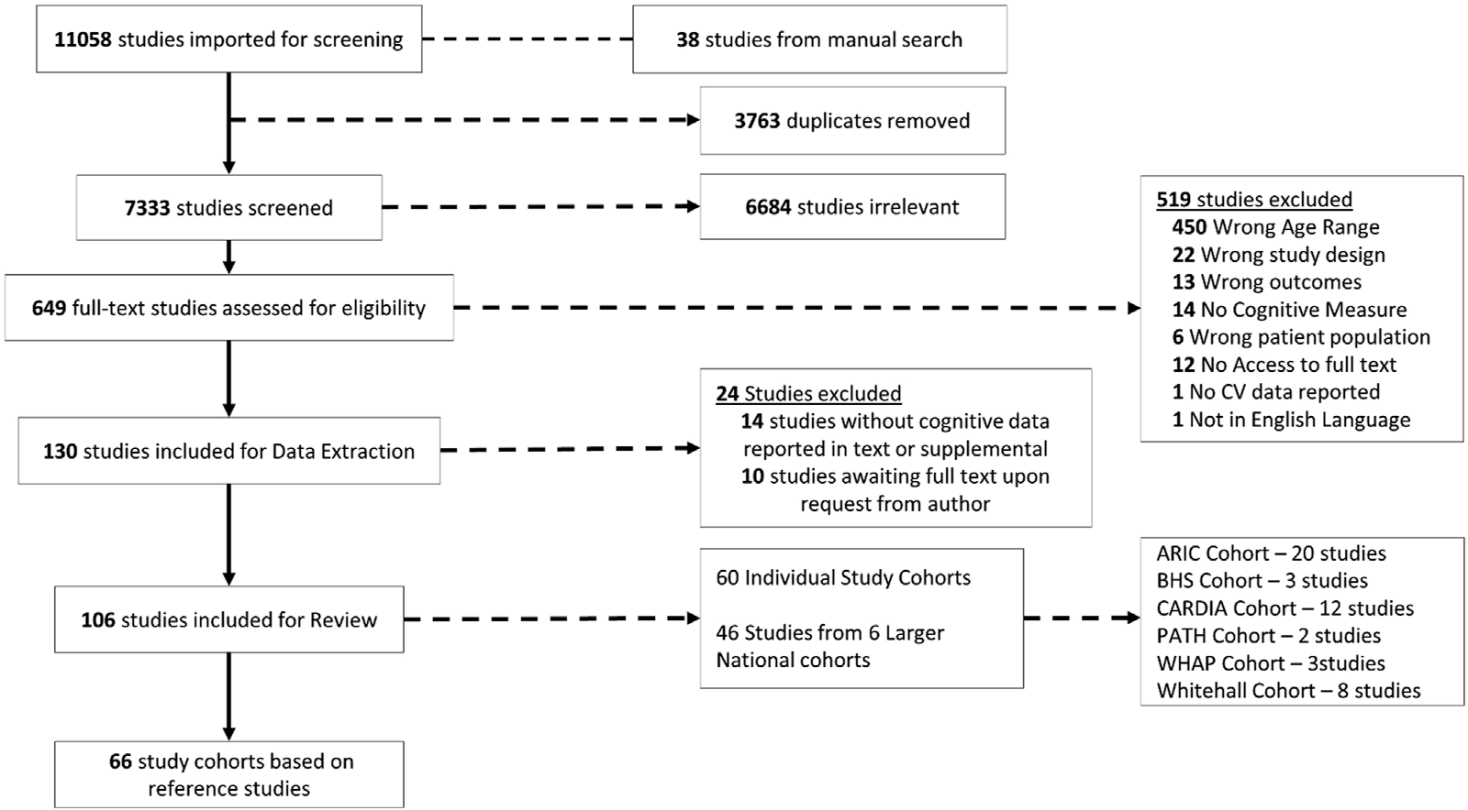
Flow chart of the study selection process.

The online databases EMBASE, MEDLINE, PubMed, Web of Science, and CINAHL from their inception until December 2023 were searched using “*cholesterol*” and a combination of keywords and Mesh terms for cognition, midlife, and later-life. The full search strategy is outlined in Supplementary Appendix A1. There were no language or date constraints applied. A manual search of the reference lists of included studies was conducted to identify any additional relevant material. Figure 1 depicts the search methodology’s step-by-step approach. Screening of studies was conducted independently by two reviewers (O.C.J and C. McH) using Covidence (https://www.covidence.org/home), including title/abstract and full text screening. Disagreements between reviewers were resolved through discussion. A third reviewer was consulted if consensus could not be reached (Á.M. K and F.W.).

### Eligibility criteria

Studies deemed eligible included those with human participants, cholesterol metrics at *midlife* (adults aged between 40–65 years, defined by World Health Organisation [WHO]), later-life (adults aged >65-years old, defined by WHO), or both to establish the longitudinal relationships between midlife cholesterol and later-life cognition. The cholesterol metrics included total cholesterol (TC), high-density lipoprotein cholesterol (HDL-C), low-density lipoprotein cholesterol (LDL-C), and triglycerides. Studies were included if they reported at least one of these metrics measured during midlife. Inclusion was not contingent on reporting all cholesterol metrics. Some studies reported a single metric, while others included multiple metrics, providing varying levels of raw data or statistical findings. Metrics were assessed against standard clinical reference values for interpretation, such as LDL-C levels being categorized as optimal (<100 mg/dL), near-optimal (100–129 mg/dL), borderline-high (130–159 mg/dL), high (160–189 mg/dL), and very high (>190 mg/dL). Studies without raw data were included if their results provided interpretable statistical outcomes within the defined cholesterol and cognitive frameworks. Cognition was divided into several domains, including but not limited to memory, attention, executive function, global cognition, and intelligence (see Table 2; Supplemental Table B3). Studies were excluded if cognitive testing was done by a proxy or designated respondent who was not the participant, such as a friend or family member, if a pre-existing diagnosis of dementia or any other form of cognitive impairment was an inclusion factor, or if studies of specific disabilities were linked to modifiable behavioural risk factors including but not limited to physical inactivity, unhealthy diet and the harmful use of alcohol.

### Data extraction

Data extraction was carried out in accordance with the STROBE guidelines (Von Elm et al., 2014) using Endnote version 20 and Microsoft Excel. Studies were assigned a reference number and data including study aims, participant characteristics, measures of cognition and cholesterol alongside relevant outcome data, such as group means, standard deviation (SD), standard error (SE), statistical significance, and precision estimates were extracted. For multiple articles identified from a single study, preference was given to the most recent publication with the longest follow-up period or the most comprehensive reporting of relevant data, thus only one article from each larger cohort study was included. Of the 106 studies included in the review, 46 originated from larger national cohort studies. From these, six unique studies were included in the analysis to avoid duplication. The follow-up durations among the included longitudinal studies varied significantly, reflecting differences in study designs; these are summarized in Table 4 and Supplemental Table B2. Where data provided in the study were not sufficient, authors were contact for further information. The data extraction pro-forma was piloted to ensure correct reporting. Cognitive outcomes were grouped according to a relevant domain (see Table 1.). Each test was interpreted according to its predefined scoring system, as used by the authors of the original studies, to categorize cognitive performance across domains. Grouping similar tests by cognitive domain enabled meaningful comparisons across studies, despite variability in individual test designs. For studies using distinct tests, results were categorized by domain to minimize heterogeneity. While exact score harmonization across all tests was not feasible, this approach maintained methodological rigor and ensured robust inference of the relationships between cholesterol metrics and cognitive performance.

**TABLE 1:** Summary table of weighted average for all cognitive measures and associated cholesterol metrics at baseline (i.e., midlife).

Cognitive Variable	No. of Studies (n =)	Weighted Average (Mean ±SD)	Age (Mean ±SD; Years)	TC (mg/dL)	HDL-C (mg/dL)	LDL-C (mg/dL)	TG (mg/dL)
Memory Verbal Memory	Total: n= 10 **Immediate**: n= 1, **Delayed**: n= 3, **EBM**: n= 1, **RAVLT (Immediate &** **Delayed recall):** n= 1, **RAVLT (Learning Score):** n= 1, **RAVLT (Summary Metric):** n= 3 **SRT:** n= 1, **ROCF (Immediate &** **Delayed recall):** n= 1, **CERAD (Immediate & Delayed):** n= 1, **CVLT (Immediate & Delayed):** n= 1	**Immediate:** Total = 14.5 **Delayed:** Total = 6.9 ± 1.5 **EBM**: Total = 10 **RAVLT (Immediate & Delayed recall):** Total = 7.1 ± 2.7, 7.1 ± 2.8 **RAVLT (Learning Score):** Total = 36.8 ± 8.3 **RAVLT (Summary metric):** Total = 8.9 ± 3.1 **SRT:** Total = 34.3 **ROCF (Immediate &** **Delayed recall):** 16.1 ± 7.6, 14.9 ± 7.8 **CERAD (Immediate & Delayed):** Total = 7.2 ± 1.1, 7.7 ± 1.5 **CVLT (Immediate & Delayed):** Total = 8.8 ± 2.1, 8.8 ± 3.2	Total = 50.9 ± 4.7, Males = 56.6 ± 7.1, Females = 56.2 ± 7.1	Total = 102.8 ± 17.9, Males = 104.4 ± 18.2, Females = 104.6 ± 18.9 **Delayed:** Total = 81.2, Males = 104.4 ± 18.2, Females = 104.6 ± 18.9 **Immediate:** Total = 81.2 **EBM:** Not available **RAVLT (Summary metric):** Total = 90.6 ± 17.3 **RAVLT (Immediate & Delayed recall, Learning Score):** Total = 219.4 ± 39.7 **SRT:** Total = 106.4 ± 19.4 **ROCF (Immediate &** **Delayed recall):** Total = 219.4 ± 39.7 **CERAD (Immediate & Delayed):** Total = 104.9 ± 18.9 **CVLT (Immediate & Delayed):** Total = 104.9 ± 18.9	Total = 27.8 ± 6.6 **Delayed:** Total = 22.3 **Immediate:** Total = 22.3 **EBM:** Not available **RAVLT (Summary metric):** Total = 34.8 ± 9.5 **RAVLT (Immediate & Delayed recall, Learning Score):** Total = 45.3 ± 6.6 **SRT:** Total = 23.2 ± 5.4 **ROCF (Immediate &** **Delayed recall):** Total = 45.3 ± 6.6 **CERAD (Immediate & Delayed):** Total = 27.9 ± 7.56 **CVLT (Immediate & Delayed):** Total = 27.9 ± 7.56	Total = 74.7 ± 16.8 **Delayed:** Not available **Immediate:** Total = 49.86 **EBM:** Not available **RAVLT (Summary metric):** Total = 80.9 ± 12.1 **RAVLT (Immediate & Delayed recall, Learning Score):** Total = 146.6 ± 35.2 **SRT:** Total = 72.9 ± 18.2 **ROCF (Immediate &** **Delayed recall):** Total = 146.6 ± 35.2 **CERAD (Immediate & Delayed):** Not available **CVLT (Immediate & Delayed):** Not available	Total = 31.2 ± 16.6 Delayed: Not available Immediate: Total = 27 EBM: Not available RAVLT (Summary metric): Total = 20.9 ± 0.5 RAVLT (Immediate & Delayed recall, Learning Score): Total = 184.7 ± 104.7 SRT: Total = 23.6 ± 14.7 ROCF (Immediate & Delayed recall): Total = 184.7 ± 104.7 CERAD (Immediate & Delayed): Total = 23.9 ± 15.3 CVLT (Immediate & Delayed): Total = 23.9 ± 15.3
Episodic Memory	Total: n= 2	Total = 5.4 ± 0.2	Total = 49.8 ± 8, Males = 50.3 ± 8, Females = 51 ± 8.1	Total = 118.7 ± 14.3	Not available	Not available	Not available
Semantic Memory	Total: n= 1	Total = 15.6 ± 2.9 Males = 15.2 ± 3.0, Females = 15.9 ± 2.8	Total = 50.7 ± 8, Males = 50.3 ± 8, Females = 51 ± 8.1	Not available	Not available	Not available	Not available
Working Memory	Total: n= 16 **DSST:** n= 5 **CMS Score:** Total: n= 1 **DSB Test**: n= 8 **MIS (MoCA):** n= 1 **VRT**: n= 1 **LMT:** n = 2	**DSST:** Total = 45.5 ± 8.5 **CMS Score:** Total = 76.6 ± 12.9 **DSB Test:** Total = 6.2 ± 1.9 **MIS (MoCA):** Total = 12.7 ± 2.4 **VRT:** Total = 11.3 **LMT:** Total = 10.1	Total = 52.02 ± 4.4	Total = 89.4 ± 16.4 **DSST:** Total = 79.3 ± 18.4, Males = 111.96 ± 19.3, Females = 124.05 ± 25.4 **CMS Score:** 81.1 ± 11.6 **DSB Test:** Total = 144.7 ± 23.6 **MIS (MoCA):** Not available **VRT:** Total = 106.4 ± 19.4 **LMT:** Total = 83.9 ± 0.7	Total = 26.93 ± 5.6 **DSST:** Total = 28.3 ± 8.7, Males = 23.8 ± 6.3, Females = 28.9 ± 7.7 **CMS Score:** 47.16 ± 13.3 **DSB Test:** Total = 108.01 ± 21.6 **MIS (MoCA):** Not available **VRT**: Total = 23.2 ± 5.4 **LMT:** 22.9	Total = 67.9 ± 13.0 **DSST**: Not available **CMS Score:** Total = 47.2 ± 13.3 **DSB Test:** Total = 108.01 ± 21.6 **MIS (MoCA):** Not available **VRT:** Total = 72.9 ± 18.2 **LMT:** 49.9	Total = 30.1 ± 13.2, Males = 30.06 ± 17.5, Females = 26.8 ± 14.2 DSST: Total = 28.08 ± 15.84, Males = 30.06 ± 17.5, Females = 26.8 ± 14.2 CMS Score: Total = 28.9 ± 14.1 DSB Test: Total = 88.7 ± 43.6 MIS (MoCA): Not available VRT: Total = 23.6 ± 14.7 LMT: Total = 26.3
Attention	Total: n= 9 **TMT-A:** n= 5 **CRT**: n= 3 **SiRT:** n= 2 **DSF Test:** n= 5 **AI (MoCA):** n = 1	**TMT-A:** Total = 42.2 ± 30.9 **CRT:** Total = 783.1 ± 167.5 **SiRT:** Total = 250.9 ± 56.7 **DSF Test:** Total = 9.4 ± 1.5 **AI (MoCA):** 16.3 ± 1.8	Total = 49.9 ± 4.6, Males = 48.1 ± 5.2, Females = 48.1 ± 5.5	Total = 109.6 ± 16.9 **TMT-A:** Total = 142.1 ± 22.9 **CRT:** Total = 123.8 ± 23.1 **SiRT:** Total = 101.8 ± 19.3 **DSF Test:** Total = 117.8 ± 14.3 **AI (MoCA):** Not available	Total = 29.4 ± 5.3 **TMT-A:** Total = 39.3 ± 7.1 **CRT:** Total = 32.02 ± 7.3 **SiRT:** Total = 28.9 ± 6.7 **DSF Test:** Total = 29.9 ± 3.3 **AI (MoCA):** Not available	Total = 70.7 ± 14.2 **TMT-A:** Total = 117.1 ± 23.9 **CRT:** Total = 62.8 ± 16.02 **SiRT:** Total = 62.8 ± 16.02 **DSF Test:** Total = 81.8 ± 11.6 **AI (MoCA):** Not available	Total = 41.9 ± 12.9 TMT-A: Total = 88.7 ± 43.6 CRT: Total = 51.5 ± 20.1 SiRT: Total = 20.3 ± 0.3 DSF Test: Total = 65.3 ± 26.5 AI (MoCA): Not available
Intelligence	Total: n= 5 **IQ**: n= 3 **MR:** n= 1 **AFQT:** n = 1	**IQ:** Total = 102.3 ± 10.6 **MR:** Total = 18.1 **AFQT:** Total = 61.8 ± 0.9	Total = 54.8 ± 3.9	Total = 82.7 ± 12.03 **IQ:** Total = 94.7 ± 15.5 **MR:** Not available **AFQT:** Not available	Total = 27.3 ± 6.7 **IQ:** Total = 49.9 ± 13.1 **MR**: Not available **AFQT:** Not available	Total = 53.6 ± 14.9 **IQ:** Total = 126.6 ± 32.3 **MR:** Not available **AFQT:** Not available	Total = 40.2 ± 19.2 IQ: Total = 168.6 ± 77.3 MR: Not available AFQT: Not available
Executive Function Letter Cancellation	Total: n= 2 **LSST**: n= 1 **LCCS**: n= 1	**LSST:** Total = 282 **LCCS:** Total = 50 ± 7.3	Total = 52.7 ± 2.6	Total = 102.4 ± 21.6 **LSST:** Total = 102.4 ± 21.6 **LCCS:** Not available	Total = 40.7 ± 10.6 **LSST:** Total = 29.2 ± 7.4 **LCCS:** Total = 64.7 ± 17.1	Total = 85.1 ± 21.9 **LSST:** Total = 63.4 ± 18 **LCCS:** Total = 128.9 ± 29.7	Total = 43.5 ± 16.9 LSST: Total = 19.8 LCCS: Total = 91.8 ± 51.4
Verbal Fluency	Total: n= 6 **WFT**: n= 4 **VIS (MoCA):** n= 1 **BeDT:** n= 1 **BuDT:** n= 1	**WFT:** Total = 31.5 ± 8.4 **VIS (MoCA):** Total = 6.48 ± 0.92 **BeDT:** Male = 12, Female: 12 **BuDT:** Male = 7, Female: 6	Total = 54.2 ± 5.6, Male = 56.6 ± 7.1, Female = 56.2 ± 7.1	Total = 148.1 ± 29.5, Male = 104.4 ± 18.8, Female = 104.6 ± 18.9 **WFT:** Total = 148.1 ± 29.5, Male = 104.4 ± 18.8, Female = 104.6 ± 18.9 **VIS (MoCA):** Not available **BeDT:** Not available **BuDT:** Not available	Total = 43.3 ± 13.5 **WFT**: Total = 43.3 ± 13.5 **VIS (MoCA):** Not available **BeDT**: Not available **BuDT:** Not available	Total = 72.9 ± 18.2 **WFT**: Total = 72.9 ± 18.2 **VIS (MoCA):** Not available **BeDT**: Not available **BuDT:** Not available	Total = 23.6 ± 14.7 WFT: Total = 23.6 ± 14.7 VIS (MoCA): Not available BeDT: Not available BuDT: Not available
Processing Speed	Total: n= 14 **TMT-B:** n= 8 **STIT:** n= 3 **WMT:** n= 1 **CES**: n= 2 **RVP (CANTAB):** n= 1 **SCWT:** n= 1 **EIS (MoCA):** n= 1 **LT**: n= 1	**TMT-B:** Total = 94.4 ± 5.5 **STIT:** Total = 42.9 ± 1.5 **WMT:** Total = 2.3 ± 1.1 **CES:** Total = 48.9 ± 0.1 **RVP (CANTAB):** Total = 0.9 **SCWT:** Total = 19.1 **EIS (MoCA):** Total = 11.6 ± 1.4 **LT:** Male = 39.8 ± 17.8, Female = 45.5 ± 26.6	Total = 53.03 ± 4.7, Male = 56.6 ± 7.1, Female = 53.2 ± 4.8	Total = 92.8 ± 18.2, Male = 104.4 ± 18.8, Female = 104.4 ± 18.5 **TMT-B:** Total = 89.5 ± 18.8, Female = 104.04 ± 17.1 **STIT:** Total = 214.9 ± 38.9, Male = 104.4 ± 18.8, Female = 104.6 ± 18.9 **WMT:** Total = 200.05 ± 25.6 **CES:** Total = 121.3 ± 12.6 **RVP (CANTAB):** Total = 99 **SCWT:** Male = 104.4 ± 18.2, Female: 104.6 ± 18.9 **EIS (MoCA):** Not available **LT:** Not available	Total = 24.8 ± 5.8, Female = 28.1 ± 7.6 **TMT-B:** Total = 24.7 ± 5.8, Female = 28.1 ± 7.6 **STIT**: Total = 45.3 ± 10.01 **WMT:** Total = 49.5 ± 7.6 **CES:** Not available **RVP (CANTAB):** Not available **SCWT:** Not available **EIS (MoCA):** Not available **LT**: Not available	Total = 77.1 ± 19.2, Female = 67.5 ± 16.4 **TMT-B**: Total = 76.7 ± 19.1, Female = 67.5 ± 16.4 **STIT:** Total = 146.6 ± 35.2 **WMT:** Total = 127.9 ± 28.3 **CES**: Not available **RVP (CANTAB):** Not available **SCWT**: Not available **EIS (MoCA):** Not available **LT:** Not available	Total = 32.9 ± 19.04 TMT-B: Total = 32.4 ± 18.9 STIT: Total = 184.7 ± 104.7 WMT: Total = 113.3 ± 31.4 CES: Not available RVP (CANTAB): Not available SCWT: Not available EIS (MoCA): Not available LT: Not available
Global Cognition	Total: n= 22 **MMSE:** n= 14 **MoCA:** n= 7 **IQCODE:** n= 1 **CAMCOG:** n= 1 **NART:** n= 1 **IST:** n= 1 **BPP:** n= 1 **ACE:** n= 1 **HRS-CS:** n= 1 **GCA:** n = 1	**MMSE**: Total = 27.7 ± 0.7 **MoCA**: Total = 25.3 ± 3.1, Male = 25 ± 2.9, Females = 25.5 ± 2.9 **IQCODE**: Total = 43.4 ± 3.01 **CAMCOG:** Total = 90 **NART:** Total = 28 **IST:** Total = 32.4 **BPP:** Total = 46.9 **ACE:** Total = 94.9 **HRS-CS:** Total = 14.31 ± 4.06, Male = 14.2 ± 4.15, Female = 14.44 ± 3.96 **GCA:** Male = 53.1 ± 10.7, Female = 51.7 ± 10.5	Total = 54.6 ± 5.3, Males = 63.9 ± 0.7, Females = 63.9 ± 0.6	Total = 98.3 ± 14.1 **MMSE:** Total = 103.6 ± 15.9 **MoCA:** Total = 86.3 ± 9.7 **IQCODE:** Total = 200.3 ± 35.21 **CAMCOG**: Not available **NART:** Not available **IST**: Total = 99 **BPP:** Total = 99 **ACE:** Total = 99 **HRS-CS:** Not available **GCA:** Total = 119.2 ± 23.9, Males = 111.9 ± 19.3, Females = 124.02 ± 25.4	Total = 28.3 ± 6.7, Males = 22.9 ± 3.9, Females = 27.8 ± 5.3 **MMSE:** Total = 24.4 ± 6.4 **MoCA**: Total = 25.7 ± 4.6 **IQCODE**: Total = 200.3 ± 35.2 **CAMCOG**: Total = 23.4 **NART:** Total = 23.4 **IST:** Not available **BPP:** Not available **ACE:** Not available **HRS-CS:** Not available **GCA:** Total = 26.8 ± 7.6, Males = 23.8 ± 6.3, Females = 28.9 ± 7.7	Total = 63.4 ± 12.6 **MMSE:** Total = 69.7 ± 17.9 **MoCA**: Total = 49.6 ± 0.9 **IQCODE**: Not available **CAMCOG**: Not available **NART:** Not available **IST:** Not available **BPP:** Not available **ACE:** Not available **HRS-CS:** Not available **GCA:** Not available	Total = 26.1 ± 8.2, Males = 29.6 ± 11.2, Females = 25.8 ± 9.9 MMSE: Total = 25.8 ± 9.9 MoCA: Total = 26.3, Males = 28.8, Females = 23.4 IQCODE: Not available CAMCOG: Total = 27 NART: Total = 27 IST: Not available BPP: Not available ACE: Not available HRS-CS: Not available GCA: Total = 28.1 ± 15.8, Males = 30.1 ± 17.5, Females = 25.8 ± 9.9
Inductive Reasoning	Total: n= 2	**AH-4:** Total = 73.1, Male = 49.2 ± 9.5, Female = 42.9 ± 11.6	Total = 52.8, Males = 55.1 ± 5.9, Females = 55.3 ± 5.9	Male= 227.5 ± 39.1, Female = 230.9 ± 41.3	Total = 23.4 ± 10.3, Male= 53 ± 13.2, Female = 65 ± 16.6	Not available	Total = 27
Psychomotor Speed	Total: n= 5	**SDMT:** Total = 43.4 ± 1.7, Female = 50.5	Total = 48.4 ± 2.6, Females = 50.1 ± 2.6	Total = 89.02 ± 4.4, Female = 104.04 ± 17.1	Total = 24.5 ± 0.3, Female = 28.08 ± 0.4	Total = 55.5 ± 4.1, Female = 67.5 ± 16.4	Total = 26.5 ± 0.4
Visuospatial Organisation	Total: n= 4 **BDT:** n= 1 **VIS MoCA:** n= 1 **Vr-D:** n = 1 **SCS:** n = 1	**BDT:** Total = 25.6 ± 0.6 **VIS MoCA:** Total = 6.48 ± 0.92 **Vr-D:** Total = 8.62 ± 3.24 **SCS:** Male = 53.6 ± 10.7, Female = 49.2 ± 9.9	Total = 46.5 ± 8.7	Total = 115.5 ± 20.4, Male = 111.9 ± 19.3, Female = 124.02 ± 25.4 **BDT:** Total = 96.8 ± 1.9 **VIS MoCA:** Not available **Vr-D:** Not available **SCS:** Total = 119.2 ± 23.9, Male = 111.9 ± 19.3, Female = 124.02 ± 25.4	Total = 26.9 ± 6.5, Male = 23.8 ± 6.3, Female = 28.9 ± 7.7 **BDT:** Total = 27.6 ± 1.5 **VIS MoCA:** Not available **Vr-D:** Not available **SCS:** Total = 26.8 ± 7.6, Male = 23.8 ± 6.3, Female = 28.9 ± 7.7	Total = 58.9 ± 1.4 **BDT:** Total = 63.02 ± 1.5 **VIS MoCA:** Not available **Vr-D:** Total = 48.2 ± 1.3 **SCS:** Not available	Total = 27.5 ± 13.6 BDT: Total = 24.3 ± 2.2 VIS MoCA: Not available Vr-D: Not available SCS: Total = 28.1 ± 15.8, Male = 30.1 ± 17.5, Female = 26.8 ± 14.2
Temporal Orientation	Total: n = 2	Total = 6.6 ± 0.9	Total = 54.5 ± 7.1	Total = 207.1 ± 61.5	Not available	Not available	Not available

Abbreviations: ACE, Addenbrooke’s cognitive examination; AH-4, Alice Heim 4-I; BDT, Block Design Test; BeDT, Benson Delay Test; BNT: Boston Naming Test; BP, Blood Pressure; BPP, Børge Priens Prøve; BuDT, Buschke Delay Test; CAMCOG, Cambridge Cognition Examination; CANTAB, Cambridge Neuropsychological Test Automated Battery; CDT, Clock Drawing Test; CERAD, Consortium to Establish a Registry for Alzheimer’s Disease; CMS, Chinese Clinical Memory Scale; CRT, Choice Reaction Time; CVLT, California Verbal Learning Test; DSB, Digit Span Backwards, CES, Composite Executive Score; DSF, Digit Span Forward; DSST, Digit Symbol Substitution Test; EBM, East Boston Memory Test; EIS, Executive Index Score; GCA, General Cognitive Ability; HRS-CS, U.S. Health and Retirement Study Composite Score; IST, Intelligenz-Struktur-Test; IQCODE, Informant Questionnaire on Cognitive Decline in the Elderly; IQ, Intelligence Quotient; LCCS, Letter Cancellation Composite Score; LSST, Letter Search Speed Test; LT, Labyrinth Test; McNS, McNair Survey; MINT, Multilingual Naming Test; MIS, Memory Index Score; MoCA, Montreal Cognitive Assessment; MMSE, Mini-Mental State Exam; MR, Mental Rotation Test; MVT, Mill Hill Vocabulary Test; NART, National Adult Reading Test; PFT, Phonemic Fluency Test; RAVLT, Rey Auditory Verbal Learning Test; ROCF, Rey–Osterreith complex figure; RVP, Rapid Visual Processing; SCWT, Stroop Colour Word Test; SDMT, Symbol Digits Modalities Test; SCS, Spatial Composite Score; SFT, Semantic Fluency Test; SRT, Selective Reminding Test; SiRT, Simple Reaction Time; STIT, Stroop Test (Interference Time); STW, Spot the Word Test; TMT-A, Trail making Test Part A; TMT-B, Trail making Test Part B; TrB-A, Trail making Test Difference between Part B and A; VIS, Visuospatial Index Score; VRT, Visual Reproduction Test; VSS, Visual Search Speed; WFT, Word Fluency Test; WDS, WAIS-IV Digit Sequencing; WAIS, Wechsler Adult Intelligence Scale; WMT, Word Matching Test; 5-CMT, - Choice Movement Test.

### Risk of bias and methodological assessment

The Appraisal Tool for Cross Sectional Research (AXIS) was used to assess the methodological quality of the included studies (Downes et al., 2016). As seen in our previous work (Joyce et al., 2023) this appraisal tool used a series of 20 questions to determine study quality and risk of bias by means of answering ‘Yes’, ‘No’ or ‘Unsure’. Answers were colour coded highlighting the impact of response towards overall bias and study quality: green, positive impact on quality of study; red, negative impact on quality of study; and amber, unknown impact on quality of study. Two reviewers (O.C.J. and C. McH.) independently evaluated the included studies. Disagreements between reviewers were resolved through discussion and a third reviewer (F.W. or Á.K.) was consulted if a consensus could not be reached. The quality of each study was rated as either low, moderate, or high.

### Statistical analysis

Descriptive analysis of cholesterol-related metrics were evaluated, including dyslipidaemia, hypercholesterolemia, and lipid-lowering medication use. The weighted mean of all cognitive measures (cognitive-specific domains and accompanying neuropsychological tests), TC, HDL-C, LDL-C, and triglyceride levels were calculated. Data are presented as mean and standard deviation or number and frequency. The weighted mean was calculated using the following formula:

$$\text{Weighted Average}=\frac{\sum_{i=1}^{n}X_{i}w_{i}}{\sum_{i=1}^{n}w_{i}}$$

where *xi* represents each individual value in the dataset; *wi* represents the weight corresponding to each value *xi*; and *n* is the total number of values in the dataset.

Qualitative analysis was used to assess the associations between midlife cholesterol and cognition at mid- and- later-life according to each cognitive domain; positive, negative, or neutral. The qualitative result from each included study was determined by the relationship identified by the authors. A meta-analysis was not deemed possible based on the extracted data for both mid- and later-life cognition.

## Results

### Literature search

Figure 1 displays an overview of the study selection. In all, 11,058 records were found during the initial search accompanied by 38 records from a manual search. Following removal of duplicates and title and abstract screening, 649 full texts were examined. As a result, 519 studies were excluded for reasons outlined in Figure 1. The authors of ten papers were contacted for access to full texts but were later removed owing to a lack of response. A total of 106 studies published between 1995 and 2024 were included. Of studies included, 46were from larger national cohorts, resulting in 6 reference studies and 60 were individual study cohorts. Subsequently, 66 study cohorts were included for demographics and weighted means of cholesterol and cognitive metrics.

### Methodological and risk of bias assessment

Overall, studies were deemed of moderate-to-high quality, with 19 of low-quality, 43 of moderate-quality, and 44 of high-quality. Unfavorable commonalities emerged across studies, including failure to justify sample size (n = 92), to classify non-responders (n = 91), to provide information regarding non-responders (n = 97), and to provide a clear assessment of statistical significance (n = 33) (Supplemental Table B1).

### Study characteristics

Of the 106 studies included, 90 included males and females, 9 assessed males only, and 7 assessed females only. Studies were conducted in 26 countries, including United States (n = 46), the United Kingdom (n = 12), China (n = 4), Australia (n = 6), and Italy (n = 4).

### Participant characteristics

A total of 203,845 participants with a weighted mean age of 54.8 ± 5 years were included. The mean weighted BMI was 27.3 ± 4.9 kg/m2. The average height and weight were 168.5 ± 1.6 cm and 79.3 ± 8.3 kg, respectively.

Studies that reported by sex (n = 60), included 82,661 males and 84,450 females. There was no discernible difference in age (57.5 ± 4.6 vs. 55.5 ± 4.1 years) or BMI (26.1 ± 2.5 vs. 26.3 ± 3.6 kg/m2), with males having a higher mean weight (88.4 vs. 78.6 kg) and height (165.8 ± 5.3 vs. 154.8 ± 4.9 cm) than females.

### Total cholesterol, HDL-C, LDL-C, triglycerides, and medication use

The weighted values for TC were 179.5 ± 34.4 (n = 56), HDL-C was 43.1 ± 13.7 (n = 44), LDL-C was 94.4 ± 26.9 (n = 27) and triglycerides was 84.7 ± 46.4 (n = 30). Dyslipidemia and hypercholesterolemia were reported in 6,290 and 18,572 participants across 10 and 12 studies, respectively (see Table 1).

**FIGURE 2:**
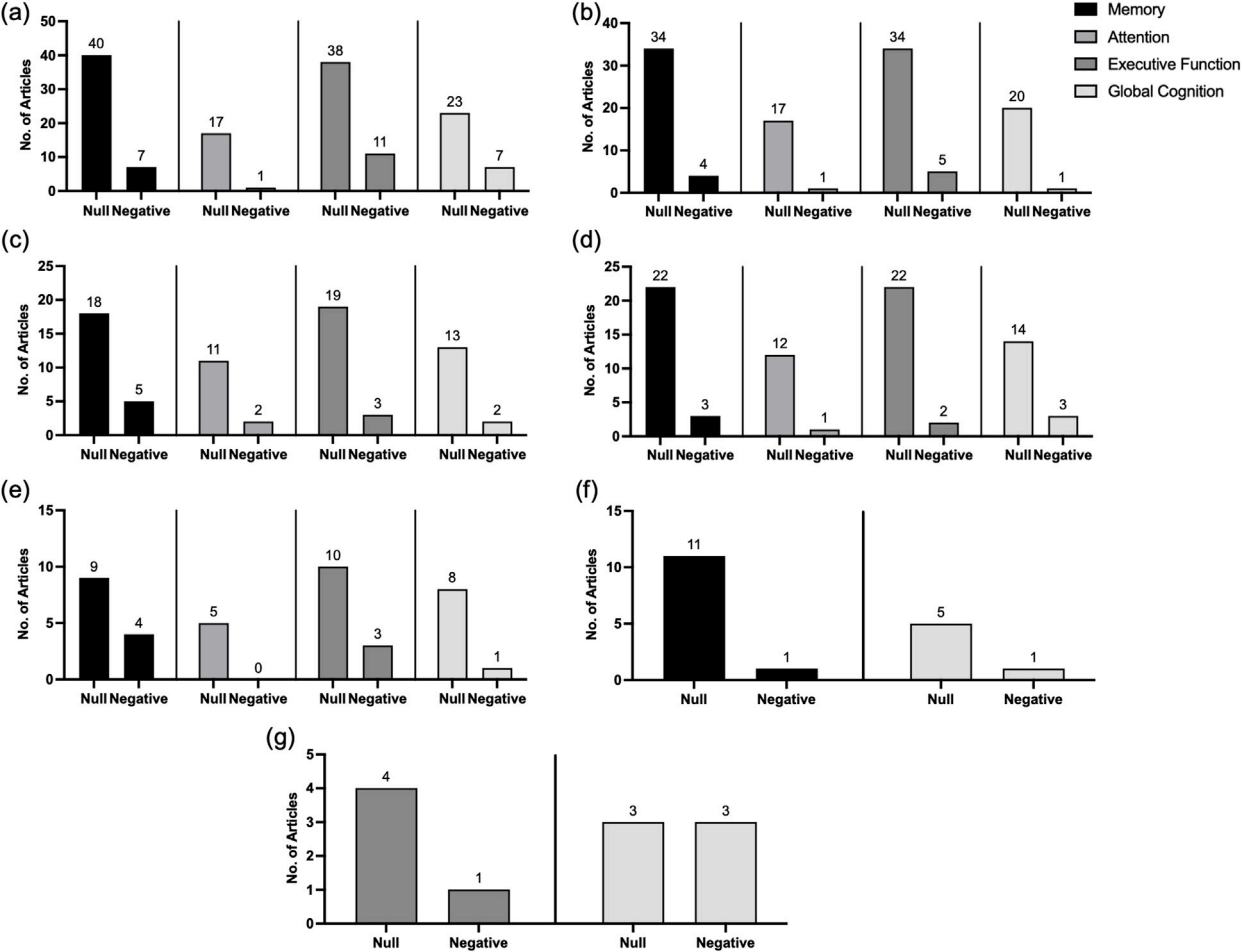
The null and negative relationships between cholesterol, its sub-components, and cognitive domains of memory, attention, executive function and global cognition at midlife. **(A)** TC, **(B)** HDL-C, **(C)** LDL-C, **(D)** TG, **(E)** Hypercholerolemia, **(F)** Lipid-lowering medication, **(G)** DyslipidemiaCreditValidation Error Authors: Oisin Joyce, Cliodhna McHugh, David Mockler, Fiona Wilson, Aine Kelly, please check and link manually.

In studies that reported by sex, males had higher mean weighted values for TC (180.1 ± 31.4 vs. 142.5 ± 25.6), HDL-C (48.9 ± 11.9 vs. 43.4 ± 9.7), LDL-C (125.9 ± 32.6 vs. 77.7 ± 19.4), triglycerides (69.4 ± 43.2 vs. 41 ± 27.6). A higher proportion of females were reported with dyslipidemia and hypercholesterolemia than males (1,202 vs. 1,026 and 902 vs. 894). Conversely, a higher number of males were reported as using lipid/cholesterol lowering medication than females (523 vs. 430) (see Tables 2, 3; Figure 2).

**TABLE 2:** Summary of weighted cholesterol metrics across included studies (reference studies applied).

	TC	No Studies (n=)	No. Participants (n=)	HDLC	No Studies (n=)	No. Participants (n=)	LDLC	No Studies (n=)	No. Participants (n=)	TG	No Studies (n=)	No. Participants (n=)
Total	179.5 ± 34.4	56	176,438	43.1 ± 13.7	44	108,811	94.4 ± 26.9	27	38,829	84.7 ± 46.4	30	61,340
Male	180.1 ± 31.4		5,908	48.9 ± 11.9		4,159	125.9 ± 32.6		298	69.4 ± 43.2		821
Female	142.5 ± 25.6		5,585	43.4 ± 9.7		3,701	77.7 ± 19.4		1,736	41.0 ± 27.6		1,941

**TABLE 3:** Summary of hypercholesterolremia, dyslipidemia, and lipid-cholesterol lowering medication across included studies (reference studies applied).

	Hypercholesterolemia (n=)	No Studies (n=)	Dyslipidemia (n=)	No Studies (n=)	Lipid/Cholesterol Lowering Medication (n=)	No Studies (n=)
Total	18,572	12	6,290	10	6,542	16
Male	894		1,026		523	
Female	902		1,202		430	

### Associations between cholesterol metrics at midlife and measures of cognition at later-life

Of the 6 longitudinal study cohorts, 4 included measures of midlife cholesterol and later-life cognition. A negative relationship was reported in a single study between executive function and TC, LDL-C, HDL-C and triglycerides, between memory and TC and triglycerides and between global cognition and TC, triglycerides (Power et al., 2018). The remaining three studies found no relationship between cholesterol metrics at midlife and later-life cognition (Szoeke et al., 2016; Tuligenga et al., 2014; Kaffashian et al., 2013).

Of the 60 individual study cohorts, 7 reported on the relationship between midlife cholesterol and later-life cognitive function. Four studies reported negative associations between TC and cognitive measures (memory, executive function, processing speed, and global cognition) (Kivipelto et al., 2001; An et al., 2019; Wendell et al., 2016; Henderson et al., 2003; Chuang et al., 2023) and for HDL-C, LDL-C and triglycerides and cognitive measures (memory, attention, global cognition, spatial ability, and perceptual speed) (An et al., 2019; Henderson et al., 2003; Chuang et al., 2023; Reynolds et al., 2010). A positive association between TC, HDL-C, and hypercholesterolemia and cognitive measures, including executive function, processing speed, global cognition, and verbal learning and memory was reported in three studies (An et al., 2019; Wendell et al., 2016; Yang et al., 2018).

There was no difference among all longitudinal studies in the reported relationship (null vs. negative vs. positive) across all cognitive domains assessed based on study design or quality with all included studies being of moderate (n = 6, 46%) to high (n = 7, 54%) quality (see Table 4).

**TABLE 4:** Summary of longitudinal studies with negative or null relationship between cholesterol and cognitive measures at later life.

Author	Year	Setting	Study Quality	Cognitive Variables	Relationship
An et al.	2019	Multicentre prospective, longitudinal	Moderate	Memory, attention, executive function, processing speed, and global cognition	TC: - (Executive Function, processing speed, global cognition) HDL-C: - (Global Cognition); + (Executive function, processing speed) LDL-C: - (Memory, attention, global cognition)
Cherbuin et al.	2009	Prospective, longitudinal	High	Global cognition	Lipid Lowering medication: - (Global cognition)
Chuang et al.	2023	Retrospective	Moderate	Global Cognition	TC: 0 (Global Cognition) HDL-C: 0 (Global Cognition) LDL-C: 0 (Global Cognition) TG: 0 (Global Cognition) Dyslipidemia: 0 (Global Cognition)
Henderson et al.	2003	Longitudinal	Moderate	Memory	TC, LDL-C: - (Memory)
Kaffashian et al.	2013	Prospective	High	Memory, executive function, attention, global cognition, inductive reasoning	0
Kivipelto et al.	2001	Prospective and cross-sectional analysis of population-based, longitudinal	High	Memory, attention, executive function, global cognition	TC: - (Global cognition)
Nunley et al.	2017	Prospective, observational	High	Memory, attention, executive function, global cognition, intelligence and psychomotor speed	Lipid-lowering Medication: - (Memory and psychomotor speed)
Power et al.	2017	Prospective	High	Memory, executive function, global cognition	TC, TG: - (Memory, executive function, Global cognition) HDL, LDL: - (Executive function)
Reynolds et al.	2011	Longitudinal, population-based	Moderate	Memory, global cognition, perceptual speed, verbal and spatial ability	TG: - (Spatial ability, perceptual speed, and general cognition – Females)
Szoeke et al.	2019	Longitudinal	High	Global Cognition	0
Tuligenga et al.	2014	Prospective, longitudinal	Moderate	Memory, executive function, and inductive reasoning	0
Wendell et al.	2014	Prospective	High	Memory, attention, executive function, global cognition, and visuospatial ability	TC: - (Memory); + (Memory, executive function, global cognition)
Yang et al.	2018	Prospective	Moderate	Psychomotor speed, attention, executive function, memory	Hypercholesterolemia: + (verbal learning and memory)

0, no association; -, negative association; +, positive association.

In summary, there was inconsistent reporting on the relationship between midlife cholesterol metrics and later-life cognitive function.

### Associations between cholesterol metrics at midlife and measures of cognition at midlife

Table 2 details mean pooled outcomes for all midlife cognitive measures used and associated cholesterol metrics. Of all included studies, most (n = 82, 79.6%) reported no relationship between cholesterol metrics at midlife and cognitive measures at midlife, including memory, attention, executive function, global cognition, inductive reasoning, intelligence, psychomotor speed, and visuospatial organisation. The majority of studies reported no significant relationship between the specific cholesterol measure and cognitive function, including, **TC** [*memory*: 85.1% (n = 40); *attention*: 94.1% (n = 17); *executive function*: 71.1% (n = 38); *global cognition*: 69.6% (n = 23)], **HDL-C** [*memory*: 89.5% (n = 34), *attention*: 94.4% (n = 17); *executive function*: 81.2% (n = 34); *global cognition*: 95.2% (n = 20)], **LDL-C** [*memory*: 78.3% (n = 18); *attention*: 84.6% (n = 11); *executive function*: 86.4% (n = 19); *global cognition*: 92.9% (n = 13)], **TG** [(*memory*: 88% (n = 22); *attention*: 92.3% (n = 12); *executive function*: 91.7% (n = 22); *global cognition*: 82.3% (n = 14)], **hypercholesterolemia** (*memory*: 69.2% (n = 9); *attention*: 100% (n = 5); *executive function*: 76.9% (n = 10); *global cognition*: 88.9% (n = 8), **lipid lowering medication** [*memory*: 91.7% (n = 11); *global cognition*: 83.3% (n = 5)], and **dyslipidemia** [*executive function*: 80% (n = 4); *global cognition*: 57% (n = 4)].

All remaining studies reported a negative relationship with midlife cognition across select cognitive domains across both the individual study cohorts [**TC** (n = 7), **HDL-C** (n = 2), **LDL-C** (n = 3), **TG** (n = 2), **hypercholesterolemia** (n = 2), **lipid lowering medication** (n = 1), and **dyslipidemia** (n = 3)], and the longitudinal national cohorts [**TC** (n = 7), **HDL-C** (n = 4), **LDL-C** (n = 4), **TG** (n = 2), **hypercholesterolemia** (n = 3), and **lipid lowering medication** (n = 1)].

In our analysis of the associations between cholesterol metrics and measures of cognition at midlife, we observed distinct patterns based on study quality. Moderate to high-quality studies predominantly reported no significant relationship (Moderate (n = 33); High (n = 35)), whereas low-quality studies more frequently reported negative associations (n = 14). This trend was consistent across various cholesterol metrics and cognitive domains. Moderate to high-quality studies generally reported no significant relationship between **TC** and cognition, while low-quality studies indicated negative associations, particularly in domains such as memory (low (n = 1), moderate (n = 5), high (n = 1)), attention (moderate (n = 5)), and executive function (low (n = 1), moderate (n = 7), high (n = 3)). Similar to TC, moderate to high-quality studies between **HDL-C** and cognition showed no significant relationship, whereas low-quality studies reported negative associations, especially in domains like memory (moderate (n = 2), high (n = 2), attention: high (n = 1)) and executive function (moderate (n = 2), high (n = 3)). Moderate to high-quality studies mostly reported no significant relationship between **LDL-C** and cognition, with some exceptions, while low-quality studies indicated negative associations, particularly in domains such as memory (low (n = 1), moderate (n = 3), high (n = 1)), attention (moderate (n = 1), high (n = 1)) and executive function (moderate (n = 1), high (n = 2)). In line with these findings, negative associations between **TG’s** and cognitive function were predominantly observed in low-quality studies, especially in domains like memory (low (n = 2), moderate (n = 1), high (n = 1)), executive function (low (n = 2), high (n = 1)), and global cognition (low (n = 1), moderate (n = 1)). Moreover, negative associations were more frequently reported in low-quality studies for **hypercholesterolemia**, **lipid-lowering medication**, and **dyslipidemia**, primarily in domains such as memory (low (n = 3), moderate (n = 1), high (n = 2)), executive function (low (n = 2), high (n = 2)), and global cognition (low (n = 1), moderate (n = 3), high (n = 2)). For further detailed numerical breakdown by study quality across all cognitive domains please refer to Supplemental Table B2.

## Discussion

This systematic review aimed to assess the relationship between midlife cholesterol and its sub-components with cognitive function in both mid- and later-life. Our analysis shows inconsistent reporting of any such association, and we conclude therefore that midlife circulating cholesterol cannot predict cognitive status at either mid- or later-life. While some individual cohort studies reported negative relationships (n = 3, 50%) between midlife cholesterol and cognitive domains in later life, the majority of longitudinal cohort studies (n = 3, 75%) found no significant link. Furthermore, at midlife, most studies (n = 81, 78.6%) reported no association between cholesterol metrics and cognitive function, regardless of the specific cognitive domain assessed, while a subset of studies even reported positive associations. Findings were inconsistent irrespective of study design or study quality. Overall, our review failed to identify a clear link between midlife cholesterol levels and cognitive function in mid- and later-life, contrasting with recent evidence that led to the inclusion of cholesterol as a modifiable risk factor for dementia by the Lancet Commission (Livingston et al., 2024). According to the Commission, this specific update from the 2020 report, that excluded cholesterol as a dementia risk factor, was due in part to publication of a systematic review and metanalysis (Wee et al., 2023) that assessed not only cognitive impairment, but dementia diagnosis. Here, we focus on cognitive performance across a range of specific domains at both midlife and older age, rather than dementia *per se*. As such, while our results may conflict with those of the Lancet commission, the emphases of each study differ.

Our findings add to the existing body of literature that has investigated the relationship between circulating cholesterol levels and cognitive function across different life stages. While our focus was primarily on middle-aged adults, the conclusions drawn from our review share similarities with previous systematic evidence targeting older populations, albeit with notable differences. Despite the emphasis on middle-aged adults in our analysis, our conclusions align with the findings of Peters et al. (2021) who, in their analysis of longitudinal studies of cardiovascular and metabolic risk factors of dementia, report inconsistent evidence supporting cholesterol as a reliable predictive marker of development of dementia. While Peters et al. (2021) included 8 studies of over 21,000 participants aged 60 years or over, our expanded analysis included 106 articles encompassing more than 200,000 participants. Our analysis of the published literature reveals that individual cohort studies (50%) were more likely to report adverse associations when compared with other study types, underscoring the need for high-quality systematic reviews to be conducted in order to develop a clear picture of the relationship between CV risk factors and brain health throughout the lifespan. The heterogeneity in study findings emphasises the importance of considering methodological factors that may influence the observed associations. Variability in study design, participant characteristics, cognitive assessment tools, number of included studies, and statistical analysis methods could contribute to the discrepant results reported across studies. For example, differences in sample size and participant age range may influence the statistical power to detect significant associations. Moreover, the choice of cognitive measures and the sensitivity of these measures to subtle changes in cognitive function can impact the validity of study findings. Standardizing assessment protocols and employing rigorous statistical techniques can help mitigate potential sources of bias and enhance the reliability of study outcomes.

Similar to our findings on TC, despite variations in individual study results, a negative relationship between midlife cholesterol metrics and later-life cognitive function was not observed. Notably, our review highlighted potential protective roles for midlife HDL-C and hypercholesterolemia in preserving cognitive function in later life. These findings align with previous evidence, suggesting a protective effect of elevated HDL-C levels on cognitive decline in later middle age and early later-life demonstrating a positive correlation with memory performance among older adults (Armstrong et al., 2019; Marcum et al., 2018; Mielke et al., 2008; Kinno et al., 2019). Recent systematic evidence further supports our observations, indicating that the increased risk of mild cognitive impairment associated with elevated TC at midlife is mitigated by 61% in individuals with elevated HDL-C during the same period (Wee et al., 2023). These findings suggest a functionally protective capacity of elevated HDL-C on cognition with advancing age.

The results of this review add to the existing understanding that midlife cholesterol may not have a significant short-term influence on brain health, reflected in our finding of no relationship between midlife cholesterol and midlife cognitive function. This held true regardless of the specific domain of cognitive function that was assessed, such as memory, executive function, or attention. Perhaps it is not surprising that the lack of a link between these measures at later life is observed when examined decades earlier. As such, based on our results and others, it is possible, if not probable that circulating cholesterol is at best a minor contributor to the profile of CV risk factors that may be of use in predicting risk of cognitive decline from midlife onwards.

Despite the insights gained from our systematic review, several limitations must be acknowledged. Significant heterogeneity across studies, inconsistent reporting of cognitive test data, and varying follow-up times limit cross-study comparisons and the ability to draw definitive conclusions. The lack of comparative groups and raw data within studies also hindered the possibility of conducting a meta-analysis. Moreover, our review did not consider the known contribution of specific genes, such as the APOe4 gene, to the relationship between cholesterol and cognition. Lastly, the variance in ages at which blood samples and cognitive testing were conducted warrants caution in interpreting the findings.

## Conclusion

While epidemiological and pathological investigations have suggested that midlife modifiable CV risk factors such as high cholesterol may lead to cognitive decline, reports are inconsistent, and the literature remains heterogeneous. The present review summarises the current evidence and indicates that, despite these inconsistencies between individual studies, no relationship exists between cholesterol measured at midlife and multiple cognitive domains, either at either mid- or later-life. Strategies to prevent cognitive decline in later life should therefore focus more strongly on modifiable risk factors shown to have greater negative impacts on cognitive function throughout the lifespan, such as hypertension and diabetes.

## Data Availability

The original contributions presented in the study are included in the article/Supplementary Material, further inquiries can be directed to the corresponding author.
