# Supplementary material for: The influence of circulating cholesterol and its components in middle-aged adults on cognitive function at mid- and later-life; a systematic review
Source: Front Aging. 2025 Mar 19;6:1430382. doi: 10.3389/fragi.2025.1430382 (PMC11962029; doi:10.3389/fragi.2025.1430382)
Supplement: Supplementary file 1 [file DataSheet1.docx]

**List of Supplemental Digital Content**

**Appendix A.1 Search Strategy**

**Supplemental Table B.1.** Summary of all studies with negative relationships between cholesterol, associated metrics, and cognitive measures at midlife.

**Supplemental Table B.2.** Summary of studies with no relationship between cholesterol, associated metrics, and cognitive measures at midlife.

**Supplemental Table B.3.** Summary of negative relationships between cholesterol metrics and cognition at midlife by study design and quality.

**Supplemental Table B.4.** Critical appraisal of included studies using the AXIS tool.

**Supplemental Table B.5.** List of reference studies used for longitudinal cohort studies.

**Supplemental Table B.6.** Cholesterol metrics, hypercholesterolemia status, and cognitive measures across all included studies.

**Supplemental Table B.7.** List of Cognitive tests used across all cognitive domains.

# **Search Strategy**

Searches run 23/02/21

Number of results from all searches = 7326

After deduplication = 5313

Searches run 19/04/22

Number of results from all searches=3670

After deduplication= 1162

Searches run 19/12/23

Number of results from all searches=4459

After deduplication= 749

### **EMBASE**

('cognitive defect'/exp OR 'cognition'/exp) AND 'risk factor'/exp

((cognition OR cognitive) NEAR/2 health*):ti,ab

((cognition OR cognitive) NEAR/2 function*):ti,ab

((Cognitive OR cognition OR memory) NEAR/2 (impair* OR disorder* OR defect* OR decline?)):ti,ab

((dementia OR Alzheimer?) NEAR/3 risk*):ti,ab

#1 OR #2 OR #3 OR #4 OR #5

'diabetes mellitus'/exp

(diabetes OR 'diabetes mellitus' OR diabetic*):ti,ab

'cholesterol'/exp OR 'total cholesterol level'/exp

(cholesterol OR cholesterine OR cholesterin):ti,ab

#7 OR #8 OR #9 OR #10

'middle aged'/exp

('middle age?' OR middleaged or midlife OR ‘mid life’ OR ‘mid age’ OR ‘aged 40–65’ OR ‘middle years’):ti,ab

#12 OR #13

'cohort analysis'/exp

'longitudinal study'/exp

'prospective study'/exp

'follow up'/exp

Cohort?:ti,ab

'case control study'/exp

(case* and control*):ti,ab

#15 OR #16 OR #17 OR #18 OR #19 OR #20 OR #21

#6 AND #11 AND #14 AND #22 AND [english]/lim

('conference abstract' OR 'conference report' OR letter OR editorial):it

#23 NOT #24

### **Medline**

(exp Cognition Disorders/ OR exp cognition/) AND Risk Factors/

((cognition OR cognitive) adj2 health*).ti,ab.

((cognition OR cognitive) adj2 function*).ti,ab.

((Cognitive OR cognition OR memory) adj2 (impair* OR disorder* OR defect* OR decline?)).ti,ab.

((dementia OR Alzheimer?) adj3 risk*).ti,ab.

or/1-5

exp Diabetes Mellitus/

(diabetes OR diabetes mellitus OR diabetic*).ti,ab.

exp Cholesterol/ OR Hypercholesterolemia/

(cholesterol OR cholesterine OR cholesterin).ti,ab.

or/7-10

Middle Aged/

(middle age? OR middleaged or midlife OR mid life OR mid age OR middle years).ti,ab.

or/12-13

exp cohort studies/

cohort$.tw.

controlled clinical trial.pt.

epidemiologic methods/

limit 18 to yr=1971-1988

or/15-17,19

6 AND 11 AND 14 AND 20

## **Web of Science 460 Articles**

TS =(((cognition OR cognitive) NEAR/2 health*) OR ((cognition OR cognitive) NEAR/2 function*) OR ((Cognitive OR cognition OR memory) NEAR/2 (impair* OR disorder* OR defect* OR decline?)) OR ((dementia OR Alzheimer?) NEAR/3 risk*))

TS =(diabetes OR “diabetes mellitus” OR diabetic* OR cholesterol OR cholesterine OR cholesterin)

TS =(“middle age?” OR middleaged or midlife OR “mid life” OR “mid age” OR “aged 40–65” OR “middle years”)

TS =(“cohort analysis” OR “longitudinal study” OR “prospective study” OR “follow up” OR Cohort* OR “case control stud*” OR (case* and control*))

#1 AND #2 AND #3 AND #4

## **CINAHL 1434 Results**

(MH "Cognition In Old Age" OR MH "Cognition Disorders In Old Age" ) AND (MH "Risk Factors")

TI ((cognition OR cognitive) N2 health*) OR AB ((cognition OR cognitive) N2 health*)

TI ((cognition OR cognitive) N2 function*) OR AB ((cognition OR cognitive) N2 function*)

TI ((Cognitive OR cognition OR memory) N2 (impair* OR disorder* OR defect* OR decline*)) OR AB ((Cognitive OR cognition OR memory) N2 (impair* OR disorder* OR defect* OR decline*))

TI ((dementia OR Alzheimer*) N3 risk*) OR AB ((dementia OR Alzheimer*) N3 risk*)

S1 OR S2 OR S3 OR S4 OR S5

(MH "Diabetes Mellitus+")

TI (diabetes OR “diabetes mellitus” OR diabetic*) OR AB (diabetes OR “diabetes mellitus” OR diabetic*)

(MH "Cholesterol+") OR (MH "Cholesterol, Dietary") OR (MH "Embolism, Cholesterol")

TI (cholesterol OR cholesterine OR cholesterin) OR AB (cholesterol OR cholesterine OR cholesterin)

S7 OR S8 OR S9 OR S10

(MH "Middle Age")

TI (“middle age*” OR middleaged or midlife OR “mid life” OR “mid age” OR “aged 40–65” OR “middle years”) OR AB (“middle age*” OR middleaged or midlife OR “mid life” OR “mid age” OR “aged 40–65” OR “middle years”)

S12 OR S13

(MH "Prospective Studies+")

(MH "Case Control Studies+")

(TI (case or cases) n5 TI (control or controls)) OR (AB (case or cases) n5 AB (control or controls))

(TI (case or cases) n3 TI (matched)) OR (AB (case or cases) n3 AB (matched)) OR TI (control group*)

TI (cohort N3 (stud* OR analysis)) OR AB (cohort N3 (stud* OR analysis))

TI (observational N3 (stud*)) OR AB (observational N3 (stud*))

TI (“longitudinal stud*”) OR AB (“longitudinal stud*”)

S15 OR S16 OR S17 OR S18 OR S19 OR S20 OR S21

S6 AND S11 AND S14 AND S22

***Supplemental Table B.1.*** Summary of all studies with negative relationships between cholesterol, associated metrics, and cognitive measures at midlife.

| Author | Year | Study Design | Setting | Study Quality | Participants | Cognitive Variables | Relationship |  |
| --- | --- | --- | --- | --- | --- | --- | --- | --- |
| Aliberti et al | 2020 | Cross sectional | ELSA, Brazil | High | n= 5,275 Age= 56.3 | Memory, executive function, temporal orientation, global cognition | Dyslipidaemia: - (Global cognition, semantic verbal fluency) |  |
| An et al. | 2019 | Multicentre prospective, longitudinal | EMCOA; China | Moderate | n =2514 (males = 1156, females = 1358)  Age = 59 | Memory, attention, executive function, processing speed, and global cognition | TC: - (Executive Function, processing speed, global cognition; mid-late life)  HDL-C: - (Global Cognition); mid-late life)  LDL-C: - (Memory, attention, global cognition; mid-late life) |  |
| Backestrom et al. | 2015 | Retrospective, cross sectional | Betula Prospective Cohort, Sweden | Moderate | n= 291 (males= 127, females= 164) Age= 50.7 (8) [males= 50.3 (8), females= 51 (8.1)] | Memory | TC: - (Memory) |  |
| Bressler et al. | 2013 | Prospective | ARIC, USA | Low | White: n= 8,364 (males= 3,859, females= 4,505); African American: n= 2,083 (males= 716, females= 1,367) Age: White= 57 (5.6); African American= 55.8 (5.7) | Memory, executive function | LDL: - (Memory) |  |
| Cherbuin et al. | 2009 | Prospective, longitudinal | PATH, Australia | High | Wave 2  n= 2,018 (males= 1022, females= 996)  Age= 62.5 (1.5) | Global cognition | Lipid/Cholesterol Lowering Medication: - (Global cognition) |  |
| Christman et al. | 2011 | Prospective | ARIC, USA | Moderate | n= 8,958 (males= 3,943, females= 5,015)  Age= 56.5 (5.6) | Memory and executive function | TC: - (Executive function) |  |
| Dearborn et al. | 2014 | Cross sectional, longitudinal | ARIC, USA | High | n= 10,495  Age: Males = 54.7 (5.6), Females = 54.8 (5.5) | Memory, executive function | HDL, TG: - (Memory and executive function) |  |
| Del Vecchio et al. | 2023 | Retrospective, Case Control | Rome, Italy | Moderate | n = 112 (males = 81, females = 31)  Control Group: n = 31 (males = 20, females = 11)  Study Group: n = 81 (males = 61, females = 41) | Global Cognition | Hypercholesterolemia: - (Global Cognition) |  |
| Elkins et al. | 2005 | Prospective | ARIC, USA | High | n= 12,096 (males= 12,039, females= 57) Age= 57 (5.7) | Memory, executive function | HDL, LDL: - (Executive function) |  |
| Ferguson et al. | 2018 | Cross sectional | CARDIA, USA | Moderate | n= 634 (males= 305, females=329)  Age= 50.4 (3.5) | Memory, executive function | TC: - (Executive function) |  |
| Gerber et al. | 2021 | Multicentre population-based cohort | CARDIA; USA | Low | Total: n = 2809  No NAFLD >51 HU: n=2136  Mild NAFLD >40–51 HU: n=392  Severe NAFLD ≤40 HU: n=281  Age:  Total = 50.1 (3.6)  No NAFLD >51 HU = 50 (3.7)  Mild NAFLD >40–51 HU = 50.3 (3.6)  Severe NAFLD ≤40 HU = 50.5 (3.6) | Memory, executive function, psychomotor speed, and global cognition | Hypercholesterolemia & Hypertriglycemia: - (Memory, executive function, and global cognition) |  |
| Gonzalez et al. | 2018 | Prospective, epidemiologic | ARIC, USA | Moderate | n= 13,720 (males= 5,873, females= 7,397) Age= 54.1 (5.7) | Memory, executive function, global cognition | TC: - (Global cognition) |  |
| Gourley et al. | 2020 | Cross sectional | Texas, USA | Moderate | n= 132 (males= 59, females= 73) Age= 49 (6) | Memory, executive function, intelligence, attention | TC, HDL: - (Memory, executive function) |  |
| Henderson et al. | 2003 | Longitudinal | MWMHP; Australia | Moderate | n = 438 (All female)  age = 56.7 (2.5) | Memory | LDL-C: - (Memory; mid-mid, mid-late)  TC: - (Memory; Mid-late) |  |
| Houle et al. | 2019 | Cross sectional, longitudinal | HAALSI, South Africa | Moderate | n= 2,059 (males= 2,345, females= 2,714) Age= 40- 59 | Memory, executive function, attention, global cognition, temporal orientation | Dyslipidaemia: - (Global cognition) |  |
| Kaffashian et al. | 2013 | Prospective | Whitehall II, UK | High | n= 4,374 (males= 3,162, females= 1,212)  Age= 55.2 (5.1) | Memory, executive function, attention, global cognition, inductive reasoning | TC: - (Executive function, global cognition, inductive reasoning) |  |
| Kivipelto et al. | 2001 | Prospective and cross-sectional analysis of population-based, longitudinal | North Karelia Project and the FINMONICA study; Finland | High | Total: N = 1449; MCI: N = 82, Without MCI: N = 1270  Age:  Midlife: MCI = 51.7 (5.8); Without MCI = 50.1 (6.0)  Late life: MCI = 72.8 (4.1), Without MCI = 71.0 (3.9) | Memory, attention, executive function, global cognition | TC: - (Global cognition; mid-late) |  |
| Knopman et al. | 2001 | Longitudinal | ARIC, USA | Low | n= 10,882 (males= 6,978, females= 3,904) Age= 56.8 (5.7) | Memory, executive function | Hypercholesterolemia: - (Memory, executive function) |  |
| Knopman et al. | 2018 | Longitudinal | ARIC, USA | Low | n= 10,882 (males= 8,723, females= 7,137) Age= 51.4 (4.9) | Memory, executive function, global cognition | TC: - (Global cognition) |  |
| Kovacs et al. | 2014 | Cross sectional | Hungary | Moderate | *Hypertensive*= 72; *controls*= 85  Age: *Hypertensive*= 43.6; *controls*= 43.6 | Memory, executive function, attention, psychomotor speed, visuospatial organisation | LDL: - (Memory, attention, executive function, psychomotor speed) |  |
| Kumari et al., | 2005 | Longitudinal | Whitehall II, UK | Moderate | NGT: males= 3,407, females= 1,334; IGT: males= 405, females= 192; Diabetes: males= 208, females= 101  Age: NGT: males= 55.1, females= 55.7; IGT: males= 58.2, females= 57.8; Diabetes: males= 57.9, females= 58.9 | Memory, inductive reasoning, executive function | HDL: - (Inductive reasoning, executive function, and memory) |  |
| Lutski et al. | 2019 | Longitudinal | BIP, Israel | High | n= 337  Age= 56.6 (6.4) | Memory, executive function, attention, global cognition, visuospatial organisation | HDL, LDL, TG: - (Memory, attention, global cognition, visuospatial organisation) |  |
| Mahinrad et al. | 2020 | Longitudinal | CARDIA, USA | Moderate | n= 191 (males= 104, females= 87)  Age= 56 (4) | Memory, executive function, attention | TC: - (Memory, executive function, global cognition) |  |
| Masi et al. | 2018 | Prospective | MRC NSHD, UK | Moderate | n= 1,249 (males= 578, females= 671)  Age= 60–64 | Memory, attention, executive function | TC: - (Memory) |  |
| Mefford et al. | 2021 | Multicentre longitudinal, prospective | CARDIA study, USA | Moderate | N = 3328  Time-averaged LDL-C levels over follow-up, mg/dL: <100 (n = 519) 100–129 (n = 1,094) 130–159 (n = 961) ≥160 (n = 754)  Age:  Time-averaged LDL-C levels over follow-up, mg/dL: <100 = 46.9 (3.2); 100–129 = 49.2 (3.5); 130–159 = 51.1 (3.1); ≥160 = 52.6 (2.5) | Memory, attention, and executive function | LDL-C: - (Memory) |  |
| Moore et al. | 2014 | Longitudinal | VETSA, Thailand | High | n= 651 (all males)  Age= 55.3 (3.1) | Executive function, memory, visuospatial organisation, intelligence | Hypercholesterolemia: - (Memory, and executive function) |  |
| Muhkerjee et al. | 2016 | Prospective | MACS, USA | Moderate | n = HIV-: 516  age = 51.5 | Memory, attention, executive function, processing speed, perceptual speed, | TC: - (Memory and attention |  |
| Nunley et al. | 2017 | Prospective, observational | Pittsburgh Epidemiology of Diabetes Complications Study; USA | High | N = 108  Age = 49.52 (7.04) | Memory, attention, executive function, global cognition, intelligence and psychomotor speed | Lipid-lowering Medication: - (Memory and psychomotor speed; mid-late) |  |
| Panigrahi et al. | 2021 | Cross sectional | New Delhi, India | Moderate | n= 80 (males= 31, females= 49)  Age= 51.7 (7.2) | Global cognition | Dyslipidaemia: - (Global cognition) |  |
| Power et al., | 2017 | Prospective | ARIC, USA | High | n= 15,792 Age= 57.5 (5.7) | Memory, executive function, global cognition | TC, TG: - (Memory, executive function, Global cognition)  HDL, LDL: - (Executive function) |  |
| Reynolds et al. | 2011 | Longitudinal, population-based | SASTA; Sweden | Moderate | n = 819 (males = 335, females = 484) | Memory, global cognition, perceptual speed, verbal and spatial ability | TC: - (Perceptual speed – Males; mid-midlife)  TG: - (Global cognition – Males; mid-midlife; Spatial ability, perceptual speed, and general cognition – Females; mid-later life) |  |
| Singh-Manoux et al. | 2009 | Cross-sectional, longitudinal | Whitehall II, UK | Moderate | n= 5,292 (males= 3,810, females= 1,481)  Age: CHD= 59.4 (5.5); No CHD= 55.2 (5.9) | Memory, executive function, inductive reasoning | TC: - (Global cognition, Executive function, inductive reasoning) |  |
| Solomon et al. | 2009 | Prospective | CAIDE, Finland | Low | Low TC: n= 123 (males= 40, females= 83);  Intermediate TC: n= 365 (males= 151, females= 216;  High TC: n= 894 (males= 332, females= 566)  Age: Low TC= 49.1; Intermediate TC= 48.8; High TC= 50.4 | Memory, executive function, global cognition, psychomotor speed | TC: - (Memory and Executive function)  Lipid/Cholesterol Lowering Medication: - (Memory and psychomotor speed) |  |
| Wendell et al. | 2014 | Prospective | BLSA, USA | High | n = 1601 (male = 813, female = 788)  Age = 54.4 (16.4) | Memory, attention, executive function, global cognition, and visuospatial ability | TC: - (Executive function, global cognition, verbal learning, and language; midlife-midlife)  TC: - (Memory; mid-late life) |  |
| Yaffe et al. | 2014 | Longitudinal, prospective | CARDIA, USA | Moderate | Year 25 Examination  n= 3,381 (males= 1,475, females = 1,906)  Age= 50.2 (3.6) | Memory, executive function, psychomotor speed | TC: - (Executive function) |  |
| Yang et al. | 2018 | Prospective | MACS, USA | Moderate | n= 900 (all males) | Psychomotor speed, attention, executive function, memory | Hypercholesterolemia: - (Memory) |  |

0, no association; -, negative association; +, positive association

**Abbreviations:** *ACE*, Akershus Cardiac Examination; *APAC,* Asymptomatic Polyvascular Abnormalities Community; *ARIC, Atherosclerosis Risk in Communities*; *ASCEND*, A Study of Cardiovascular Events in Diabetes; *Barcelona-AsIA*, Asymptomatic Intracranial Atherosclerosis; *BHS*, Bogalusa Heart Study; *BIP,* Bezafibrate Infarction Prevention; *BP,* blood pressure; *BLSA*, Baltimore Longitudinal Study of Aging; *CARDIA,* Coronary Artery Risk Development in Young Adults; *CHARLS*, China Health and Retirement Longitudinal Study; *DBP*, diastolic blood pressure; *ELSA*, English Longitudinal Study of Ageing; ELSA, Brazilian Longitudinal Study of Adult Health; EMCOA, *Effects and Mechanism Investigation of Cholesterol and Oxysterol on Alzheimer’s disease*; *FINMONICA,* Finnish Multinational Monitoring of Trends and Determinants in Cardiovascular Disease*; HAALSI*, Health and Aging in Africa; *HANDLS, healthy Aging in Neighborhoods of Diversity Across the Life Span*; *HHP*, Honolulu Heart Program; *KALS,* Kaohsiung Atherosclerosis Longitudinal Study ; *KEEPSCog*, Kronos Early Estrogen Prevention cognitive; *KIHD,* Kuopio Ischaemic Heart Disease Risk Factor Study; *MACS,* Multicentre AIDS Cohort Study; *MADT*, Middle-Aged Danish Twins; *MDCS,* Malmö Diet and Cancer Study; *MORGEN*, Monitoring Project on Cardiovascular Disease Risk Factors; *MRC,* Medical Research Council; MWMHP, Melbourne Women’s Midlife Health Project; NSHD*,* National Survey of Health and Development; *PATH,* Population Assessment of Tobacco and Health*; PURE,* prospective Urban and Rural Epidemiological; *RECALL*, Risk Factors, Evaluation of Coronary Calcium and Lifestyle; SASTA, Swedish Adoption/Twin Study of Aging; SBP, systolic blood pressure; *Swan,* Study of Women’s Health Across the Nation; TILDA, The Irish Longitudinal Study on Ageing; *VETSA,* Vietnam Era Twin Study of Aging and; *WHAP,* Women’s Health Aging Project

***Supplemental Table B.2.*** Summary of studies with no relationship between cholesterol, associated metrics, and cognitive measures at midlife.

| Author | Year | Study Design | Setting | Study Quality | Participants | Cognitive Variables | Relationship |  |
| --- | --- | --- | --- | --- | --- | --- | --- | --- |
| Albanese et al. | 2012 | Longitudinal | UK | Moderate | n= 2,083 (males= 1,013, females= 1,070)  Age= 43-53 | Memory, attention, executive function, global cognition | TC |  |
| Alves de Moraes | 2002 | Longitudinal | ARIC, USA | Low | n= 8,058  Age= 56.7 (5.6) | Memory, executive function | TC, HDL, LDL, TG |  |
| An et al. | 2019 | Multicentre prospective, longitudinal | EMCOA; China | Moderate | n =2514 (males = 1156, females = 1358)  Age = 59 | Memory, attention, executive function, processing speed, and global cognition | 0 Lipid/Cholesterol lowering medication, TG’s |  |
| Babaei et al. | 2013 | RCT | Iran | Low | n= 52 (28 patients vs. 24 controls) Age= 57.1 (5.9) | Memory | TC, HDL, LDL |  |
| Boots et al. | 2015 | Cross sectional | WRAP, USA | High | n= 315 (males= 102, females= 213) Age= 58.6 (6.3) | Memory, executive function, visuospatial organisation, global cognition | TC, HDL |  |
| Bressler et al. | 2013 | Prospective | ARIC, USA | Low | White: n= 8,364 (males= 3,859, females= 4,505); African American: n= 2,083 (males= 716, females= 1,367) Age: White= 57 (5.6); African American= 55.8 (5.7) | Memory, executive function | TC, HDL, TG, Hypercholesterolemia |  |
| Britton et al. | 2004 | Cross sectional. Longitudinal | Whitehall II Study; UK | Low | Males = 4272, Females = 1761  Age = Males: 55.5 (6), Females = 56 (6.04) | Memory, executive function, inductive reasoning | TC |  |
| Brunner et al. | 2017 | Cross sectional, longitudinal | Whitehall II, UK | Low | Visit 5 and 6  n= 175  Age= (55-64 | Global cognition | TC, HDL |  |
| Carmichael et al. | 2019 | Observational, longitudinal | BHS; USA | Low | Total: n = 50 (male = 26, female = 14)  Age = 48.8 (8.7) | Memory, executive function | TC |  |
| Cerhan et al. | 1998 | Longitudinal | ARIC; USA | Moderate | Total: n = 13913  Age = 45-64 | Memory, executive function, psychomotor speed | TC |  |
| Chen et al. | 2015 | Cross sectional, longitudinal | WHAP Study; Australia | High | n= 247 Age= 50.1 (2.6) | Executive function, psychomotor speed, memory | TC, HDL, LDL |  |
| Chosy et al. | 2019 | Longitudinal | Honolulu Heart Program (HHP); USA | Moderate | Total = 3,582 participants (SCI Absent)  Age = 52.3 (4.4) | Memory, attention, executive function, global cognition, inductive reasoning | TC |  |
| Christman et al. | 2011 | Prospective | ARIC, USA | Moderate | n= 8,958 (males= 3,943, females= 5,015)  Age= 56.5 (5.6) | Memory, executive function | HDL, LDL, TG |  |
| Cohen-Manheim et al. | 2016 | Cross sectional | Jerusalem, Israel | High | n= 507 (males= 343, females= 164) Age= 49.9 (0.8) | Memory, executive function, attention | TC, HDL, LDL, TG |  |
| Creavin et al. | 2012 | Prospective, longitudinal | Caerphilly, Wales | Moderate | Phase 1  n= 2,512  Age= 52.8 | Attention, global cognition, inductive reasoning | HDL, TG |  |
| Cui et al. | 2016 | Case-control | Guangzhou, China | Moderate | *Hypertensive*: n= 278; *Controls*= 155 Age: *Hypertensive*= 54.2 (4.2); *Controls*= 55.8 (5.5) | Intelligence, global cognition | TC, HDL, LDL, TG |  |
| de Menezes et al. | 2021 | Longitudinal | ELSA, Brazil | High | n= 7,063  Age= 58.9 (5.9) | Memory, executive function, global cognition | TC,  Lipid/Cholesterol Lowering Medication |  |
| Dearborn et al. | 2014 | Cross sectional, longitudinal | ARIC, USA | High | n= 10,495  Age: Males = 54.7 (5.6), Females = 54.8 (5.5) | Memory, executive function | Lipid/Cholesterol Lowering Medication |  |
| Dearborn-Tomazos et al. | 2019 | Longitudinal observational | ARIC, USA | Low | n= 13,588 (males= 3,000, females= 7,588) Age= 54.6 (5.7) | Memory, executive function, global cognition | TC |  |
| Debette et al. | 2011 | Prospective | Framingham, USA | Moderate | n= 1,352 (males= 6,634, females= 718) Age= 54 (9) | Memory, executive function | Hypercholesterolemia |  |
| Derby et al. | 2021 | Longitudinal | SWAN, USA | Moderate | N = 1139  Age = 53.4 (2.6) | Memory and executive function | TG's, HDL-C, Hyperlipidemia |  |
| Elbaz et al. | 2014 | Longitudinal | Whitehall II, UK | High | n= 4,699 (males= 3,324, females= 1,375) Age= 48.6 (5.8) | Inductive reasoning | TC, HDL |  |
| Fava et al. | 2013 | Prospective longitudinal | Italy | Low | Total: n = 96 (Group A = 48, Group B = 48)  Age: Group A = 53 (7), Group B = 54.6 (8.1) | Memory, executive function, global cognition | TG’s |  |
| Fuh et al. | 2007 | Matched, case–control study from a population-based cohort | KIWI; Kinmen, Taiwan | Low | Normal (N = 144) Impaired glucose tolerance (N = 68) Diabetes mellitus (N = 72)  Age:  Normal = 47.9 (4.3) Impaired glucose tolerance = 46.8 (4.1) Diabetes mellitus = 47.9 (4.3) | Memory, attention, and executive function | TC, TG’s |  |
| Giugliano et al. | 2018 | RCT | Pozzilli, Italy | Low | *Active* *Treatment*: n= 18; *Control*: n=18  Age: *Active* *Treatment*= 58.2 (8); *Control*= 57.9 (6.7) | Executive function, global cognition | TC, HDL, LDL, TG |  |
| Gonzalez et al. | 2018 | Prospective, epidemiologic | ARIC, USA | Moderate | n= 13,720 (males= 5,873, females= 7,397) Age= 54.1 (5.7) | Memory, executive function, global cognition | Lipid/Cholesterol Lowering Medication |  |
| Gottesman et al. | 2017 | Prospective | ARIC, USA | High | n= 15,744 (males= 7,054, females= 8,690) Age= 54.2 (5.8) | Memory, executive function, global cognition | TC |  |
| Hajjar et al. | 2018 | Observational | USA | Low | n= 511 (males = 163, females = 348)  Age= 49.1 (0.5) | Memory, attention, executive function, global cognition, visuospatial ability | Hypercholesterolemia |  |
| Hakamada-Taguchi et al. | 2002 | Observational | Tokyo, Japan | Low | n= 26 (all females)  Age = 57.7 (1.1) | Memory, attention, executive function, intelligence | TC |  |
| Haley et al. | 2010 | Cross sectional | USA | Moderate | n= 38 Age= 50 (6.4) | Global cognition, intelligence, memory, attention, executive function, psychomotor speed | HDL, LDL, TG |  |
| Henderson et al. | 2003 | Longitudinal | MWMHP; Australia | Moderate | n = 438 (All female)  age = 56.7 (2.5) | Memory | TC, HDL-C, TG's, LDL-C, Lipid/Cholesterol lowering medication |  |
| Henriksen et al. | 2017 | Longitudinal, cohort study | The Metropolit Danish Male Birth Cohort, Denmark | High | Total: n = 189  Age = 58 (0.7) | Memory, attention, executive function, global cognition, psychomotor speed | TC, HDL, LDL |  |
| Hossain et al. | 2020 | Cross sectional, longitudinal | HANDLS, USA | High | n= 128 (males= 102, females= 126)  Age: males= 57.1 (0.5), females= 56 (0.8) | Memory, attention  executive function, global cognition | Dyslipidaemia |  |
| Ihle-Hansen et al. | 2019 | Prospective | ACE, Norway | High | n= 3,413 (males= 1,774, females= 1,639)  Age= 63.9 (0.7) [males= 63.9 (0.7), females= 63.9 (0.6)] | Global cognition | Lipid/Cholesterol lowering medication,  Hypercholesterolemia |  |
| John et al. | 2021 | Longitudinal cohort | NCDS; UK | High | N = 3730  Age = 44 | Memory, and executive function | TC, HDL-C, TG’s |  |
| Kaffashian et al. | 2013 | Prospective | Whitehall II, UK | High | n= 4,374 (males= 3,162, females= 1,212)  Age= 55.2 (5.1) | Memory, executive function, attention, global cognition, inductive reasoning | HDL |  |
| Kaffashian et al. | 2011 | Prospective | Whitehall II, UK | High | n= 4,827 (males= 3,486, females= 1,341) Age: males= 55.1 (5.9), females= 55.3 (5.9) | Memory, executive function, attention, global cognition, inductive reasoning | TC, HDL |  |
| Kalmijn et al. | 2002 | Prospective | MORGEN, The Netherlands | Moderate | n= 1,927 (males= 905, females= 989)  Age: males= 56.6 (7.1), females= 56.2 (7.1) | memory, executive function, global cognition, psychomotor speed | TC |  |
| Knopman et al. | 2009 | Longitudinal | ARIC Study; USA | Moderate | n= 1,130 (males= 429, females= 701) Age= 59 (4.3) | Memory, executive function | HDL, LDL, TG |  |
| Kovacs et al. | 2014 | Cross sectional | Hungary | Moderate | *Hypertensive*= 72; *controls*= 85  Age: *Hypertensive*= 43.6; *controls*= 43.6 | Memory, executive function, attention, psychomotor speed, visuospatial organisation | TC, HDL, TG,  Hypercholesterolemia |  |
| Kumar et al. | 2008 | Cross-sectional study | PATH Through Life Project; Australia | Moderate | Diabetics: N = 39; Non-diabetics: N = 428  Age:  Diabetics = 62.62 (1.16)  Non-diabetics = 62.55 (1.48) | Memory, attention, global cognition, and psychomotor speed | TC |  |
| Kumar et al., | 2020 | Longitudinal | ASCEND, UK | Low | n= 80 Age= 59 | Global cognition, attention,  executive function, memory | Hypercholesterolemia |  |
| Lin et al. | 2020 | Longitudinal | KALS, Taiwan | High | n= 528 Age= 53.9 (8.4) | Global cognition, memory, executive function, visuospatial orientation, attention | Hypercholesterolemia |  |
| Liu et al. | 2022 | Prospective | Neck-Shoulder and Lumbocrural Pain Hospital and the Affiliated Hospital of Shandong University of TCM; China | Moderate | Overall: n = 156; Controls = 64, SCI = 92  Age: Controls = 57.1 (6.3); SCI = 57.6 (6.7) | General Cognition | HDL-C, LDL-C |  |
| Lopez-Oloriz et al. | 2014 | Population-based | AsIA, Spain | Low | n= 95 Age= 59.9 (3.3) | Executive function, psychomotor speed, global cognition | Dyslipidaemia |  |
| Masi et al. | 2018 | Prospective | MRC NSHD, UK | Moderate | n= 1,249 (males= 578, females= 671)  Age= 60–64 | Memory, attention, executive function | HDL, LDL, TG |  |
| Mefford et al. | 2021 | Multicentre longitudinal, prospective | CARDIA study, USA | Moderate | N = 3328  Time-averaged LDL-C levels over follow-up, mg/dL: <100 (n = 519) 100–129 (n = 1,094) 130–159 (n = 961) ≥160 (n = 754)  Age:  Time-averaged LDL-C levels over follow-up, mg/dL: <100 = 46.9 (3.2); 100–129 = 49.2 (3.5); 130–159 = 51.1 (3.1); ≥160 = 52.6 (2.5) | Memory, attention, and executive function | TC, HDL-C, Lipid/cholesterol lowering medication |  |
| Muhkerjee et al. | 2016 | Prospective | MACS, USA | Moderate | n = HIV-: 516  Age = 51.5 | Memory, attention, executive function, processing speed, perceptual speed, | Lipid/Cholesterol lowering medication |  |
| Muhkerjee et al. | 2022 | Cross-sectional | Kolkata; India | Moderate | Total: n = 72 (diabetic retinopathy = 36, W/O diabetic retinopathy = 36)  Age:  Diabetic retinopathy = 55.2 (5.9)  W/O Diabetic retinopathy = 54.3 (6.8) | Memory, attention, executive function, global cognition | TC |  |
| Nunley et al. | 2017 | Prospective, observational | Pittsburgh Epidemiology of Diabetes Complications Study; USA | High | N = 108  Age = 49.52 (7.04) | Memory, attention, executive function, global cognition, intelligence and psychomotor speed | TC, HDL-C, LDL-C |  |
| Palacios-Mendoza et al. | 2018 | Cross sectional | Guayaquil, Ecuador | High | Diabetes: n= 142 (males= 65, females= 76); No Diabetes: n= 167 (males= 116, females= 50) Age: Diabetes= 59.9 (4.2); No Diabetes= 59.9 (3.8) | Memory, executive function, intelligence, attention | TC, HDL, LDL, TG, Lipid/Cholesterol lowering medication |  |
| Pearson et al. | 2017 | Prospective, longitudinal | CHALICE; New Zealand | High | Total: n = 404 (male = 189, female = 215)  Age: 50-54 | Global cognition | HDL-C, TG’s |  |
| Pokharel et al., | 2019 | Prospective | ARIC, USA | Moderate | n= 18,222 | Memory, executive function | TC, HDL |  |
| Rask et al. | 2016 | Longitudinal | Metropolitan 1953 Danish male birth cohort, Denmark | High | Total: n = 190 (Positive cognitive change = 93, Negative cognitive change = 97)  Age = 56 | Global cognition | TC |  |
| Ravona-springer et al. | 2020 | Prospective longitudinal | Israel Registry for Alzheimer Prevention (IRAP) study; Israel | Moderate | Total: n = 483; FH+ = 379, FH- = 104  Age: FH+ = 54.55 (6.76), FH- = 56.42 (6.19) | Memory, executive function, and global cognition | TC, HDL-C, LDL-C, TG, Hypercholesterolemia |  |
| Rawlings et al., | 2014 | Prospective | ARIC, USA | Moderate | n= 13,351  Age= 48-67 | Memory, executive function, global cognition | TC, HDL, TG |  |
| Razavi et al. | 2020 | Epidemiological | Bogalusa Heart, USA | High | n= 960  Age= 48.4 (5.1) | Memory, attention, executive function, processing speed | HDL, LDL, TG |  |
| Reis et al. | 2013 | Cross-sectional | CARDIA study; USA | Moderate | Total: n = 2510; Coronary artery calcified plaque: Present = 686, Absent = 1824; Abdominal aortic calcified plaque: Present = 1297, Absent = 1213  Age:  Coronary artery calcified plaque: Present = 51.1 (3.3), Absent = 49.6 (3.7); Abdominal aortic calcified plaque: Present = 50.6 (3.6), Absent = 49.5 (3.7) | Memory, attention, and executive function | HDL-C, LDL-C, TG, Dyslipidemia |  |
| Reynolds et al. | 2011 | Longitudinal, population-based | SASTA; Sweden | Moderate | n = 819 (males = 335, females = 484) | Memory, global cognition, perceptual speed, verbal and spatial ability | HDL-C (+ Verbal fluency [females], perceptual speed [males and females])  TG’s (+ Verbal fluency, spatial ability, perceptual speed, and general cognitive ability [females]) |  |
| Salama et al. | 2019 | Cross-sectional study | Egypt | Moderate | Total: n = 186; MCI: N = 14, Normal: N = 172  Age:  <50: N = 65  50 - <55: N = 64  55 - <60: N = 42  60-65: N = 15 | Global cognition | TC, HDL-C, LDL-C, TG |  |
| Salzwedel et al. | 2019 | Prospective, observational | Germany | High | n= 401 (Males= 321, Females= 80) Age= 54.5 (6.3) | Global cognition | Dyslipidaemia |  |
| Shi et al. | 2019 | Cross-sectional analysis of community-based, longitudinal study | BHS; USA | High | Total: n = 1177 (white male = 324, white female = 449, black male = 150, black female = 254)  Age:  Total = 48.11 (5.26)  White male = 48.92 (4.91)  White female = 48.15 (5.08)  Black male = 47.27 (6.03)  Black female = 47.53 (5.38) | Memory, attention, executive function, processing speed, global cognition | LDL |  |
| Sierra et al. | 2004 | Cross secticherbuinonal | Barcelona, Spain | High | *Without WML*: n= 37 (males= 24, females= 13); *With WML*: n= 23 (males= 14, females= 9) Age: *Without WML*= 53.9 (3.5); *With WML*= 55.2 (4.2) | Intelligence, memory, attention | TC |  |
| Suvila et al. | 2021 | Prospective | CARDIA, USA | High | n= 2,496 (males= 534, females= 1,689) Age= 55.1 (3.6) | Memory, executive function, psychomotor speed, global cognition | TC, HDL |  |
| Szczesnia et al. | 2020 | Longitudinal | PURE, Poland | High | n= 547 (males= 195, females= 352) Age= 56.2 (6.5) [males= 55.1 (6.8), females = 56.9 (6.3)] | Attention, executive function, psychomotor speed, global cognition | Hypercholesterolemia |  |
| Szoeke et al. | 2016 | Longitudinal, prospective | WHAP, Australia | Moderate | Baseline  n= 387  Age= 49.6 (2.5) | Memory | TC, HDL, TG |  |
| Szoeke et al. | 2019 | Longitudinal | WHAP; Australia | High | PET-SUVR data: n = 122  PET-SUVR >1.2: n = 23  PET-SUVR ≤1.2: n = 99  Age:  PET-SUVR data = 69.7 (2.5)  PET-SUVR >1.2 = 70.2 (2.5)  PET-SUVR ≤1.2 = 69.6 (2.5) | Global cognition | Lipid/Cholesterol lowering medication |  |
| Tufvesson et al. | 2013 | Prospective | MDCS, Sweden | High | n= 933 (males= 369, females= 564) Age= 57.5 (5.7) | Global cognition | HDL: + (Processing speed)  LDL, TG |  |
| Tuligenga et al. | 2014 | Prospective, longitudinal | Whitehall II study; UK | Moderate | Total: n = 5653; Normoglycaemia (n=4703); Prediabetes (n=648); Newly diagnosed diabetes (n=115); Known diabetes (n=187)  Age: Total = 54.4; Normoglycaemia = 55.1 (5.9); Prediabetes = 57.5 (6.1); Newly diagnosed diabetes 59.0 (6.1); Known diabetes = 57.4 (6.3) | Memory, executive function, and inductive reasoning | TC, Lipid/cholesterol lowering medication |  |
| Vadini et al. | 2020 | longitudinal, randomized, controlled, parallel-arm study | Italy | Moderate | Pre-liraglutide (n = 16) Pre-lifestyle (n = 16)  Age: Pre-liraglutide = 57 (49–64); Pre-lifestyle = 53 (52–58) | Memory, attention, executive function | TC, HDL-C, TG, Dyslipidemia |  |
| Veugen et al. | 2018 | Observational, prospective | Maastricht, Netherlands | High | n= 3,011 (males= 1,542, females = 1,469  Age= 52 (5) | Memory, executive function, attention | TC, HDL, LDL, TG, Lipid/Cholesterol lowering medication |  |
| Walker et al. | 2019 | Prospective | ARIC, USA | High | n= 3,012 (males= 1,382, females= 1,630)  Age= 55.5 (5.4) | Memory, executive function, psychomotor speed | TC, HDL, LDL, Lipid/Cholesterol lowering medication |  |
| Wang et al. | 2016 | Cross sectional | APAC, China | High | n= 3,048 (males= 1,727, females= 1,321) Age= 57.9 (11.1) | Global cognition | TC, HDL, LDL, TG, Hypercholesterolemia |  |
| Wang et al., | 2018 | Prospective epidemiological | ARIC, USA | High | n= 13,720  Age= ? | Memory, executive function, global cognition | TC, HDL, Lipid/Cholesterol lowering medication |  |
| Wendell et al. | 2014 | Prospective | BLSA, USA | High | n = 1601 (male = 813, female = 788)  Age = 54.4 (16.4) | Memory, attention, executive function, global cognition, and visuospatial ability | + TC (Memory, executive function, global cognition – midlife-later life) |  |
| Wharton et al. | 2014 | Cross sectional | KEEPSCog, USA | High | n= 571 (all females)  Age= 42–59 | Memory, attention, executive function, global cognition | HDL, LDL, TG |  |
| Wieczorek et al. | 2016 | Prospective | Poland | Moderate | n= 74 (males= 44, females= 30) Age= 59 (50–63) | Global cognition | Hypercholesterolemia |  |
| Wu et al. | 2022 | Cross sectional | CHARLS, China | Moderate | Diabetes Free: n = 7036; Treated Diabetes: n = 250; Untreated Diabetes: n = 628  Age: Diabetes Free:51.75 (4.81); Treated Diabetes: 53.35 (4.35); Untreated Diabetes: 52.05 (4.64) | Episodic memory, global cognition, mental intactness | Dylipidemia |  |
| Yano et al. | 2018 | Prospective | MACS, USA | High | n= 900 (all males) | Memory, attention, executive function, psychomotor speed | TC, HDL |  |
| Yano et al. | 2014 | Multicentre, longitudinal | CARDIA; USA | High | Total: n = 2326  Age at Year 25 = 50.4 (3.6) | Memory, executive function | TC, HDL |  |
| Ylilauri et al. | 2017 | Prospective | KIHD, Finland | High | n= 2,497 (all males)  Age= 42–60 | Global cognition, attention  executive function, memory | TC, HDL, LDL, TG, Lipid/Cholesterol lowering medication |  |
| Young et al. | 2006 | Longitudinal, observational | ARIC, USA | Moderate | n= 7,148 (males= 3,173, females= 3,975) Age= 53.7 | Memory, executive function | Hypercholesterolemia |  |

0, no association; +, positive association

**Abbreviations:** *ACE*, Akershus Cardiac Examination; *APAC,* Asymptomatic Polyvascular Abnormalities Community; *ARIC, Atherosclerosis Risk in Communities*; *ASCEND*, A Study of Cardiovascular Events in Diabetes; *Barcelona-AsIA*, Asymptomatic Intracranial Atherosclerosis; *BHS*, Bogalusa Heart Study; *BIP,* Bezafibrate Infarction Prevention; *BP,* blood pressure; *CARDIA,* Coronary Artery Risk Development in Young Adults; CHALICE, Canterbury Health, Ageing and Lifecourse; CHARLS, China Health and Retirement Longitudinal Study; *DBP*, diastolic blood pressure; *ELSA*, English Longitudinal Study of Ageing; ELSA, Brazilian Longitudinal Study of Adult Health *HAALSI*, Health and Aging in Africa; *HANDLS, healthy Aging in Neighborhoods of Diversity Across the Life Span*; *HHP*, Honolulu Heart Program; *KALS,* Kaohsiung Atherosclerosis Longitudinal Study ; *KEEPSCog*, Kronos Early Estrogen Prevention cognitive; *KIHD,* Kuopio Ischaemic Heart Disease Risk Factor Study; KIWI, Kinmen Women-Health Investigation; MACS*,* Multicentre AIDS Cohort Study; *MADT*, Middle-Aged Danish Twins; *MDCS,* Malmö Diet and Cancer Study; *MORGEN*, Monitoring Project on Cardiovascular Disease Risk Factors; *MRC,* Medical Research Council; NCDS, National Child Development Study; NSHD*,* National Survey of Health and Development; *PATH,* Population Assessment of Tobacco and Health*; PURE,* prospective Urban and Rural Epidemiological; *RECALL*, Risk Factors, Evaluation of Coronary Calcium and Lifestyle; *SBP*, systolic blood pressure; *Swan,* Study of Women’s Health Across the Nation; TILDA, The Irish Longitudinal Study on Ageing; *VETSA,* Vietnam Era Twin Study of Aging and; *WHAP,* Women’s Health Aging Project

***Supplemental Table B.3.*** Summary of negative relationships between cholesterol metrics and cognition at midlife by study design and quality.

| Study Design | Memory | Attention | Executive Function | Global Cognition | Psychomotor Speed | Inductive Reasoning | Intelligence | Visuospatial Organisation |
| --- | --- | --- | --- | --- | --- | --- | --- | --- |
| TC |  |  |  |  |  |  |  |  |
| *Individual Study Cohorts* | (15, 48-51) | (51) | (15, 37, 40, 49) | (37) | - | - | - | - |
| *Longitudinal Study Cohorts* | (32, 52) | - | (32, 35, 52-56) | (32, 35, 52, 55, 57, 58) | - | (35, 55) | - | - |
| *Study Quality (n=)* | Low: 1  Moderate: 5  High: 1 | Low: -  Moderate: 1  High: - | Low: 1  Moderate: 7  High: 3 | Low: 1  Moderate: 3  High: 3 | Low: -  Moderate: -  High: - | Low: -  Moderate: 1  High: 1 | Low: -  Moderate: -  High: - | Low: -  Moderate: -  High: - |
| HDL-C |  |  |  |  |  |  |  |  |
| *Individual Study Cohorts* | (49, 59) | (59) | (49) | (59) | - | - | - | (59) |
| *Longitudinal Study Cohorts* | (60, 61) | - | (32, 60-62) | - | - | (61) | - | - |
| *Study Quality (n=)* | Low: -  Moderate: 2  High: 2 | Low: -  Moderate: -  High: 1 | Low: -  Moderate: 2  High: 3 | Low: -  Moderate: -  High: 1 | Low: -  Moderate: -  High: - | Low: -  Moderate: 1  High: - | Low: -  Moderate: -  High: - | Low: -  Moderate: -  High: 1 |
| LDL-C |  |  |  |  |  |  |  |  |
| *Individual Study Cohorts* | (38, 59, 63) | (59, 63) | (63) | (59) | (63) | - | - | (59) |
| *Longitudinal Study Cohorts* | (64, 65) | - | (32, 62) | - | - | - | - | - |
| *Study Quality (n=)* | Low: 1  Moderate: 3  High: 1 | Low: -  Moderate: 1  High: 1 | Low: -  Moderate: 1  High: 2 | Low: -  Moderate: -  High: 1 | Low: -  Moderate: 1  High: - | Low: -  Moderate: -  High: - | Low: -  Moderate: -  High: - | Low: -  Moderate: -  High: 1 |
| TG |  |  |  |  |  |  |  |  |
| *Individual Study Cohorts* | (59) | (59) | - | (40, 59) | - | - | - | (59) |
| *Longitudinal Study Cohorts* | (32, 60) | - | (32, 60) | (32) | - | - | - | - |
| *Study Quality (n=)* | Low: -  Moderate: -  High: 3 | Low: -  Moderate: -  High: 1 | Low: -  Moderate: -  High: 2 | Low: -  Moderate: 1  High: 2 | Low: -  Moderate: -  High: - | Low: -  Moderate: -  High: - | Low: -  Moderate: -  High: - | Low: -  Moderate: -  High: 1 |
| Hypercholesterolemia |  |  |  |  |  |  |  |  |
| *Individual Study Cohorts* | (41, 66) | - | (66) | (67) | - | - | - | - |
| *Longitudinal Study Cohorts* | (68, 69) | - | (68, 69) | (69) | - | - | - | - |
| *Study Quality (n=)* | Low: 2  Moderate: 1  High: 1 | Low: -  Moderate: -  High: - | Low: 2  Moderate: -  High: 1 | Low: 1  Moderate: 1  High: - | Low: -  Moderate: -  High: - | Low: -  Moderate: -  High: - | Low: -  Moderate: -  High: - | Low: -  Moderate: -  High: - |
| Lipid Lowering Medication |  |  |  |  |  |  |  |  |
| *Individual Study Cohorts* | (15) | - | - | - | (15) | - | - | - |
| *Longitudinal Study Cohorts* | - | - | - | (70) | - | - | - | - |
| *Study Quality (n=)* | Low: 1  Moderate: -  High: - | Low: -  Moderate: -  High: - | Low: -  Moderate: -  High: - | Low: -  Moderate: -  High: 1 | Low: 1  Moderate: -  High: - | Low: -  Moderate: -  High: - | Low: -  Moderate: -  High: - | Low: -  Moderate: -  High: - |
| Dyslipidemia |  |  |  |  |  |  |  |  |
| *Individual Study Cohorts* | - | - | (71) | (71-73) | - | - | - | - |
| *Longitudinal Study Cohorts* | - | - | - | - | - | - | - | - |
| *Study Quality (n=)* | Low: -  Moderate: -  High: - | Low: -  Moderate: -  High: - | Low: -  Moderate: -  High: 1 | Low: -  Moderate: 2  High: 1 | Low: -  Moderate: -  High: - | Low: -  Moderate: -  High: - | Low: -  Moderate: -  High: - | Low: -  Moderate: -  High: - |

***Supplemental Table B.4.*** Critical appraisal of included studies using the AXIS tool.

| **Question** | Albanese et al. 2012 | Aliberti et al., 2020 | Alves de Moraes et al. 2002 | Anstey et al.2014 | Babaei et al.2013 | Backestrom et al. 2015 | Bancks et al. 2017 | Boots et al. 2015 | Bressler et al. 2013 | Britton et al. 2004 | Brunner et al. 2017 |
| --- | --- | --- | --- | --- | --- | --- | --- | --- | --- | --- | --- |
| **Introduction** | | | | | | | | | | | |
| Question 1 | **Y** | **Y** | **N** | **Y** | **Y** | **Y** | **Y** | **Y** | **N** | **Y** | **U** |
| **Methods** | | | | | | | | | | | |
| Question 2 | **Y** | **Y** | **Y** | **Y** | **Y** | **Y** | **Y** | **Y** | **Y** | **Y** | **U** |
| Question 3 | **N** | **N** | **N** | **N** | **N** | **N** | **N** | **N** | **N** | **N** | **U** |
| Question 4 | **Y** | **Y** | **Y** | **Y** | **Y** | **Y** | **Y** | **Y** | **Y** | **Y** | **Y** |
| Question 5 | **Y** | **Y** | **Y** | **Y** | **Y** | **Y** | **Y** | **Y** | **Y** | **Y** | **Y** |
| Question 6 | **Y** | **Y** | **Y** | **Y** | **U** | **Y** | **Y** | **Y** | **Y** | **Y** | **Y** |
| Question 7 | **N** | **Y** | **N** | **N** | **U** | **N** | **N** | **N** | **N** | **N** | **N** |
| Question 8 | **Y** | **Y** | **Y** | **Y** | **Y** | **Y** | **Y** | **Y** | **Y** | **Y** | **Y** |
| Question 9 | **Y** | **Y** | **Y** | **Y** | **Y** | **Y** | **Y** | **Y** | **Y** | **Y** | **Y** |
| Question 10 | **U** | **Y** | **Y** | **Y** | **Y** | **Y** | **Y** | **Y** | **Y** | **N** | **U** |
| Question 11 | **Y** | **Y** | **Y** | **Y** | **N** | **Y** | **Y** | **Y** | **Y** | **N** | **Y** |
| **Results** | | | | | | | | | | | |
| Question 12 | **Y** | **Y** | **Y** | **Y** | **N** | **Y** | **Y** | **Y** | **Y** | **Y** | **Y** |
| Question 13 | **U** | **N** | **N** | **N** | **U** | **N** | **N** | **N** | **N** | **N** | **N** |
| Question 14 | **N** | **U** | **Y** | **N** | **U** | **N** | **N** | **N** | **N** | **N** | **N** |
| Question 15 | **Y** | **Y** | **Y** | **Y** | **Y** | **Y** | **Y** | **Y** | **Y** | **Y** | **Y** |
| Question 16 | **Y** | **Y** | **Y** | **Y** | **Y** | **Y** | **Y** | **Y** | **Y** | **Y** | **Y** |
| **Discussion** | | | | | | | | | | | |
| Question 17 | **Y** | **Y** | **Y** | **Y** | **N** | **Y** | **Y** | **Y** | **Y** | **Y** | **Y** |
| Question 18 | **N** | **Y** | **Y** | **N** | **N** | **Y** | **Y** | **Y** | **N** | **Y** | **N** |
| **Other** | | | | | | | | | | | |
| Question 19 | **N** | **N** | **U** | **N** | **N** | **N** | **N** | **N** | **N** | **U** | **N** |
| Question 20 | **Y** | **Y** | **U** | **Y** | **Y** | **Y** | **Y** | **Y** | **Y** | **Y** | **N** |

| **Question** | | Carmichael et al. 2019 | Cerhan et al. 1998 | Chen et al. 2015 | Cherbuin et al. 2009 | Chosy et al. 2019 | Christman et al. 2011 | Chuang et al. 2023 | Cohen-Manheim et al. 2016 | Creavin et al. 2012 | Cui et al. 2016 | de Menezes et al. 2021 | Dearborn et al. 2014 |  |
| --- | --- | --- | --- | --- | --- | --- | --- | --- | --- | --- | --- | --- | --- | --- |
|  | **Introduction** | | | | | | | | | | | | | |
| Question 1 | | **Y** | **Y** | **Y** | **Y** | **Y** | **Y** | **Y** | **Y** | **Y** | **Y** | **Y** | **Y** |  |
|  | **Methods** | | | | | | | | | | | | | |
| Question 2 | | **Y** | **Y** | **Y** | **Y** | **Y** | **Y** | **Y** | **Y** | **Y** | **Y** | **Y** | **Y** |  |
| Question 3 | | **N** | **Y** | **N** | **N** | **N** | **N** | **N** | **Y** | **N** | **N** | **N** | **N** |  |
| Question 4 | | **Y** | **Y** | **Y** | **Y** | **Y** | **Y** | **Y** | **Y** | **Y** | **Y** | **Y** | **Y** |  |
| Question 5 | | **Y** | **Y** | **Y** | **Y** | **Y** | **Y** | **Y** | **Y** | **Y** | **Y** | **Y** | **Y** |  |
| Question 6 | | **Y** | **Y** | **Y** | **Y** | **Y** | **Y** | **Y** | **Y** | **Y** | **Y** | **Y** | **Y** |  |
| Question 7 | | **N** | **N** | **N** | **N** | **N** | **N** | **N** | **N** | **N** | **N** | **N** | **N** |  |
| Question 8 | | **Y** | **Y** | **Y** | **Y** | **Y** | **Y** | **Y** | **Y** | **Y** | **Y** | **Y** | **Y** |  |
| Question 9 | | **Y** | **Y** | **Y** | **Y** | **Y** | **Y** | **Y** | **Y** | **Y** | **Y** | **Y** | **Y** |  |
| Question 10 | | **N** | **N** | **Y** | **Y** | **N** | **Y** | **Y** | **Y** | **N** | **Y** | **Y** | **Y** |  |
| Question 11 | | **Y** | **Y** | **Y** | **Y** | **Y** | **Y** | **Y** | **Y** | **Y** | **Y** | **Y** | **Y** |  |
|  | **Results** | | | | | | | | | | | | | |
| Question 12 | | **Y** | **Y** | **Y** | **Y** | **Y** | **Y** | **Y** | **Y** | **Y** | **Y** | **Y** | **Y** |  |
| Question 13 | | **N** | **N** | **N** | **N** | **N** | **N** | **N** | **N** | **N** | **N** | **N** | **N** |  |
| Question 14 | | **N** | **N** | **N** | **N** | **N** | **N** | **N** | **N** | **N** | **N** | **N** | **N** |  |
| Question 15 | | **Y** | **Y** | **Y** | **Y** | **Y** | **Y** | **Y** | **Y** | **Y** | **Y** | **Y** | **Y** |  |
| Question 16 | | **Y** | **Y** | **Y** | **Y** | **Y** | **Y** | **Y** | **Y** | **Y** | **Y** | **Y** | **Y** |  |
|  | **Discussion** | | | | | | | | | | | | | |
| Question 17 | | **Y** | **Y** | **Y** | **Y** | **Y** | **Y** | **Y** | **Y** | **Y** | **Y** | **Y** | **Y** |  |
| Question 18 | | **Y** | **Y** | **Y** | **Y** | **U** | **Y** | **Y** | **N** | **Y** | **N** | **Y** | **Y** |  |
|  | **Other** | | | | | | | | | | | | | |
| Question 19 | | **N** | **N** | **N** | **N** | **N** | **N** | **N** | **N** | **N** | **N** | **N** | **N** |  |
| Question 20 | | **Y** | **Y** | **Y** | **Y** | **Y** | **U** | **Y** | **Y** | **Y** | **Y** | **Y** | **Y** |  |

| **Question** | | Dearborn-Tomazos et al. 2019 | Debette et al. 2011 | Del Vecchio et al. 2023 | Derby et al. 2021 | Elbaz et al. 2014 | Elkins et al. 2005 | Fava et al. 2013 | Ferguson et al. 2018 | Fuh et al. 2007 | Gerber et al. 2021 | Giugliano et al. 2018 |  |
| --- | --- | --- | --- | --- | --- | --- | --- | --- | --- | --- | --- | --- | --- |
|  | **Introduction** | | | | | | | | | | | | |
| Question 1 | | **Y** | **Y** | **Y** | **Y** | **Y** | **Y** | **Y** | **Y** | **Y** | **N** | **N** |  |
|  | **Methods** | | | | | | | | | | | | |
| Question 2 | | **Y** | **Y** | **Y** | **Y** | **Y** | **Y** | **Y** | **Y** | **Y** | **N** | **N** |  |
| Question 3 | | **N** | **N** | **N** | **Y** | **N** | **N** | **N** | **N** | **N** | **N** | **N** |  |
| Question 4 | | **Y** | **Y** | **Y** | **N** | **Y** | **Y** | **Y** | **Y** | **N** | **Y** | **Y** |  |
| Question 5 | | **Y** | **Y** | **Y** | **Y** | **Y** | **Y** | **Y** | **Y** | **Y** | **Y** | **U** |  |
| Question 6 | | **Y** | **Y** | **Y** | **Y** | **Y** | **Y** | **Y** | **Y** | **Y** | **Y** | **Y** |  |
| Question 7 | | **N** | **N** | **N** | **N** | **N** | **N** | **N** | **N** | **N** | **Y** | **N** |  |
| Question 8 | | **Y** | **Y** | **Y** | **Y** | **Y** | **Y** | **Y** | **Y** | **Y** | **Y** | **Y** |  |
| Question 9 | | **Y** | **Y** | **Y** | **Y** | **Y** | **Y** | **Y** | **Y** | **Y** | **Y** | **Y** |  |
| Question 10 | | **Y** | **N** | **Y** | **N** | **Y** | **Y** | **Y** | **N** | **Y** | **Y** | **Y** |  |
| Question 11 | | **N** | **Y** | **Y** | **Y** | **Y** | **Y** | **Y** | **Y** | **Y** | **Y** | **Y** |  |
|  | **Results** | | | | | | | | | | | | |
| Question 12 | | **N** | **Y** | **Y** | **Y** | **Y** | **Y** | **Y** | **Y** | **Y** | **Y** | **Y** |  |
| Question 13 | | **N** | **N** | **N** | **N** | **N** | **N** | **N** | **N** | **N** | **N** | **N** |  |
| Question 14 | | **N** | **N** | **N** | **N** | **N** | **N** | **N** | **N** | **N** | **N** | **N** |  |
| Question 15 | | **Y** | **Y** | **Y** | **Y** | **Y** | **Y** | **Y** | **Y** | **Y** | **Y** | **Y** |  |
| Question 16 | | **Y** | **Y** | **Y** | **Y** | **Y** | **Y** | **Y** | **Y** | **Y** | **Y** | **Y** |  |
|  | **Discussion** | | | | | | | | | | | | |
| Question 17 | | **Y** | **Y** | **Y** | **Y** | **Y** | **Y** | **Y** | **Y** | **Y** | **Y** | **Y** |  |
| Question 18 | | **Y** | **Y** | **Y** | **Y** | **Y** | **Y** | **Y** | **Y** | **N** | **N** | **N** |  |
|  | **Other** | | | | | | | | | | | | |
| Question 19 | | **N** | **N** | **N** | **N** | **N** | **N** | **N** | **N** | **N** | **N** | **N** |  |
| Question 20 | | **Y** | **U** | **Y** | **Y** | **Y** | **Y** | **N** | **Y** | **Y** | **Y** | **Y** |  |

| **Question** | Gonzalez et al. 2018 | Gottesman et al. 2017 | Gourley et al. 2020 | Hajjar et al. 2018 | Hakamada-Taguchi et al. 2002 | Haley et al. 2010 | Henderson et al. 2003 | Henriksen et al. 2017 | Hossain et al. 2020 | Houle et al. 2019 |  |
| --- | --- | --- | --- | --- | --- | --- | --- | --- | --- | --- | --- |
| **Introduction** | | | | | | | | | | | |
| Question 1 | **Y** | **Y** | **Y** | **Y** | **Y** | **Y** | **Y** | **Y** | **Y** | **Y** |  |
| **Methods** | | | | | | | | | | | |
| Question 2 | **Y** | **Y** | **Y** | **Y** | **Y** | **Y** | **Y** | **Y** | **Y** | **Y** |  |
| Question 3 | **N** | **N** | **N** | **N** | **N** | **N** | **N** | **N** | **N** | **N** |  |
| Question 4 | **Y** | **Y** | **Y** | **Y** | **Y** | **Y** | **Y** | **Y** | **Y** | **Y** |  |
| Question 5 | **Y** | **Y** | **Y** | **Y** | **Y** | **Y** | **Y** | **Y** | **Y** | **Y** |  |
| Question 6 | **Y** | **Y** | **Y** | **Y** | **U** | **Y** | **Y** | **Y** | **Y** | **Y** |  |
| Question 7 | **N** | **N** | **N** | **N** | **N** | **N** | **Y** | **N** | **N** | **N** |  |
| Question 8 | **Y** | **Y** | **Y** | **Y** | **Y** | **Y** | **Y** | **Y** | **Y** | **Y** |  |
| Question 9 | **Y** | **Y** | **Y** | **Y** | **Y** | **Y** | **Y** | **Y** | **Y** | **Y** |  |
| Question 10 | **Y** | **Y** | **N** | **N** | **N** | **N** | **N** | **Y** | **Y** | **N** |  |
| Question 11 | **Y** | **Y** | **Y** | **Y** | **N** | **Y** | **Y** | **Y** | **Y** | **Y** |  |
| **Results** | | | | | | | | | | | |
| Question 12 | **Y** | **Y** | **Y** | **Y** | **Y** | **Y** | **Y** | **Y** | **Y** | **Y** |  |
| Question 13 | **N** | **N** | **N** | **N** | **N** | **N** | **N** | **N** | **N** | **N** |  |
| Question 14 | **N** | **N** | **N** | **N** | **N** | **N** | **Y** | **N** | **N** | **N** |  |
| Question 15 | **Y** | **Y** | **Y** | **Y** | **Y** | **Y** | **Y** | **Y** | **Y** | **Y** |  |
| Question 16 | **Y** | **Y** | **Y** | **Y** | **Y** | **Y** | **Y** | **Y** | **Y** | **Y** |  |
| **Discussion** | | | | | | | | | | | |
| Question 17 | **Y** | **Y** | **Y** | **Y** | **Y** | **Y** | **Y** | **Y** | **Y** | **Y** |  |
| Question 18 | **Y** | **Y** | **Y** | **Y** | **Y** | **Y** | **N** | **Y** | **Y** | **Y** |  |
| **Other** | | | | | | | | | | | |
| Question 19 | **N** | **N** | **N** | **N** | **N** | **N** | **N** | **N** | **N** | **N** |  |
| Question 20 | **U** | **Y** | **Y** | **Y** | **Y** | **Y** | **Y** | **Y** | **Y** | **Y** |  |

| **Question** | Ihle-Hansen et al. 2019 | John et al. 2021 | Kaffashian et al. 2011 | Kaffashian et al.2011 | Kalmijn et al. 2002 | Kivipelto et al. 2001 | Knopman et al. 2001 | Knopman et al.2018 | Knopman et al. 2009 | Kovacs et al. 2014 |  |
| --- | --- | --- | --- | --- | --- | --- | --- | --- | --- | --- | --- |
| **Introduction** | | | | | | | | | | | |
| Question 1 | **Y** | **Y** | **Y** | **Y** | **Y** | **Y** | **Y** | **Y** | **Y** | **Y** |  |
| **Methods** | | | | | | | | | | | |
| Question 2 | **Y** | **Y** | **Y** | **Y** | **Y** | **Y** | **Y** | **Y** | **Y** | **Y** |  |
| Question 3 | **N** | **Y** | **N** | **N** | **N** | **Y** | **N** | **N** | **N** | **N** |  |
| Question 4 | **Y** | **Y** | **Y** | **Y** | **Y** | **Y** | **Y** | **Y** | **Y** | **Y** |  |
| Question 5 | **Y** | **Y** | **Y** | **Y** | **Y** | **Y** | **Y** | **Y** | **Y** | **Y** |  |
| Question 6 | **Y** | **Y** | **Y** | **Y** | **Y** | **Y** | **Y** | **Y** | **Y** | **Y** |  |
| Question 7 | **N** | **Y** | **N** | **N** | **N** | **Y** | **N** | **N** | **N** | **N** |  |
| Question 8 | **Y** | **Y** | **Y** | **Y** | **Y** | **Y** | **Y** | **Y** | **Y** | **Y** |  |
| Question 9 | **Y** | **Y** | **Y** | **Y** | **Y** | **Y** | **Y** | **Y** | **Y** | **Y** |  |
| Question 10 | **Y** | **N** | **Y** | **Y** | **N** | **Y** | **N** | **N** | **N** | **Y** |  |
| Question 11 | **Y** | **Y** | **Y** | **Y** | **Y** | **Y** | **Y** | **Y** | **Y** | **Y** |  |
| **Results** | | | | | | | | | | | |
| Question 12 | **Y** | **Y** | **Y** | **Y** | **Y** | **Y** | **Y** | **N** | **Y** | **Y** |  |
| Question 13 | **N** | **N** | **N** | **N** | **N** | **N** | **N** | **N** | **N** | **N** |  |
| Question 14 | **N** | **N** | **N** | **N** | **N** | **Y** | **N** | **N** | **N** | **N** |  |
| Question 15 | **Y** | **Y** | **Y** | **Y** | **N** | **Y** | **Y** | **Y** | **Y** | **Y** |  |
| Question 16 | **Y** | **Y** | **Y** | **Y** | **Y** | **Y** | **Y** | **Y** | **Y** | **Y** |  |
| **Discussion** | | | | | | | | | | | |
| Question 17 | **Y** | **Y** | **Y** | **Y** | **Y** | **Y** | **Y** | **Y** | **Y** | **Y** |  |
| Question 18 | **Y** | **Y** | **Y** | **Y** | **Y** | **Y** | **Y** | **N** | **Y** | **N** |  |
| **Other** | | | | | | | | | | | |
| Question 19 | **N** | **N** | **N** | **N** | **N** | **N** | **N** | **N** | **N** | **N** |  |
| Question 20 | **Y** | **Y** | **Y** | **Y** | **Y** | **Y** | **Y** | **Y** | **N** | **Y** |  |

| **Question** | Kumar et al.,2020 | Kumar et al. 2008 | Kumari et al., 2005 | Lin et al. 2020 | Liu et al. 2022 | Lopez-Oloriz et al. 2014 | Lutski et al. 2019 | Mahinrad et al. 2020 | Masi et al. 2018 | Mefford et al. 2021 |  |
| --- | --- | --- | --- | --- | --- | --- | --- | --- | --- | --- | --- |
| **Introduction** | | | | | | | | | | | |
| Question 1 | **Y** | **Y** | **Y** | **Y** | **Y** | **Y** | **Y** | **Y** | **Y** | **Y** |  |
| **Methods** | | | | | | | | | | | |
| Question 2 | **Y** | **Y** | **Y** | **Y** | **Y** | **Y** | **Y** | **Y** | **Y** | **Y** |  |
| Question 3 | **N** | **N** | **N** | **N** | **N** | **N** | **N** | **N** | **N** | **N** |  |
| Question 4 | **Y** | **Y** | **Y** | **Y** | **Y** | **Y** | **Y** | **Y** | **Y** | **Y** |  |
| Question 5 | **Y** | **Y** | **Y** | **Y** | **Y** | **Y** | **Y** | **Y** | **U** | **Y** |  |
| Question 6 | **Y** | **Y** | **Y** | **Y** | **Y** | **Y** | **Y** | **Y** | **U** | **Y** |  |
| Question 7 | **N** | **N** | **N** | **Y** | **N** | **N** | **Y** | **N** | **N** | **N** |  |
| Question 8 | **Y** | **Y** | **Y** | **Y** | **Y** | **Y** | **Y** | **Y** | **Y** | **Y** |  |
| Question 9 | **Y** | **Y** | **Y** | **Y** | **Y** | **Y** | **Y** | **Y** | **Y** | **Y** |  |
| Question 10 | **N** | **Y** | **Y** | **Y** | **Y** | **N** | **N** | **Y** | **N** | **Y** |  |
| Question 11 | **Y** | **Y** | **Y** | **Y** | **Y** | **N** | **Y** | **Y** | **N** | **Y** |  |
| **Results** | | | | | | | | | | | |
| Question 12 | **Y** | **Y** | **Y** | **Y** | **Y** | **Y** | **Y** | **Y** | **N** | **Y** |  |
| Question 13 | **N** | **N** | **N** | **N** | **N** | **N** | **N** | **N** | **N** | **N** |  |
| Question 14 | **N** | **N** | **N** | **N** | **N** | **N** | **Y** | **N** | **N** | **N** |  |
| Question 15 | **Y** | **Y** | **Y** | **Y** | **Y** | **Y** | **Y** | **Y** | **U** | **Y** |  |
| Question 16 | **Y** | **Y** | **Y** | **Y** | **Y** | **Y** | **Y** | **Y** | **Y** | **Y** |  |
| **Discussion** | | | | | | | | | | | |
| Question 17 | **Y** | **Y** | **Y** | **Y** | **Y** | **Y** | **Y** | **Y** | **U** | **Y** |  |
| Question 18 | **N** | **Y** | **Y** | **Y** | **Y** | **Y** | **Y** | **Y** | **N** | **Y** |  |
| **Other** | | | | | | | | | | | |
| Question 19 | **N** | **N** | **N** | **N** | **N** | **N** | **N** | **N** | **U** | **N** |  |
| Question 20 | **N** | **Y** | **N** | **Y** | **Y** | **Y** | **Y** | **Y** | **N** | **Y** |  |

| **Question** | Moore et al. 2014 | Muhkerjee et al. 2022 | Mukerji et al. 2016 | Nunley et al. 2017 | Palacios-Mendoza et al. 2018 | Panigrahi et al., 2021 | Pearson et al. 2017 | Power et al., 2017 | Rask et al. 2016 | Ravona-Springer et al. 2020 |  |
| --- | --- | --- | --- | --- | --- | --- | --- | --- | --- | --- | --- |
| **Introduction** | | | | | | | | | | | |
| Question 1 | **Y** | **Y** | **Y** | **Y** | **Y** | **Y** | **Y** | **Y** | **Y** | **Y** |  |
| **Methods** | | | | | | | | | | | |
| Question 2 | **Y** | **Y** | **Y** | **Y** | **Y** | **Y** | **Y** | **Y** | **Y** | **Y** |  |
| Question 3 | **N** | **Y** | **Y** | **N** | **N** | **N** | **Y** | **N** | **N** | **N** |  |
| Question 4 | **Y** | **Y** | **Y** | **Y** | **Y** | **Y** | **Y** | **Y** | **Y** | **Y** |  |
| Question 5 | **Y** | **Y** | **Y** | **Y** | **Y** | **Y** | **Y** | **Y** | **Y** | **Y** |  |
| Question 6 | **Y** | **Y** | **Y** | **Y** | **Y** | **Y** | **Y** | **Y** | **Y** | **Y** |  |
| Question 7 | **N** | **N** | **N** | **Y** | **N** | **N** | **N** | **N** | **Y** | **N** |  |
| Question 8 | **Y** | **Y** | **Y** | **Y** | **Y** | **Y** | **Y** | **Y** | **Y** | **Y** |  |
| Question 9 | **Y** | **Y** | **Y** | **Y** | **Y** | **Y** | **Y** | **Y** | **Y** | **Y** |  |
| Question 10 | **Y** | **N** | **N** | **Y** | **Y** | **N** | **Y** | **Y** | **N** | **N** |  |
| Question 11 | **Y** | **Y** | **Y** | **Y** | **Y** | **Y** | **Y** | **Y** | **Y** | **Y** |  |
| **Results** | | | | | | | | | | | |
| Question 12 | **Y** | **Y** | **Y** | **Y** | **Y** | **Y** | **Y** | **Y** | **Y** | **Y** |  |
| Question 13 | **N** | **N** | **N** | **N** | **N** | **N** | **N** | **N** | **N** | **N** |  |
| Question 14 | **Y** | **N** | **N** | **N** | **N** | **N** | **N** | **N** | **Y** | **N** |  |
| Question 15 | **Y** | **Y** | **Y** | **Y** | **Y** | **Y** | **Y** | **Y** | **Y** | **Y** |  |
| Question 16 | **Y** | **Y** | **Y** | **Y** | **Y** | **Y** | **Y** | **Y** | **Y** | **Y** |  |
| **Discussion** | | | | | | | | | | | |
| Question 17 | **Y** | **Y** | **Y** | **Y** | **Y** | **Y** | **Y** | **Y** | **Y** | **Y** |  |
| Question 18 | **Y** | **Y** | **Y** | **Y** | **Y** | **Y** | **Y** | **Y** | **Y** | **Y** |  |
| **Other** | | | | | | | | | | | |
| Question 19 | **N** | **N** | **N** | **N** | **N** | **N** | **N** | **N** | **N** | **N** |  |
| Question 20 | **Y** | **Y** | **Y** | **Y** | **Y** | **Y** | **Y** | **Y** | **Y** | **Y** |  |

| **Question** | Rawlings et al., 2014 | Razavi et al., 2020 | Reis et al. 2013 | Reynolds et al. 2010 | Salama et al. 2019 | Salzwedel et al. 2019 | Sha et al. 2018 | Sierra et al. 2004 | Singh-Manoux et al. 2003 | Solomon et al.2009 |  |
| --- | --- | --- | --- | --- | --- | --- | --- | --- | --- | --- | --- |
| **Introduction** | | | | | | | | | | | |
| Question 1 | **Y** | **Y** | **Y** | **Y** | **Y** | **Y** | **Y** | **Y** | **Y** | **Y** |  |
| **Methods** | | | | | | | | | | | |
| Question 2 | **Y** | **Y** | **Y** | **Y** | **Y** | **Y** | **Y** | **Y** | **Y** | **Y** |  |
| Question 3 | **N** | **N** | **N** | **Y** | **N** | **N** | **Y** | **N** | **N** | **N** |  |
| Question 4 | **Y** | **Y** | **Y** | **Y** | **Y** | **Y** | **Y** | **Y** | **Y** | **Y** |  |
| Question 5 | **Y** | **Y** | **Y** | **Y** | **Y** | **Y** | **Y** | **Y** | **Y** | **U** |  |
| Question 6 | **Y** | **Y** | **Y** | **Y** | **Y** | **Y** | **Y** | **Y** | **Y** | **U** |  |
| Question 7 | **N** | **N** | **N** | **N** | **N** | **N** | **N** | **N** | **N** | **N** |  |
| Question 8 | **Y** | **Y** | **Y** | **Y** | **Y** | **Y** | **Y** | **Y** | **Y** | **Y** |  |
| Question 9 | **Y** | **Y** | **Y** | **Y** | **Y** | **Y** | **Y** | **Y** | **Y** | **Y** |  |
| Question 10 | **N** | **Y** | **Y** | **N** | **Y** | **Y** | **N** | **Y** | **Y** | **Y** |  |
| Question 11 | **Y** | **Y** | **Y** | **Y** | **Y** | **Y** | **Y** | **Y** | **Y** | **Y** |  |
| **Results** | | | | | | | | | | | |
| Question 12 | **Y** | **Y** | **Y** | **Y** | **Y** | **Y** | **Y** | **Y** | **Y** | **Y** |  |
| Question 13 | **N** | **N** | **N** | **N** | **N** | **N** | **N** | **N** | **N** | **N** |  |
| Question 14 | **N** | **N** | **N** | **N** | **N** | **N** | **N** | **N** | **N** | **N** |  |
| Question 15 | **Y** | **Y** | **Y** | **Y** | **Y** | **Y** | **Y** | **Y** | **Y** | **Y** |  |
| Question 16 | **Y** | **Y** | **Y** | **Y** | **Y** | **Y** | **Y** | **Y** | **Y** | **Y** |  |
| **Discussion** | | | | | | | | | | | |
| Question 17 | **Y** | **Y** | **Y** | **Y** | **Y** | **Y** | **Y** | **Y** | **Y** | **Y** |  |
| Question 18 | **Y** | **Y** | **Y** | **Y** | **Y** | **Y** | **Y** | **Y** | **Y** | **N** |  |
| **Other** | | | | | | | | | | | |
| Question 19 | **N** | **N** | **N** | **N** | **N** | **N** | **N** | **N** | **N** | **N** |  |
| Question 20 | **N** | **Y** | **Y** | **N** | **Y** | **Y** | **Y** | **Y** | **Y** | **Y** |  |

| **Question** | Suvila et al. 2021 | Szczesnia et al. 2020 | Szoeke et al. 2016 | Szoeke et al. 2016 | Tufvesson et al. 2013 | Tuligenga et al. 2014 | Vadini et al. 2020 | Veugen et al. 2018 | Walker et al. 2019 | Wang et al. 2016 |  |
| --- | --- | --- | --- | --- | --- | --- | --- | --- | --- | --- | --- |
| **Introduction** | | | | | | | | | | | |
| Question 1 | **Y** | **Y** | **Y** | **Y** | **Y** | **Y** | **Y** | **Y** | **Y** | **Y** |  |
| **Methods** | | | | | | | | | | | |
| Question 2 | **Y** | **Y** | **Y** | **Y** | **Y** | **Y** | **Y** | **Y** | **Y** | **Y** |  |
| Question 3 | **N** | **Y** | **N** | **N** | **N** | **N** | **N** | **N** | **N** | **N** |  |
| Question 4 | **Y** | **Y** | **Y** | **Y** | **Y** | **Y** | **Y** | **Y** | **Y** | **Y** |  |
| Question 5 | **Y** | **Y** | **Y** | **Y** | **Y** | **Y** | **Y** | **Y** | **Y** | **Y** |  |
| Question 6 | **Y** | **Y** | **Y** | **Y** | **Y** | **Y** | **Y** | **Y** | **Y** | **Y** |  |
| Question 7 | **N** | **N** | **Y** | **Y** | **Y** | **N** | **N** | **N** | **Y** | **N** |  |
| Question 8 | **Y** | **Y** | **Y** | **Y** | **Y** | **Y** | **Y** | **Y** | **Y** | **Y** |  |
| Question 9 | **Y** | **Y** | **Y** | **Y** | **Y** | **Y** | **Y** | **Y** | **Y** | **Y** |  |
| Question 10 | **Y** | **Y** | **Y** | **Y** | **Y** | **Y** | **Y** | **Y** | **Y** | **Y** |  |
| Question 11 | **Y** | **Y** | **Y** | **Y** | **Y** | **Y** | **Y** | **Y** | **Y** | **Y** |  |
| **Results** | | | | | | | | | | | |
| Question 12 | **Y** | **Y** | **Y** | **Y** | **Y** | **Y** | **Y** | **Y** | **Y** | **Y** |  |
| Question 13 | **N** | **N** | **N** | **N** | **N** | **N** | **N** | **N** | **N** | **N** |  |
| Question 14 | **N** | **N** | **N** | **N** | **N** | **N** | **N** | **N** | **N** | **N** |  |
| Question 15 | **Y** | **Y** | **Y** | **Y** | **Y** | **Y** | **Y** | **Y** | **Y** | **Y** |  |
| Question 16 | **Y** | **Y** | **Y** | **Y** | **Y** | **Y** | **Y** | **Y** | **Y** | **Y** |  |
| **Discussion** | | | | | | | | | | | |
| Question 17 | **Y** | **Y** | **Y** | **Y** | **Y** | **Y** | **Y** | **Y** | **Y** | **Y** |  |
| Question 18 | **Y** | **Y** | **Y** | **Y** | **Y** | **Y** | **Y** | **Y** | **Y** | **Y** |  |
| **Other** | | | | | | | | | | | |
| Question 19 | **N** | **N** | **N** | **N** | **N** | **N** | **N** | **N** | **N** | **N** |  |
| Question 20 | **Y** | **Y** | **N** | **Y** | **Y** | **Y** | **Y** | **Y** | **Y** | **Y** |  |

| **Question** | | Wang et al., 2018 | Wendell et al. 2016 | Wharton et al., 2014 | Wieczorek et al. 2016 | Wu et al. 2022 | Yaffe et al. 2014 | Yang et al. 2018 | Yano et al. 2018 | Yano et al. 2014 | Ylilauri et al. 2017 | Young et al. 2006 |  |
| --- | --- | --- | --- | --- | --- | --- | --- | --- | --- | --- | --- | --- | --- |
|  | **Introduction** | | | | | | | | | | | | |
| Question 1 | | **Y** | **Y** | **Y** | **Y** | **Y** | **Y** | **Y** | **Y** | **Y** | **Y** | **Y** |  |
|  | **Methods** | | | | | | | | | | | | |
| Question 2 | | **Y** | **Y** | **Y** | **Y** | **Y** | **Y** | **Y** | **Y** | **Y** | **Y** | **Y** |  |
| Question 3 | | **N** | **Y** | **N** | **N** | **N** | **N** | **N** | **N** | **N** | **N** | **N** |  |
| Question 4 | | **Y** | **Y** | **Y** | **Y** | **Y** | **Y** | **Y** | **Y** | **Y** | **Y** | **Y** |  |
| Question 5 | | **Y** | **Y** | **Y** | **Y** | **Y** | **Y** | **Y** | **Y** | **Y** | **Y** | **Y** |  |
| Question 6 | | **Y** | **Y** | **Y** | **Y** | **Y** | **Y** | **Y** | **Y** | **Y** | **Y** | **Y** |  |
| Question 7 | | **N** | **N** | **N** | **N** | **N** | **N** | **N** | **N** | **N** | **N** | **N** |  |
| Question 8 | | **Y** | **Y** | **Y** | **Y** | **Y** | **Y** | **Y** | **Y** | **Y** | **Y** | **Y** |  |
| Question 9 | | **Y** | **Y** | **Y** | **Y** | **Y** | **Y** | **Y** | **Y** | **Y** | **Y** | **Y** |  |
| Question 10 | | **Y** | **N** | **Y** | **Y** | **Y** | **Y** | **N** | **Y** | **Y** | **Y** | **Y** |  |
| Question 11 | | **Y** | **Y** | **Y** | **Y** | **Y** | **Y** | **Y** | **Y** | **Y** | **Y** | **Y** |  |
|  | **Results** | | | | | | | | | | | | |
| Question 12 | | **Y** | **Y** | **Y** | **Y** | **Y** | **Y** | **Y** | **Y** | **Y** | **Y** | **Y** |  |
| Question 13 | | **N** | **N** | **N** | **N** | **N** | **N** | **N** | **N** | **N** | **N** | **N** |  |
| Question 14 | | **N** | **N** | **N** | **N** | **N** | **N** | **N** | **N** | **N** | **N** | **N** |  |
| Question 15 | | **Y** | **Y** | **Y** | **Y** | **Y** | **Y** | **Y** | **Y** | **Y** | **Y** | **Y** |  |
| Question 16 | | **Y** | **Y** | **Y** | **Y** | **Y** | **Y** | **Y** | **Y** | **Y** | **Y** | **Y** |  |
|  | **Discussion** | | | | | | | | | | | | |
| Question 17 | | **Y** | **Y** | **Y** | **Y** | **Y** | **Y** | **Y** | **Y** | **Y** | **Y** | **Y** |  |
| Question 18 | | **Y** | **Y** | **Y** | **Y** | **Y** | **Y** | **Y** | **Y** | **Y** | **Y** | **Y** |  |
|  | **Other** | | | | | | | | | | | | |
| Question 19 | | **N** | **N** | **N** | **U** | **N** | **N** | **N** | **N** | **N** | **N** | **N** |  |
| Question 20 | | **Y** | **Y** | **Y** | **Y** | **Y** | **U** | **Y** | **Y** | **Y** | **Y** | **N** |  |

*The colour of the included text is representative of the following: *green* indicates a positive impact on the measure of study quality; *red* indicates a negative impact on the measure of study quality; *orange* indicates an unknown impact on the measure of study quality. N: *no*; U: *unsure*; Y: *yes*.

**Supplemental Table B.5.** List of reference studies used for longitudinal cohort studies.

| Study Cohort | Reference Study |
| --- | --- |
| ARIC | Dearborn-Tomazos et al. (2019) |
| BHS | Carmichael et al. (2019) |
| CARDIA | Suvila et al. (2021) |
| PATH | Cherbuin et al. (2009) |
| WHAP | Szoeke et al. (2019) |
| Whitehall | Brunner et al. (2017) |

**Supplemental Table B.6.** Blood pressure metrics, hypertensive status, and cognitive measures across all included studies.

| Author | Year | TC (mg/dL) | HDL-C (mg/dL) | LDL-C (mg/dL) | Triglycerides (mg/dL) | Dyslipidemia (N =) | Lipid Lowering Medication (N =) | Hypercholesterolemia (N =) | Cognitive Variables  (Mean and SD Available) |
| --- | --- | --- | --- | --- | --- | --- | --- | --- | --- |
| Albanese et al. | **2012** | Total Cohort:  Males = 108 (1938), N = 891  Females = 109.8 (19.8), N = 920 | - | - | - | - | - | - | - |
| Aliberti et al | **2020** | - | - | - | - | Hypertension = 1080  No Hypertension = 576 | - | - | - |
| Alves de Moraes | **2002** | Baseline: Total = 208.2 (37.7) | Baseline (Total): 49.4 (16.4) | Baseline (Total): 132.9 (35.5) | Baseline (Total): 130 (75.4) | - | - | - | *Memory:* Delayed recall: 3.8 (SE = 0.01), 6.7 (SE = 0.01), 6.6 (SE = 0.02), 6.4 (SE = 0.02)  *Working Memory:*  DSST (Total): Normotensive = 48.9 (0.13), Incident Hypertension = 45.5 (0.26), Controlled Hypertension = 43.5 (0.32), Partially Controlled Hypertension = 42.8 (0.33), Uncontrolled Hypertension = 42.4 (0.38) |
| An et al. | **2019** | Q1 (Total): 81.18  Q2 (Total): 81  Q3 (Total): 81.18  Q4 (Total): 84.06 | Q1 (Total): 22.32  Q2 (Total): 22.68  Q3 (Total): 23.4  Q4 (Total): 23.22 | Q1 (Total): 49.86  Q2 (Total): 50.22  Q3 (Total): 48.96  Q4 (Total): 50.76 | Q1 (Total): 27  Q2 (Total): 28.62  Q3 (Total): 25.2  Q4 (Total): 24.48 | - | Q1 (Total): n = 80  Q2 (Total): n = 74  Q3 (Total): n = 25.2  Q4 (Total): n = 24.48 | - | Cholesterol intake quartile, mg/d: Q1(< 188), Q2 (188–283), Q3 (283–385), Q4 (> 385)  *Global Cognition: MoCA*  24 (22, 26), 25 (22, 26), 25 (22, 27), 26 (24, 27)  *Memory*  *AVLT-IR*  14 (11, 18), 14 (11, 18), 15 (12, 18), 15 (12, 19)  *AVLT-SR*  5 (3, 7), 5 (3, 7), 5 (3, 7), 5 (4, 7)  *AVLT-LR*  4 (2, 6), 4 (2, 6), 4 (2, 6), 4 (3, 6)  *LMT*  9.5 (5.5, 14.0), 10.0 (6.0, 14.0), 11.0 (6.5, 15.0), 11.0 (7.5, 15.0)  ***Processing Speed***  *SDMT*  32 (25, 40), 34 (28, 41), 34 (26, 42), 35 (29, 44)  *Attention*  *DSF*  8 (7, 8), 8 (7, 9), 8 (7, 9), 8 (7, 9) |
| Babaei et al. | **2013** | HC = 147.42 (42.99), N = 10  MC = 203.14 (35.31), N = 10  HE = 168.87 (24.07), N = 11  ME = 252.5 (178.73), N = 11 | HC = 44.71 (11.16), N = 10  MC = 37.28 (1.79), N = 10  HE = 46.5 (5.63), N = 11  ME =37.5 (3.33), N = 11 | HC = 115.28 (25.11), N = 10  MC = 163.28 (10.43), N = 10  HE = 119.25 (33.8), N = 11  ME =133.25 (46.53), N = 11 | - | - | - | - | - |
| Backestrom et al. | **2015** | Male = 106.2 (23.04), N = 127  Female = 106.2 (23.22), N = 164  Total = 106.2 (22.32), N = 291 | - | - | - | - | - | - | *Episodic memory:* Summary Score: Males = 6.62 (1.3), Females = 7.29, Total = 7 (1.39)  *Semantic Memory Score:* Males (Total): 15.2 (3.03), 40 Years: 15.56 (2.07), 50 Years: 15.67 (2.9), 60 Years: 14.33 (3.72) Females (Total): 15.96 (2.82), 40 Years: 16.86 (2.1), 50 Years: 15.86 (3.07), 60 Years: 15.39 (2.93) Total: 15.6 (2.94), 40 Years: 16.27 (2.17), 50 Years: 15.77 (2.98), 60 Years: 14.96 (3.3) |
| Boots et al. | **2015** | Wave 2 Total Participants = 200.3 (35.21), N = 315 | Wave 2 Total Participants: 62.39 (18.8), N = 315 | - | - | - | - | - | *Global Cognition* MMSE (Total): 29.47 (0.88) IQCODE (Total): 43.38 (3.01) |
| Bressler et al. | **2013** | Total Whites = 97.2 (18), N = 8353 Total African American = 97.2 (18), N = 2062 | Total Whites = 23.4 (7.2), N = 8328 Total African American = 25.2 (7.2), N = 2055 | Total Whites = 61.2 (16.2), N = 8212 Total African American = 63 (18)), N = 2046 | Total Whites = 28.8 (16.2), N = 8351 Total African American = 21.6 (12.6), N = 2062 | - | - | Total Whites = 4132 Total African American = 1067 | *Verbal Memory:* Delayed Recall (Total) White (rs9939609): TT: 6.8 (1.4); AT: 6.8 (1.4); AA: 6.9 (1.4) White ( rs17817449): TT: 6.8 (1.4); GT: 6.8 (1.4); GG: 6.9 (1.4) White (rs805136): CC: 6.8 (1.4); AC: 6.8 (1.4); AA: 6.9 (1.4) White ( rs1421085): TT: 6.8 (1.4); CT: 6.8 (1.4) ; CC:6.9 (1.4) African American (rs9939609): TT: 6.3 (1.5); AT: 6.3 (1.6); AA: 6.4 (1.6) African American ( rs17817449): TT: 6.3 (1.6); GT: 6.3 (1.6); GG: 6.4 (1.6) African American (rs805136): CC: 6.3 (1.5); AC: 6.3 (1.6); AA: 6.3 (1.6) African American ( rs1421085): TT: 6.4 (1.6); CT: 6.2 (1.6) ; CC:6.3 (1.4)  *Working Memory* DSST (Total) White (rs9939609): TT: 49.5 (11.2); AT: 49.9 (11.2); AA: 49.9 (11.3) White ( rs17817449): TT: 49.5 (11.2); GT: 49.9 (11.2); GG: 49.9 (11.4) White (rs805136): CC: 49.4 (11.1); AC: 49.9 (11.2); AA: 49.9 (11.4) White ( rs1421085): TT: 49.5 (11.2); CT: 49.9 (11.2) ; CC:49.9 (11.3) African American (rs9939609): TT: 33.6 (13.1); AT: 32.6 (13.1); AA: 33.8 (13) African American ( rs17817449): TT: 33.1 (13.2); GT: 32.7 (13.0); GG: 34.3 (13.2) African American (rs805136): CC: 33.0 (13.3); AC: 32.9 (13.1); AA: 34.1 (13.2) African American ( rs1421085): TT: 33.2 (13.2); CT: 32.8 (13.0); CC: 33.6 (11.9) |
| Britton et al. | **2004** | Phase 5 Data Collection: Males = 106.2, N = 4272  Females = 108, N = 1761 | - | - | - | - | - | - | *Working Memory* Composite Score: Males = 6.85 (2.33), Females = 6.87 (2.72)  *Inductive Reasoning* AH-4: Male = 48.61 (10.07), Female = 41.21 (12.41) |
| Carmichael et al. | **2019** | Total: 186.6 (34.3) | - | - | - | - | - | - | Memory:  Logical memory  This study = 17.2 (6.9), Recent cognitive assessment = 16.0 (7.3)  Executive function:  Digit Coding  This study = 65.6 (16.4)  Recent cognitive assessment = 58.8 (17.7)  DSBT  This study = 7.7 (2.2)  Recent cognitive assessment = 7.7 (2.5)  TMT-B  This study = 57.4 (28.8)  Recent cognitive assessment = 62.3 (30.1) |
| Chen et al. | **2015** | Baseline: Females = 104.04 (17.1), N= 247 | Baseline: Females = 28.08 (7.56), N = 247 | Baseline (Females) = 67.5 (16.38), N = 247 | - | - | - | - | *Psychomotor Speed* SDMT: Female: Normotensive = 52.8, Pre-hypertensive = 50, Hypertensive = 47.1 |
| Cherbuin et al. | **2009** | - | - | - | - | - | Normal at Wave 2 = 467  MCI at Wave 2 = 4  Any MCD at Wave 2 = 25 | - | *Global Cognition* MMSE (Total): 29.37 (0.895) |
| Chosy et al. | **2019** | SCI Present = 100.8 (16.2), N = 189  SCI Absent = 102.6 (16.2), N = 3393 | - | - | - | - | - | - | - |
| Christman et al. | **2011** | Total = 97.2 (18), N = 8958  Non-diagnosed Diabetes  HbA1c <5.7% = 97.2 (18), N = 6589  HbA1c 5.7-6.5% = 99 (18), N = 1542  HbA1c ≥6.5% = 99 (18), N = 311  Diagnosed Diabetes  HbA1c <7.0% = 95.4 (18), N = 144  HbA1c 7.0-8.0% = 95.4 (18), N = 107  HbA1c ≥8.0% = 100.8 (19.8), N = 265 | Total = 23.4 (7.2), N = 8958  Non-diagnosed Diabetes  HbA1c <5.7% = 23.4 (7.2), N = 6589  HbA1c 5.7-6.5% = 21.6 (7.2), N = 1542  HbA1c ≥6.5% = 19.8 (5.4), N = 311  Diagnosed Diabetes  HbA1c <7.0% = 19.8 (7.2), N = 144  HbA1c 7.0-8.0% = 19.8 (5.4), N = 107  HbA1c ≥8.0% = 19.8 (7.2), N = 265 | Total = 61.2 (16.2), N = 8958  Non-diagnosed Diabetes  HbA1c <5.7% = 61.2 (16.2), N = 6589  HbA1c 5.7-6.5% = 64.8 (16.2), N = 1542  HbA1c ≥6.5% = 64.8 (18), N = 311  Diagnosed Diabetes  HbA1c <7.0% = 61.2 (18), N = 144  HbA1c 7.0-8.0% = 61.2 (16.2), N = 107  HbA1c ≥8.0% = 66.6 (18), N = 265 | Total = 25.2 (12.6), N = 8958  Non-diagnosed Diabetes  HbA1c <5.7% = 25.2 (12.6), N = 6589  HbA1c 5.7-6.5% = 27 (12.6), N = 1542  HbA1c ≥6.5% = 30.6 (14.4), N = 311  Diagnosed Diabetes  HbA1c <7.0% = 30.6 (14.4), N = 144  HbA1c 7.0-8.0% = 32.4 (14.4), N = 107  HbA1c ≥8.0% = 34.2 (16.2), N = 265 | - | - | - | *Verbal Memory* Delayed Recall: Total = 6.7 (1.5)  *Working Memory* DSST (Total): 47.4 (12.8) |
| Chuang et al. | **2023** | Males = 200.7 (35.7), n = 298  Females = 205.6 (39.8), n = 320 | Males = 47.2 (12.1), n = 298  Females = 56.3 (1.4), n = 320 | Males = 125.9 (32.6), n = 298  Females = 124.0 (33.1), n = 320 | Males = 139.3 (99.5), n = 298  Females = 129.4 (103.7), n = 320 | Males: n = 132  Females: n = 136 | - | - | Global Cognition  MMSE: Male = 28.5 (1.9), Female = 27.9 (2.8) |
| Cohen-Manheim et al. | **2016** | Total = 79.2 (14.4), N = 507 | Total = 18 (5.4), N = 507 | Total = 48.6 (12.6), N = 507 | Total = 25.2 (16.2), N = 507 | - | - | - | - |
| Creavin et al. | **2012** | No TC Data Available | Phase 1 (Total): 23.4, N = 2512 Phase 2 (Total): 18, N = 2398 Phase 3 (Total): 28.8, N = 2046 | - | Phase 1 (Total): 27, N = 2512 Phase 2 (Total): 28.8, N = 2398 Phase 3 (Total): 26.8, N = 2046 | - | - | - | *Inductive Reasoning* AH-4 (Total): 73.1  *Global Cognition* MMSE (Total): 27 CAMCOG (Total): 90 NART (Total): 28 |
| Cui et al. | **2016** | Total: Hypertension = 81.9 (12.06), N = 278 Normotensive = 79.56 (10.62), N = 155 | Total: Hypertension = 25.2 (6.48), N = 278 Normotensive = 25.38 (5.58), N = 155 | Total: Hypertension = 47.16 (15.84), N = 278 Normotensive = 47.16 (8.82), N = 155 | Total: Hypertension = 29.7 (14.58), N = 278 Normotensive = 27.54 (13.14), N = 155 | - | - | - | *Intelligence* WAIS (Total): Control (Normotensive): 99.23 (8.64), Non-Treated Hypertension: 98.77 (9.46), Treated Hypertension: 104.53 (6.68), Compliant Hypertension: 105.31 (9.02), Non-Compliant Hypertension: 102.77 (9.85)  *Working Memory* CMS Score (Total): Control (Normotensive): 84.11 (9.96), Non-Treated Hypertension: 70.02 (16.53), Treated Hypertension: 74.91 (14.23), Compliant Hypertension: 81.86 (11.85), Non-Compliant Hypertension: 65.12 (12.43)  *Global Cognition* MMSE (Total): Control (Normotensive): 27.59 (2.25), Non-Treated Hypertension: 26.98 (3.04), Treated Hypertension: 728.91 (1.06), Compliant Hypertension: 29.04 (1.62), Non-Compliant Hypertension: 27.65 (2.18) |
| de Menezes et al. | **2021** | Total (Baseline) = 70.2 (18), N = 7063 | - | - | - | - | Total (Baseline) = 1399 | - | *Working Memory* DSST (Total): 36.8 (6.1)  *Verbal Fluency*  Word Fluency Test (Total(: 29.8 (8.4) |
| Dearborn et al. | **2014** | - | No MetS: Males = 48.3 (12.7), Females = 64.1 (16.1 MetS: Males = 36.9 (9.6), Females = 48 (13.6) | - | No MetS: Males = 106.2 (53.1), Females = 97.3 (44.2) MetS: Males = 194.7 (115), Females = 168.1 (97.3) | - | No MetS: Males = 32, Females = 15 MetS: Males = 105, Females = 140 | - | *Verbal Memory* Immediate Recall:  MetS: Males = 6.5 (1.5), Females = 7.1 (1.4) No MetS: Males = 6.3 (1.4), Females = 6.8 (1.4)  *Working Memory* DSST: MetS: Males = 44.5 (12.7), Females = 50.4 (12.9) No MetS: Males = 43.9 (11.9), Females = 45 (14.1) |
| Dearborn-Tomazos et al. | **2019** | Total = 214 (41.7), N = 13,588 | - | - | - | - | - | - | - |
| Debette et al. | **2011** | - | - | - | - | - | - | Examination 5 (Total) = 243 | *Verbal Memory* Delayed Recall (Total): 10.83 (3.5)  *Visuospatial Organisation*  VR-d Test (Total): 8.62 (3.24) |
| Del Vecchio et al. | **2023** | - | - | - | - | - | - | Total: n = 12  Control Group: n = 1  Study Group: n = 10 | MMSE  Total: 25.8 (2.2); Control: 26.5 (2.3); Study: 25.6 (2.2)  MoCA  Total: 25.2 (2.7); Control: 26.5 (2.9); Study: 24.6 (2.4) |
| Derby et al. | **2021** | - | - | - | - | - | - | Total: n = 528 | SDMT: 58.8 (10.3)  High TG’s (Yes, No): 58.9 (10.5), 58.6 (9.8)  Low HLD-C (Yes, No): 58.9 (10.4), 58.5 (10.1)  Hyperlipidaemia (Yes, No): 59.3 (10.1), 58.2 (10.6)  DSBT: 7.0 (2.3)  High TG’s (Yes, No): 7.0 (2.4), 6.9 (2.2)  Low HLD-C (Yes, No): 7.0 (2.4), 6.9 (2.2)  Hyperlipidaemia (Yes, No): 7.0 (2.3), 6.9 (2.4)  EBMT: 10 (10, 12)  High TG’s (Yes, No): 1 (566), 2 (583)  Low HLD-C (Yes, No): 1 (553), 2 (617)  Hyperlipidaemia (Yes, No): 1 (572), 2 (568) |
| Elbaz et al. | **2014** | Follow-up 1 (Total) = 114.84 (20.34), N = 4699 | Follow-up 1 (Total) = 26.1 (7.38), N = 4699 | - | - | - | - | - | *Inductive Reasoning* AH-4 (Total): 47 (10) |
| Elkins et al. | **2005** | - | Baseline (Visit 1 - Total) = 50 (17), N = 12,096 | Baseline (Visit 1 - Total) = 133 (37), N = 12,096 | - | - | - | - | *Verbal Memory* Delayed Recall (Total): 7 (1.5)  *Working Memory* DSST (Total): 45 (14) |
| Fava et al. | **2013** | - | - | - | Group A (Total): 115 (44)  Group B (Total): 121 (52) | - | - | - | reynolds |
| Ferguson et al. | **2018** | Total = 192.3 (35.2), N = 634 | - | - | - | - | - | - | - |
| Fuh et al. | **2007** | Normal: 90 (18)  IGT: 91.8 (18)  DM:100.8 (18) | - | - | Normal: 15.3  IGT: 19.8  DM:29.34 | - | - | - | RAVLT  Normal: 9.9 (2.5)  Impaired Glucose Control:10.6 (2.2)  Diabetes: 9.8 (2.8)  Continuous Recognition Paradigm of Kimura  Normal: 69.4 (5.7)  Impaired Glucose Control: 69.9 (5.5)  Diabetes: 68.2 (6.4)  VFT  Normal: 13.6 (3.9)  Impaired Glucose Control: 14.5 (4.0)  Diabetes: 13.7 (4.1)  TMT-A  Normal: 83.7 (46.2)  Impaired Glucose Control: 74.8 (30.8)  Diabetes: 91.4 (43.1)  TMT-B  Normal: 116.1 (43.2)  Impaired Glucose Control: 129.0 (60.9)  Diabetes: 135.8 (62.7)  DSFT  Normal: 9.2 (2.5)  Impaired Glucose Control: 9.4 (2.6)  Diabetes: 7.9 (2.7)  DSBT  Normal: 2.9 (1.6)  Impaired Glucose Control:3.3 (1.9)  Diabetes: 2.7 (1.8) |
| Gerber et al. | **2021** | - | - | - | - | - | - | Total: n = 668 | DSST  Overall: 70.0 (16.0)  Liver attenuation: No NAFLD >51 HU: 70.6 (16.3)  Mild NAFLD >40–51 HU: 67.6 (15.6)  Severe NAFLD ≤40 HU: 68.9 (14.4)  RAVLT  Overall: 8.3 (3.3)  Liver attenuation: No NAFLD >51 HU: 8.4 (3.3)  Mild NAFLD >40–51 HU: 7.9 (3.1)  Severe NAFLD ≤40 HU:8.0 (3.1)  Stroop  Overall: 22.9 (10.8)  Liver attenuation: No NAFLD >51 HU: 22.7 (10.7)  Mild NAFLD >40–51 HU: 24.3 (11.6)  Severe NAFLD ≤40 HU:22.9 (10.2) |
| Giugliano et al. | **2018** | Control (Baseline - Total): 198.2 (23.7), N = 18 Active Treatment (Baseline - Total): 201.9 (27.5), N = 18 | Control (Baseline - Total): 47.6 (7.9), N = 18 Active Treatment (Baseline - Total): 51.3 (7.2), N = 18 | Control (Baseline - Total): 129.2 (26.1), N = 18 Active Treatment (Baseline - Total): 126.7 (30.4), N = 18 | Control (Baseline - Total): 107.1 (34.8), N = 18 Active Treatment (Baseline - Total): 119.5 (27.9), N = 18 | - | - | - | - |
| Gonzalez et al. | **2018** | Total = 214.99 (41.67), N = 13,270  White = 214.77 (40.47), N = 2998  Black = 215.73 (45.53), N = 10,272 | - | - | - | - | Total = 380  White = 339  Black =41 | - | - |
| Gottesman et al. | **2017** | Visit 1 Total:  <200 = 37.5%  200 to <240 = 37.5%  ≥240 = 25% | - | - | - | - | - | - | - |
| Gourley et al. | **2020** | Total = 199 (39), N = 132 | Total: 53 (17), N = 132 | - | -- | - | - | - | - |
| Hajjar et al. | **2018** | - | - | - | - | - | - | Total = 96 | *Working Memory* DSST (Total): 55 (0.4) |
| Hakamada-Taguchi et al. | **2002** | Total = 106.2 (14.4), N = 26 | - | - | - | - | - | - | *Working Memory* DSST (Total): 67.4 (1.07) |
| Haley et al. | **2010** | - | Control (Total): 56.8 (14.7), N = 25 Metabolic Syndrome (Total) = 36.5 (10), N = 13 | Control (Total): 125.2 (32.1), N = 25 Metabolic Syndrome (Total) = 129.3 (32.5), N = 13 | Control (Total): 5116.8 (66.7), N = 25 Metabolic Syndrome (Total) = 268.2 (97.5), N = 13 | - | - | - | *Intelligence* WAIS (Total): 112.6 (11.5) |
| Henderson et al. | **2003** | Females  Years 1+2: 104.94 (16.2)  Years 4+5: 104.94 (16.02)  Years 7+8:105.12 (16.38) | Females  Years 1+2: 28.44 (7.38)  Years 4+5: 29.16 (7.2)  Years 7+8:26.82 (7.02) | Females  Years 1+2: 67.86 (16.02)  Years 4+5: 65.52 (15.84)  Years 7+8:68.22 (15.66) | Females  Years 1+2: 19.26 (12.96)  Years 4+5: 23.04 (16.56)  Years 7+8:23.4 (14.22) | - | Females  Year 8: n = 29 | Total: n = 668 | - |
| Henriksen et al. | **2017** | Group A (No Decline) = 99, N = 95  Group B (Decline) = 99, N = 94 | No HDL-C Data Available*  (HDL:LDL ratio provided) | - | - | - | - | - | *Global Cognition* MMSE (Total): No Decline = 30, Decline = 29 BPP (Total): No Decline = 46, Decline = 48 IST (Total): No Decline = 21, Decline = 44 ACE (Total): No Decline = 93, Decline = 97 |
| Hossain et al. | **2020** | - | - | - | - | Males = 33  Females = 66 | - | - | - |
| Houle et al. | **2019** | - | - | - | - | Males = 861  Females = 1000  Total = 1861 | - | - | *Global Cognition* HRS Composite Score: Males = 14.2 (4.15), Females = 14.44 (3.96), Total = 14.31 (4.06) |
| Ihle-Hansen et al. | **2019** | - | - | - | - | - | Males = 523  Females = 396  Total = 892 | Males = 894  Females = 902  Total =1796 | *Global Cognition*  MoCA: Males = 25 (2.9), Females = 25.5 (2.9), Total = 25.3 (2.9) |
| Kaffashian et al. | **2013** | Comparison 1 Framingham CVD Risk Score (Total): 229.7 (40.4), N = 4374 | Comparison 1 Framingham CVD Risk Score (Total): 56.7 (15.3), N = 4374 | - | - | - | - | - | - |
| Kaffashian et al. | **2011** | Total Cohort:  Males = 227.5 (39.1), N = 3353  Females = 230.9 (41.3), N = 1295 | Total Cohort:  Males = 53 (13.2), N = 3353  Females = 65 (16.6), N = 1295 | - | - | - | - | - | - |
| Kalmijn et al. | **2002** | Total Cohort:  Males = 104.4 (18.18), N = 905  Females = 104.58 (18.9), N = 989 | - | - | - | - | - | - | *Verbal Memory* Delayed Word Recall (Total): Never Smokers = 8.2, Former Smokers = 7.9, Current Smokers = 7.9 |
| Kivipelto et al. | **2001** | Without MCI: 52.4 | - | - | - | - | - | - | MMSE  With MCI: 22.6 (0.4)  Without MCI:26.3 (1.9) |
| Knopman et al. | **2001** | - | - | - | - | -- | - | Females (Total) = 4533 | *Verbal Memory:* Delayed Recall (Total): 6.94 (1.4)  *Working Memory* DSST (Total): 48.62 (13.5) |
| Knopman et al. | **2018** | Total (Normal):  <200 = 43%  200 to <240 = 38%  ≥240 = 19% | - | - | - | - | - | Females (Total) = 4533 | - |
| Kovacs et al. | **2014** | Healthy controls (free of hypertension or hyperlipidemia - Total): 85.86 (1.62), N = 44 Normotensive subjects with elevated LDL–C levels (Total): 111.24 (1.98), N = 41 Hypertensive patients with normal LDL–C levels (Total): 86.04 (1.98), N = 49 Hypertensive patients with elevated LDL–C levels (Total): 114.66 (1.98), N = 23 | Healthy controls (free of hypertension or hyperlipidemia - Total): 29.16 (1.26), N = 44 Normotensive subjects with elevated LDL–C levels (Total): 26.64 (1.26), N = 41 Hypertensive patients with normal LDL–C levels (Total): 26.64 (1.62), N = 49 Hypertensive patients with elevated LDL–C levels (Total): 28.08 (1.8), N = 23 | Healthy controls (free of hypertension or hyperlipidemia - Total): 48.24 (1.26), N = 44 Normotensive subjects with elevated LDL–C levels (Total): 73.62 (1.42), N = 41 Hypertensive patients with normal LDL–C levels (Total): 48.42 (1.44), N = 49 Hypertensive patients with elevated LDL–C levels (Total): 75.24 (1.8), N = 23 | Healthy controls (free of hypertension or hyperlipidemia - Total): 18.72 (1.62), N = 44 Normotensive subjects with elevated LDL–C levels (Total): 27.36 (1.98), N = 41 Hypertensive patients with normal LDL–C levels (Total): 24.84 (2.34), N = 49 Hypertensive patients with elevated LDL–C levels (Total): 28.62 (3.06), N = 23 | - | - | Normotensive subjects with elevated LDL–C levels (Total): 41 Hypertensive patients with elevated LDL–C levels (Total): 23 | *Verbal Memory* RAVLT (Summary Metric) Healthy controls (free of hypertension or hyperlipidemia - Total): 13.19 (SE = 0.38) Normotensive subjects with elevated LDL–C levels (Total): 13.05 (SE = 0.42) Hypertensive patients with normal LDL–C levels (Total): 12.28 (SE = 0.44) Hypertensive patients with elevated LDL–C levels (Total): 11.48 (SE = 0.86)  *Attention* TMT-A Healthy controls (free of hypertension or hyperlipidemia - Total): 32.25 (2.11) Normotensive subjects with elevated LDL–C levels (Total): 33.11 (2.49) Hypertensive patients with normal LDL–C levels (Total): 27.51 (1.67) Hypertensive patients with elevated LDL–C levels (Total): 35.95 (3)  Simple/Selective Reaction Time Healthy controls (free of hypertension or hyperlipidemia - Total): 0.62 (0.01) Normotensive subjects with elevated LDL–C levels (Total): 0.65 (0.01) Hypertensive patients with normal LDL–C levels (Total): 0.65 (0.02) Hypertensive patients with elevated LDL–C levels (Total): 0.64 (0.03)  Choice Reaction Time Healthy controls (free of hypertension or hyperlipidemia - Total): 0.52 (0.01) Normotensive subjects with elevated LDL–C levels (Total): 0.54 (0.01) Hypertensive patients with normal LDL–C levels (Total): 0.56 (0.01) Hypertensive patients with elevated LDL–C levels (Total): 0.56 (0.02)  *Working Memory* Digit Span Backwards  Healthy controls (free of hypertension or hyperlipidemia - Total): 11.7 (0.31) Normotensive subjects with elevated LDL–C levels (Total): 11.97 (0.42) Hypertensive patients with normal LDL–C levels (Total): 10.07 (0.3) Hypertensive patients with elevated LDL–C levels (Total): 10 (0.54)  *Psychomotor Speed* SDMT (Total) Healthy controls (free of hypertension or hyperlipidemia - Total): 52.77 (1.5) Normotensive subjects with elevated LDL–C levels (Total): 49.63 (1.43) Hypertensive patients with normal LDL–C levels (Total): 50.6 (1.34) Hypertensive patients with elevated LDL–C levels (Total): 44.33 (2.69)  *Visuospatial Organisation* Block Design Test Healthy controls (free of hypertension or hyperlipidemia - Total): 26.07 (0.53) Normotensive subjects with elevated LDL–C levels (Total): 25.28 (0.53) Hypertensive patients with normal LDL–C levels (Total): 25.67 (0.58) Hypertensive patients with elevated LDL–C levels (Total): 24.9 (0.83) |
| Kumar et al. | **2008** | Diabetes: 77.94 (17.1)  Non-Diabetics: 89.1 (17.82) | - | - | - | - | - | - | Diabetics; Non-Diabetics  MMSE: 29.03 (1.65) 29.40 (0.92)  Immediate recall: 6.95 (2.29) 7.53 (2.05)  Delayed recall: 6.18 (2.24) 6.67 (2.24)  Purdue Pegboard-both hands: 9.87 (1.36) 10.66 (1.62)  Purdue Pegboard-dominant hand: 12.31 (1.38) 13.59 (1.95)  Purdue Pegboard-nondominant hand: 12.33 (1.72) 13.06 (1.85)  Mean RT (choice): 0.32 (0.03) 0.32 (0.04)  Mean RT (simple): 0.26 (0.03) 0.25 (0.05)  SDMT: 47.97 (7,94) 51.48 (8.56)  Spot-the-Word test: 51.23 (6.53) 52.49 (5.70) |
| Kumar et al., | **2020** | - | - | - | - | - | - | Total:  White = 18  African American = 29 | *Attention* TMT-A (Total): White = 70, African American = 81 Digit Span Forward (Total): White = 7, African American = 6.5  *Intelligence*  Mental Rotation (Total): White = 18.5, African American = 17.5  *Working Memory* Digit Span Backwards: Males = 5, Females = 4  *Global Cognition* MoCA (Total): White = 27, African American = 25 MINT (Total): White = 31, African American = 29 |
| Kumari et al., | **2005** | - | NGT: Male = 24.12, N = 3407; Female = 29.34, N = 1334 IGT: Male = 23.4, N = 405; Female = 27.72, N = 192 Diabetes: Male = 22.14, N = 208; Female = 25.2, N = 101 | - | NGT: Male = 21.42, Female = 17.46 IGT: Male = 25.02, Female = 21.96 Diabetes: Male = 32.22, Female = 25.38 | - | - | - | *Verbal Memory* Delayed Recall (Total) Male: NGT = 6.87 (1), IGT = 6.72 (1.03), Diabetes = 6.87 (1.2) Female: NGT = 6.96 (1), IGT = 6.75 (0.95), Diabetes = 6.24 (1.39)  *Inductive Reasoning* AH-4 Male: NGT = 48.7, IGT = 49.49, Diabetes = 47.26 Female: NGT = 41.79, IGT = 41.61, Diabetes = 39.73 |
| Lin et al. | **2020** | - | - | - | - | - | - | Total = 103 | *Attention* Attention Index (MoCA - Total) = 16.28 (1.78)  *Working Memory* Memory Index Score (MoCA - Total): 12.72 (2.38)  *Temporal Orientation* Orientation Index Score (MoCA - Total): 5.84 (0.43)  *Visuospatial Organisation* Visual Index Score (MoCA - Total): 6.48 (0.92)  *Global Cognition* MoCA (Total): 26.1 (2.89) |
| Liu et al. | **2022** | - | Controls: 21.6 (3.6)  SCI: 19.8 (5.4) | Controls: 43.2 (14.4)  SCI:45 (16.2) | - | - | - | - | MoCA: Control = 27.4 (1.3), SCI: 23.3 (2.5) |
| Lopez-Oloriz et al. | **2014** | - | - | - | - | Total = 54 | - | - | - |
| Lutski et al. | **2019** | Total:  ≥200 = 142 (42.1)  <200 = 195 (57.9) | Total:  <30 = 60 (17.8)  ≥30 = 277 (82.2) | Total:  ≥160 = 97 (28.8)  <160 = 240 (71.2) | Total:  ≥200 = 60 (17.8)  <200 = 277 (82.2) | - | - | - | - |
| Mahinrad et al. | **2020** | Total:  Hypertensive (Year 0) = 183 (38). N = 70  Normotensive (Year 0) = 175 (30), N = 121 | - | - | - | - | - | - | *Verbal Memory* Delayed Word Recall (Total): 10  *Attention* TMT-A (Total) = 28  *Working Memory* DSST (Total): 68 (16) Digit Span Backwards (Total): 18 (4) |
| Masi et al. | **2018** | Total = 102.42 (21.6), N = 1186 | Total = 29.16 (7.38), N = 1186 | Total = 63.36 (18), N = 1156 | Total = 19.8, N = 1162 | - | - | - | *Attention* Selective/Simple Reaction Time (Total): 281 (63.5) Choice Reaction Time (Total): 612 (76.5) |
| Mefford et al. | **2021** | LDL-C <100: 162.1 (27)  LDL-C 100-129: 184.3 (26.8)  LDL-C 130-159: 197.7 (32.6)  LDL-C ≥160: 216.9 (41) | LDL-C <100: 62.6 (21.5)  LDL-C 100-129: 58.9 (18)  LDL-C 130-159: 56.8 (16.5)  LDL-C ≥160: 54.8 (15.9) | - | LDL-C <100: 94.2 (70.7)  LDL-C 100-129: 110 (88.5)  LDL-C 130-159: 115.4 (71.8)  LDL-C ≥160: 132.3 (106.4) | - | LDL-C <100: 16  LDL-C 100-129: 79  LDL-C 130-159: 176  LDL-C ≥160: 235 | - | DSST  <100 = 70.6 (16.8)  100–129 = 71.1 (16.6)  130–159 = 70.4 (15.6)  ≥160 = 68.0 (15.1)  RAVLT  <100 = 9.0 (2.0)  100–129 = 9.1 (1.9)  130–159 = 9.0 (1.9)  ≥160 = 8.7 (1.9)  Stroop Test  <100 = 22.6 (11.8)  100–129 = 22.2 (10.5)  130–159 = 22.4 (9.8)  ≥160 = 24.0 (12.1) |
| Moore et al. | **2014** | - | - | - | - | - | - | Total = 145 | *Intelligence* AFQT (Total): 61.8 (0.95) |
| Muhkerjee et al. | **2022** | Diabetic Retinopathy: 207.1 (61.5)  W/O Diabetic Retinopathy: 192.8 (51.6) | - | - | - | - | - | - | *MMSE score*  Diabetic retinopathy: 23.5 (3.3)  Without diabetic retinopathy: 25.3 (3.3)  *Orientation domain score*  Diabetic retinopathy: 8.2 (1.3)  Without diabetic retinopathy: 9.0 (1.1)  *Registration domain score*  Diabetic retinopathy: 2.8 (0.4)  Without diabetic retinopathy: 3.0 (0.2)  *Attention/calculation domain score*  Diabetic retinopathy: 3.7 (1.1)  Without diabetic retinopathy: 4.1 (1.3)  *Recall domain score*  Diabetic retinopathy: 2.1 (0.8)  Without diabetic retinopathy: 2.7 (0.5)  *Language domain score*  Diabetic retinopathy: 6.6 (1.2)  Without diabetic retinopathy: 6.6 (1.2) |
| Mukerji et al. | **2016** | Total: 197.2 (4.04) | Total: 47.6 (13.3) | Total: 115.4 (34.8) | Total: 161 (123.1) | - | Total: n = 304 | - | - |
| Nunley et al. | **2017** | Total: 174.79 (34.92) | Total: 60.63 (16.68) | Total: 98.15 (28.44) | - | - | Total: n = 57 | - | Statin Use: Never used (n = 51); 1-6 yr (n = 25); 7-12 yr (n = 32)  Estimated verbal IQ 108.6 ± 8.2 107.7 ± 10.0 106.5 ± 6.9 0.24  Memory domain z-score 0.24 ± 0.75, -0.23 ± 0.64, -0.25 ± 0.78  Executive function z-score 0.18 ± 0.56, -0.10 ± 0.82, -0.30 ± 0.79  Psychomotor speed z-score 0.29 ± 0.66, -0.33 ± 1.10, -0.28 ± 0.89  Visuo- construction z-score 0.21 ± 0.64, -0.16 ± 0.82, -0.21 ± 1.45 |
| Palacios-Mendoza et al. | **2018** | Diabetes (Total) = 207.16 (41.33), N = 142 No Diabetes (Total) = 229 (38.38), N = 167 | Diabetes (Total) = 42.18 (8.96), N = 142 No Diabetes (Total) = 47.9 (10.9), N = 167 | Diabetes (Total) = 133.66 (36.19), N = 142 No Diabetes (Total) = 157.64 (34.43), N = 167 | Diabetes (Total) = 202.91 (123.23), N = 142 No Diabetes (Total) = 169.19 (88.93), N = 167 | - | Diabetes (Total) = 13 No Diabetes (Total) = 12 | - | *Verbal Memory* RAVLT (Immediate Recall - Total): Diabetes = 6.57 (2.57), No Diabetes = 7.5 (2.68) RAVLT (Delayed Recall - Total): Diabetes = 6.35 (2.89), No Diabetes = 7.67 (2.69) RAVLT (Learning Score - Total): Diabetes = 34.38 (8.39), No Diabetes = 38.78 (8.3) ROCF (Immediate Recall - Total): Diabetes = 14.53 (7.42), No Diabetes = 17.76 (7.77) ROCF (Delayed Recall - Total): Diabetes = 13.28 (7.82), No Diabetes = 16.23 (7.82)  *Attention* TMT-A (Total): Diabetes = 72.92 (30.57), No Diabetes = 61.42 (30.04) Choice Reaction Time (Total): Diabetes = 1954.57 (658.59), No Diabetes = 1769.19 (580.65) Digit Span Forward Test (Total): Diabetes = 6.51 (1.72), No Diabetes = 7.1 (1.96)  *Working Memory* Digit Span Backwards (Total): Diabetes = 3.88 (1.73), No Diabetes = 4.41 (1.97) |
| Panigrahi et al. | **2021** | - | - | - | - | Total = 54 | - | - | *Global Cognition:* MMSE score: 25.37 ± 3.34 |
| Pearson et al. | **2017** | - | Male: 21.6  Female: 25.2 | - | Male: 28.8  Female:23.4 | - | - | - | Global Cognition: 27.1 |
| Power et al., | **2017** | Total = 210 (39.5), N = 13,997 | Total = 49.5 (16.7), N = 13,997 | Total = 133.6 (36.8), N = 13,997 | Total = 136.1 (90.3), N = 13,997 | - | Total = 743 | - | *Verbal Memory* Delayed Recall: Male = 6.3 (1.5), Female = 6.8 (1.5)  *Working Memory* DSST (Total): Hypertension = 42.4 (13.4), Normotensive = 46.4 (14.6) |
| Rask et al. | **2016** | Neg. Cognitive Change: 95.4 (15.3)  Pos. Cognitive Change: 99.72 (16.38) | - | - | - | - | - | - | - |
| Ravona-Springer et al. | **2020** | FH+: 197.72 (27.07)  FH-: 193.59 (27.72) | FH+: 52.91 (12.26)  FH-: 53.94 (13.05) | FH+: 119.96 (23.17)  FH-: 114.18 (27.11) | FH+: 121.62 (52.49)  FH-: 120.97 (62.85) | - | - | FH+: 123  FH-: 32 | - |
| Rawlings et al., | **2014** | Total = 210 (39.5), N = 13,351  Diabetes (Total) = 216 (45.5), N = 1779  No Diabetes (Total) = 209 (38.4), N = 11,572 | Total = 49.4 (16.7), N = 13,351  Diabetes (Total) = 43.1 (14.2), N = 1779  No Diabetes (Total) = 50.4 (16.8), N = 11,572 | - | Total = 136 (90.3), N = 13,351  Diabetes (Total) = 178 (135.3), N = 1779  No Diabetes (Total) = 130 (79.4), N = 11,572 | - | - | - | - |
| Razavi et al., | **2020** | No TC Data Available | Total = 51.7 (16.1), N = 960 | Total = 115 (35.6), N = 960 | Total = 106, N = 960 | - | - | - | - |
| Reis et al. | **2013** | - | Coronary artery calcified plaque  Present: 53.9 (17.6)  Absent: 59.4 (17.6)  Abdominal aortic calcified plaque  Present: 55.5 (16.9)  Absent: 60.5 (18.4) | Coronary artery calcified plaque  Present: 116.4 (35.5)  Absent: 111.9 (31.1)  Abdominal aortic calcified plaque  Present: 115.9 (34.4)  Absent: 110.2 (29.9) | Coronary artery calcified plaque  Present: 108  Absent: 100  Abdominal aortic calcified plaque  Present: 88  Absent: 86 | Coronary artery calcified plaque  Present: n = 279  Absent: n = 575  Abdominal aortic calcified plaque  Present: n = 511  Absent: n = 343 | - | - | - |
| Reynolds et al. | **2011** | Total: 119.16 (23.94)  Male: 111.96 (19.26)  Female: 124.02 (25.38) | Total: 26.82 (7.56)  Male: 23.76 (6.3)  Female:28.98 (7.74) | - | Total: 28.08 (15.84)  Male: 30.06 (17.46)  Female:26.82 (14.22) | - | - | - | Verbal  Males: 53.38 (9.76)  Females: 50.26 (9.45)  Spatial  Males: 53.63 (10.74)  Females: 49.22 (9.92)  Memory  Males: 50.70 (10.93)  Females: 52.83 (10.96)  Perceptual Speed  Males: 49.19 (10.52)  Females: 49.73 (10.56)  General Cognitive Ability  Males: 53.14 (10.74)  Females: 51.74 (10.46) |
| Salama et al. | **2019** | Total: 189.5 (14.2) | Total: 46.3 (13.6) | Total: 125.1 (20.3) | Total: 120.7 (49) | - | - | - | - |
| Salzwedel et al. | **2019** | - | - | - | - | Total = 261 | - | - | *Global Cognition* MoCA (Total): Cardiac Rehabilitation admission = 25.3 (3); Cardiac Rehabilitation discharge = 25.7 (2.9) |
| Shi et al. | **2019** | - | - | Total: 114.61 (35.06)  Males  White: 116.97 (33.74)  Black: 108.02 (35.82)  Females  White: 116.04 (33.91)  Black: 112.95 (37.81) | - | - | - | - | *Global Cognition*  Total: 0.52 (7.68)  Males: White: 0.71 (6.91), Black: -3.80 (6.39)  Females: White: 2.98 (6.8), Black: -1.36 (7.32)  *Verbal Memory*  *Logical memory l*  Total: 20.00 (10.00)  Males: White: 20.00 (9.00), Black: 17.00 (10.00)  Females: White: 22.00 (9.00), Black: 18.00 (8.00)  *Logical Memory ll*  Total: 16.00 (10.00)  Males: White: 16.00 (10.00), Black: 12.00 (8.00)  Females: White: 18.00 (9.00), Black: 14.00 (8.00)  *Logical Memory ll-recognition*  Total: 24.00 (4.00)  Males: White: 24.00 (4.00), Black: 22.00 (5.00)  Females: White: 25.00 (4.00), Black: 23.00 (4.00)  *Attention*  *DSF*  Total: 11.00 (4.00)  Males: White: 12.00 (4.00), Black: 10.50 (4.00)  Females: White: 12.00 (4.00), Black: 11.00 (4.00)  *DSB*  Total: 7.00 (3.00)  Males: White: 8.00 (4.00), Black: 6.00 (3.00)  Females: White: 8.00 (4.00), Black: 7.00 (2.00)  *Processing Speed*  *Digit Coding*  Total: 60.00 (24.00)  Males: White: 57.00 (20.00), Black: 45.00 (20.00)  Females: White: 67.00 (23.00), Black: 59.00 (25.00)  *TMT-A*  Total: 0.42 (0.21)  Males: White: 0.43 (0.20), Black: 0.48 (0.25)  Females: White: 0.39 (0.18), Black: 0.44 (0.21)  *Executive Function*  *TMT-B*  Total: 0.91 (0.53)  Males: White: 0.89 (0.48), Black: 1.16 (0.67)  Females: White: 0.83 (0.44), Black: 1.01 (0.57)  *Word Reading*  Total: 42.00 (13.00)  Males: White: 45.00 (10.00), Black: 36.00 (16.00)  Females: White: 46.00 (9.00), Black: 37.00 (13.00) |
| Sierra et al. | **2004** | Without WML (Total): 95.4 (16.2), N = 37 With WML (Total): 93.6 (14.4), N = 23 | - | - | - | - | - | - | *Attention* Digit Span Forward Test (Total): Diabetes = 5.51 (0.97), No Diabetes = 4.86 (1.14)  *Intelligence* WAIS (Total): Without WML = 96 (21), With WML = 103 (26)  *Working Memory* Digit Span Backwards (Total): Without WML = 4.12 (0.99), With WML = 4 (0.43) |
| Singh-Manoux et al. | **2009** | CHD (Total): 109.8 (19.8), N = 181 No CHD (Total): 106.2 (18), N = 2111 | - | - | - | - | - | - | *Verbal Memory* Delayed Recall (Total): 6.9 (2.4)  *Inductive Reasoning* AH-4 (Total): 47 (10.9) |
| Solomon et al. | **2009** | Total:  Low TC = 84.6 (7.2), N = 123  Intermediate TC = 104.4 (5.4), N = 365  High TC = 133.2 (16.2), N = 894 | - | - | - | Total:  Low TC = 3  Intermediate TC = 16  High TC =173 | -- | - | *Episodic memory* Summary Score: Total = 5  *Global Cognition* MMSE (Total): 26 |
| Suvila et al. | **2021** | Total = 90 (18), N = 2946 | Total: 28.8 (9), N = 2561 | - | - | - | - | - | *Verbal Memory* RAVLT (Summary Metric - Total): 8.5 (3.4)  *Working Memory* DSST (Total): 68 (17)  *Global Cognition* MoCA (Total): 23.9 (3.9) |
| Szczesnia et al. | **2020** | - | - | - | - | - | - | Baseline, 3-, and 6-year follow up (Total):  Male = 105  Female = 231  Total = 336 | *Attention* TMT-A (Total): Healthy = 34.03 (10.38); Microbleeds = 35.14 (10.07); Low WMH = 37.27 (11.72); High WMH = 41.80 (13.71); Infarcts = 44.06 (13.98)  *Working Memory* DSST (Total): Healthy = 67.67 (15.37); Microbleeds = 69.36 (15.4); Low WMH = 63.76 (13.67); High WMH = 55.93 (12.37); Infarcts = 57.94 (16.63)  *Global Cognition* MoCA (Total): Healthy = 26.86 (2.5); Microbleeds = 26.5 (1.7); Low WMH = 26.14 (2.5); High WMH = 25.2 (2.62); Infarcts = 25.12 (3.07) |
| Szoeke et al. | **2016** | Total (Baseline) = 104.94 (18.9), N = 387 | Total (Baseline) = 27.9 (7.56), N = 387 | - | Total (Baseline) = 23.94 (15.3), N = 387 | - | - | - | *Verbal Memory* CERAD (Delayed - Total): 7.67 (1.53) CERAD (Immediate - Total): 7.24 (1.1) CVLT (Immediate - Total): 8.77 (2.06) CVLT (Delayed - Total): 8.81 (3.23) |
| Szoeke et al. | **2019** | - | - | - | - | - | Total (Females): n = 5 | - | - |
| Tufvesson et al. | **2013** | - | Total (Baseline) = 24.84 (6.66), N = 864 | Total (Baseline) = 75.6 (18.18), N = 864 | Total (Baseline) = 23.94 (11.92), N = 864 | - | - | - | *Global Cognition* MMSE (Total): 28.1 (1.8) |
| Tuligenga et al. | **2014** | Normoglycemia: 106.8 (18.9)  Pre-DM: 110.16 (19.08)  Newly Diagnosed DM: 110.16 (20.16)  Known DM: 106.56 (18.54) | - | - | - | - | Normoglycemia: n = 122  Pre-DM: n = 22  Newly Diagnosed DM: n = 9  Known DM: n = 14 | - | - |
| Vadini et al. | **2020** | Pre-liraglutide: 178  Pre-Lifestyle: 167.5 | Pre-liraglutide: 47  Pre-Lifestyle: 42 | - | Pre-liraglutide: 122.5  Pre-Lifestyle: 90 | Pre-liraglutide: n = 7  Pre-Lifestyle: n= 9 | - | - | Pre-lifestyle; Post-lifestyle; Pre-liraglutide; Post-liraglutide  Trail Making A z 0.69(0.43) 0.72 (0.56) 0.06 0.750 0.82 (0.63) 0.76 (0.58) 0.31 0.813 0.700  Trail Making B z 0.41 (1.0) 0.30 (1.1) 0.10 0.450 0.38 (0.7) 0.41 (0.77) 0.04 0.935 0.768  Digit span z 0.39 (1.25) 0.29 (0.89) 0.09 0.745 −0.06 (1.1) 0.80 (1.0) 0.82 0.024 0.041  Visual search matrices z −0.14 (0.84) 0.36 (0.74) 0.63 0.006 −0.31 (0.75) −0.20 (0.92) 0.13 0.500 0.111  Rey–Osterrieth complex figure (direct) z −0.52 (1.30) −0.56 (1.0) 0.03 0.883 0.20 (0.90) −0.06 (1.47) 0.21 0.347 0.578  Rey–Osterrieth complex figure (delayed) z −1.35 (1.13) −1.20 (1.54) 0.11 0.543 −1.28 (1.18) −0.73 (1.31) 0.44 0.098 0.307  Phonemic verbal fluency z −0.21 (1.21) 0.12 (1.28) 0.26 0.124 0.06 (1.22) −0.04 (1.32) 0.07 0.466 0.087  Domain z score  Memory z −0.48 (1.02) −0.45 (0.82) 0.03 0.901 −0.68 (0.77) 0.032 (0.80) 0.90 0.006 0.032  Attention z 0.28 (0.51) 0.54 (0.47) 0.53 0.036 0.23 (0.49) 0.29 (0.47) 0.12 0.619 0.209  Executive z 0.09 (0.96) 0.20 (1.04) 0.10 0.387 0.21 (0.78) 0.18 (0.88) 0.61 0.845 0.467 |
| Veugen et al. | **2018** | Total = 95.4 (21.6), N = 1135 | Total = 27.54 (8.64), N = 1135 | Total = 55.98 (18.54), N = 1135 | Total = 21.6, N = 1135 | - | Total = 397 | - | *Global Cognition* MMSE (Total): 29 (1.2) |
| Walker et al. | **2019** | 1st Quartile (Total): 205.6 (38.2), N = 3012 2nd Quartile (Total): 209.4 (38.2), N = 3001 3rd Quartile (Total): 211.7 (38.5), N = 2995 4th Quartile (Total): 212.4 (41.5), N = 2966 | 1st Quartile (Total): 52.3 (17.4), N = 3012 2nd Quartile (Total): 50.1 (16.4), N = 3001 3rd Quartile (Total): 48.5 (16.4), N = 2995 4th Quartile (Total): 46.8 (16), N = 2966 | 1st Quartile (Total) = 128.6 (36), N = 3012 2nd Quartile (Total) = 133.1 (35.8), N = 3001  3rd Quartile (Total) = 135.3 (35.7), N = 2995 4th Quartile (Total) = 136.4 (37.7), N = 2966 | - | - | 1st Quartile (Total): Hypertension = 151 2nd Quartile (Total): Hypertension = 187 3rd Quartile (Total): Hypertension = 190 4th Quartile (Total): Hypertension = 252 | 1st Quartile (Total): Hypertension = 3012 2nd Quartile (Total): Hypertension = 3001 3rd Quartile (Total): Hypertension = 2995 4th Quartile (Total): Hypertension = 2966 | - |
| Wang et al. | **2016** | Total = 93.96 (27.54), N = 3048 | Total = 26.1 (8.82), N = 3048 | Total = 45.54 (21.06), N = 3048 | Total = 30.24 (29.7), N = 3048 | - | - | Total = 1582 | - |
| Wang et al., | **2018** | Total = 210 (39), N = 13,720 | Total = 50 (17), N = 13,720 | - | - | - | - | Total = 864 | - |
| Wendell et al. | **2014** | Total: 201 (36.2) | - | - | - | - | Total: n = 66 | - | - |
| Wharton et al., | **2014** | - | Total = 64.66 (17.13), N = 571 | Total = 128.99 (29.73), N = 571 | Total = 91.75 (51.41), N = 571 | - | - | - | - |
| Wieczorek et al. | **2016** | - | - | - | - | - | - | Total = 50 | *Global Cognition* MMSE (Total): 29 |
| Wu et al. | **2022** | - | - | - | - | Diabetes-Free: n = 509  Treated Diabetes: n = 79  Untreated Diabetes: n = 92 | - | - | - |
| Yaffe et al. | **2014** | Total (Baseline) = 177.4 (33.1), N = 3381 | - | - | - | - | - | - | *Verbal Memory* RAVLT (Delayed Recall - Total): 8.3 (3.3)  *Working Memory* DSST (Total): 69.9 (16.2) |
| Yang et al. | **2018** | - | - | - | - | - | - | Total = 575 | *Verbal Memory* RAVLT (Delayed Recall - Total): 9.8 (3.4) RCF (Delated Recall - Total): 21.1 (7.6)  A*ttention* TMT-A (Total): 25.8 (10.3)  *Psychomotor Speed* SDMT (Total): HIV Uninfected = 53.7 (12.8) |
| Yano et al. | **2018** | Visit 1 (Total) = 214.5 (41.1), N = 11,408 | Visit 1 (Total) = 52.1 (17), N = 11,408 | - | - | - | - | - | *Verbal Memory* Delayed Recall (Total): 5.2 (1.9)  *Working Memory* DSST (Total): 43.7 (13.4) |
| Yano et al. | **2014** | Baseline (Y0 - Total) = 177.7 (32.8), N = 2326 | Baseline (Y0 - Total) = 53.3 (12.6), N = 2326 | - | - | - | - | - | - |
| Ylilauri et al. | **2017** | Cholesterol intake quartile, mg/d: 1 (<331) Total: 104.22 (19.08) Cholesterol intake quartile, mg/d: 2 (331–387) Total: 106.02 (19.26) Cholesterol intake quartile, mg/d: 3 (388–458) Total: 106.92 (20.34) Cholesterol intake quartile, mg/d: 4 (>458) Total: 108.36 (18.72) Egg intake quartile, g/d: 1 (<14) Total: 135 (17) Egg intake quartile, g/d: 2 (14–25) Total: 106.74 (18.9) Egg intake quartile, g/d: 3 (26–43) Total: 104.94 (19.8) Egg intake quartile, g/d: 4 (>43) Total: 106.2 (17.82) | Cholesterol intake quartile, mg/d: 1 (<331) Total: 22.68 (5.04) Cholesterol intake quartile, mg/d: 2 (331–387) Total: 23.22 (5.58) Cholesterol intake quartile, mg/d: 3 (388–458) Total: 23.22 (5.4) Cholesterol intake quartile, mg/d: 4 (>458) Total: 23.76 (5.58) Egg intake quartile, g/d: 1 (<14) Total: 23.04 (5.58) Egg intake quartile, g/d: 2 (14–25) Total: 23.04 (5.58) Egg intake quartile, g/d: 3 (26–43) Total: 23.22 (5.04) Egg intake quartile, g/d: 4 (>43) Total: 23.76 (5.58) | Cholesterol intake quartile, mg/d: 1 (<331) Total = 70.56 (17.46) Cholesterol intake quartile, mg/d: 2 (331–387) Total = 72.54 (18.18) Cholesterol intake quartile, mg/d: 3 (388–458) Total = 73.62 (19.26) Cholesterol intake quartile, mg/d: 4 (>458) Total = 74.7 (17.82) Egg intake quartile, g/d: 1 (<14) Total = 73.44 (19.44) Egg intake quartile, g/d: 2 (14–25) Total = 73.44 (18) Egg intake quartile, g/d: 3 (26–43) Total = 72.18 (18.72) Egg intake quartile, g/d: 4 (>43) Total = 72.54 (16.92) | Cholesterol intake quartile, mg/d: 1 (<331) Total: 25.74 (15.66) Cholesterol intake quartile, mg/d: 2 (331–387) Total: 23.76 (13.68) Cholesterol intake quartile, mg/d: 3 (388–458) Total: 22.5 (12.78) Cholesterol intake quartile, mg/d: 4 (>458) Total: 22.5 (16.74) Egg intake quartile, g/d: 1 (<14) Total: 25.74 (16.38) Egg intake quartile, g/d: 2 (14–25) Total: 23.58 (13.5) Egg intake quartile, g/d: 3 (26–43) Total: 23.04 (12.96) Egg intake quartile, g/d: 4 (>43) Total: 22.14 (16.2) | - | - | - | *Verbal Memory* Selective Reminding Test (Total): Cholesterol intake quartile, mg/d: <339 (284) = 34.5 Cholesterol intake quartile, mg/d: 339–406 (370) = 34.4 Cholesterol intake quartile, mg/d: >406 (456) = 34 Egg intake quartile, g/d: <16 (8) = 34 Egg intake quartile, g/d: 16–32 (23) =34  Egg intake quartile, g/d: >32 (45) = 34.9  *Working Memory* Visual Reproduction Test (Total):  Cholesterol intake quartile, mg/d: <339 (284) = 11.3 Cholesterol intake quartile, mg/d: 339–406 (370) = 11.3 Cholesterol intake quartile, mg/d: >406 (456) = 11.3 Egg intake quartile, g/d: <16 (8) = 11.2 Egg intake quartile, g/d: 16–32 (23) =11.1 Egg intake quartile, g/d: >32 (45) = 11.6  *Global Cognition* MMSE (Total): Cholesterol intake quartile, mg/d: <339 (284) = 27.1 Cholesterol intake quartile, mg/d: 339–406 (370) = 27 Cholesterol intake quartile, mg/d: >406 (456) = 27.1 Egg intake quartile, g/d: <16 (8) = 27 Egg intake quartile, g/d: 16–32 (23) = 27.1 Egg intake quartile, g/d: >32 (45) = 27.2 |
| Young et al. | **2006** | - | - | - | - | - | - | Total (Baseline) = 3811 | *Verbal Memory* Delayed Recall (Total) With Hyperinsulinemia Fasting insulin (cutoff 75th percentile) = 6.58 (1.42) Fasting insulin (cutoff 12.2 mU/l) = 6.59 (1.4) HOMA (cutoff 75th percentile) = 6.56 (1.41) HOMA (cutoff 2.6) = 6.64 (1.41)  Without Hyperinsulinemia Fasting insulin (cutoff 75th percentile) = 6.83 (1.42) Fasting insulin (cutoff 12.2 mU/l) = 6.85 (1.43) HOMA (cutoff 75th percentile) = 6.82 (1.43) HOMA (cutoff 2.6) = 6.86 (1.43)  *Working Memory* DSST (Total):  With Hyperinsulinemia Fasting insulin (cutoff 75th percentile) = 43 (13.7) Fasting insulin (cutoff 12.2 mU/l) = 43.7 (13.7) HOMA (cutoff 75th percentile) = 42.8 (13.8) HOMA (cutoff 2.6) = 44.4 (13.7)  Without Hyperinsulinemia Fasting insulin (cutoff 75th percentile) = 47.9 (13.1) Fasting insulin (cutoff 12.2 mU/l) = 48.1 (13.1) HOMA (cutoff 75th percentile) = 47.8 (13.2) HOMA (cutoff 2.6) = 48.4 (13) |

**Supplemental Table B.7.** List of Cognitive tests used across all cognitive domains.

| Cognitive Variable | Cognitive Test(s) |
| --- | --- |
| Memory  *Verbal Memory* | Immediate and Delayed Verbal Memory, Spot the Word test, East Boston Memory test, Rey Auditory Verbal Learning Test (Immediate & Delayed recall, Learning & Summary Score), Selective Reminding Test, Rey–Osterreith complex figure (Immediate & Delayed), Consortium to Establish a Registry for Alzheimer’s Disease (Immediate & Delayed), California Verbal Learning Test (Immediate & Delayed), Word List Learning |
| *Working Memory* | Digit Symbol Substitution Test, Composite Score, Chinese Clinical Memory Scale, Digit Span Backwards, McNair Survey, WAIS-IV Digit Sequencing, Memory Index Score (MoCA), Visual Reproduction Test |
| Attention | Trail making Test Part A, Choice Reaction Time, Simple Reaction Time, Digit Span Forward Test, 5-Choice Movement Test |
| Intelligence | Wechsler Adult Intelligence Scale, IQ, Mental Rotation Test |
| Executive Function  *Letter Cancellation* | Letter Search Speed Test, Letter Cancellation Composite Score |
| *Verbal Fluency* | Word Fluency test, Boston Naming Test, Mill Hill Vocabulary Test, Phonemic Fluency Test, Semantic Fluency Test, Verbal Index Score, Benson Delay Test, Buschke Delay Test |
| *Processing Speed* | Trail-making Test Part-B, Trail-making Test Part B minus A, Stroop Test (Interference Time), Word Matching Test, Composite Executive Score, Rapid Visual Processing (Cambridge Neuropsychological Test Automated Battery & Isolated), Stroop Colour Word Test, Executive Index Score (MoCA), Visual Search Speed, Labyrinth Test |
| Global Cognition | Mini-Mental State Exam, Montreal Cognitive Assessment, Informant Questionnaire on Cognitive Decline in the Elderly, Cambridge Cognition Examination, National Adult Reading Test, Multilingual Naming Test, Intelligenz-Struktur-Test, Børge Priens Prøve, Addenbrooke’s cognitive examination, U.S. Health and Retirement Study Composite Score, Consortium to Establish a Registry for Alzheimer’s Disease |
| Inductive Reasoning | Alice Heim 4-I |
| Psychomotor Speed | Symbol Digits Modalities Test |
| Visuospatial Organisation | Block Design Test, Visual Index Score (MoCA), Clock Drawing Test |
